# PolyGA targets the ER stress-adaptive response by impairing GRP75 function at the MAM in *C9ORF72*-ALS/FTD

**DOI:** 10.1007/s00401-022-02494-5

**Published:** 2022-09-19

**Authors:** Federica Pilotto, Alexander Schmitz, Niran Maharjan, Rim Diab, Adolfo Odriozola, Priyanka Tripathi, Alfred Yamoah, Olivier Scheidegger, Angelina Oestmann, Cassandra N. Dennys, Shrestha Sinha Ray, Rochelle Rodrigo, Stephen Kolb, Eleonora Aronica, Stefano Di Santo, Hans Rudolf Widmer, Nicolas Charlet-Berguerand, Bhuvaneish T Selvaraj, Siddharthan Chandran, Kathrin Meyer, Benoît Zuber, Anand Goswami, Joachim Weis, Smita Saxena

**Affiliations:** 1 Department of Neurology, Inselspital, University Hospital, Bern, Switzerland; 2 Department for BioMedical Research, University of Bern, Bern, Switzerland; 3 Institute of Anatomy, University of Bern, Bern, Switzerland; 4 Institute of Neuropathology, RWTH Aachen University Hospital, Aachen, Germany; 5 Center for Gene Therapy, The Research Institute at Nationwide Children’s Hospital, Columbus, OH USA; 6 Department of Pediatrics, The Ohio State University, Columbus, OH USA; 7 Department of Neurology and Biological Chemistry and Pharmacology, The Ohio State University, Columbus, OH USA; 8 Department of (Neuro) Pathology, Amsterdam UMC, University of Amsterdam, Amsterdam Neuroscience, Amsterdam, The Netherlands; 9 Department of Neurosurgery, Inselspital, University Hospital, Bern, Switzerland; 10 CNRS, Inserm, IGBMC UMR 7104, U 1258, Université de Strasbourg, F-67404 Illkirch, France; 11 UK Dementia Research Institute at University of Edinburgh, University of Edinburgh, Edinburgh, EH16 4SB UK; 12 Euan MacDonald Centre for MND Research, University of Edinburgh, Edinburgh, EH16 4SB UK; 13 Centre for Clinical Brain Sciences, University of Edinburgh, Edinburgh, EH16 4SB UK

## Abstract

ER stress signaling is linked to the pathophysiological and clinical disease manifestations in amyotrophic lateral sclerosis (ALS). Here, we have investigated ER stress-induced adaptive mechanisms in *C9ORF72*-ALS/FTD, focusing on uncovering early endogenous neuroprotective mechanisms and the crosstalk between pathological and adaptive responses in disease onset and progression. We provide evidence for the early onset of ER stress-mediated adaptive response in *C9ORF72* patient-derived motoneurons (MNs), reflected by the elevated increase in GRP75 expression. These transiently increased GRP75 levels enhance ER–mitochondrial association, boosting mitochondrial function and sustaining cellular bioenergetics during the initial stage of disease, thereby counteracting early mitochondrial deficits. In *C9orf72* rodent neurons, an abrupt reduction in GRP75 expression coincided with the onset of UPR, mitochondrial dysfunction and the emergence of PolyGA aggregates, which co-localize with GRP75. Similarly, the overexpression of PolyGA in *WT* cortical neurons or *C9ORF72* patient-derived MNs led to the sequestration of GRP75 within PolyGA inclusions, resulting in mitochondrial calcium (Ca^2+^) uptake impairments. Corroborating these findings, we found that PolyGA aggregate-bearing human post-mortem *C9ORF72* hippocampal dentate gyrus neurons not only display reduced expression of GRP75 but also exhibit GRP75 sequestration within inclusions. Sustaining high GRP75 expression in spinal *C9orf72* rodent MNs specifically prevented ER stress, normalized mitochondrial function, abrogated PolyGA accumulation in spinal MNs, and ameliorated ALS-associated behavioral phenotype. Taken together, our results are in line with the notion that neurons in *C9ORF72*-ALS/FTD are particularly susceptible to ER–mitochondrial dysfunction and that GRP75 serves as a critical endogenous neuroprotective factor. This neuroprotective pathway, is eventually targeted by PolyGA, leading to GRP75 sequestration, and its subsequent loss of function at the MAM, compromising mitochondrial function and promoting disease onset.

**Supplementary Information:**

The online version contains supplementary material available at 10.1007/s00401-022-02494-5.

## Introduction

Silent prodromal phases, with no overt symptoms, are a conserved feature of most neurodegenerative diseases (NDs) [29, 35]. Adaptive responses, through homeostatic and compensatory processes, are likely to predominate during these early phases and might well be sufficient to counteract disease-associated functional deficits and enable cells to cope with stress. However, chronic stress responses may eventually be detrimental to the cell [19, 29, 34, 35, 39]. Identification of early compensatory measures and the specific pathomechanisms impairing them is pivotal in finding valid temporal entry points to target the pathology.

During presymptomatic stages of amyotrophic lateral sclerosis (ALS), motoneurons (MNs) already exhibit early vulnerability to endoplasmic reticulum (ER) stress [57]. Initially, ER stress responses are physiologically adaptive enabling cells to cope with stress. However, the chronicity of the ER stress response is detrimental to the cell [19, 32, 34, 39, 57]. We studied the relatively common monogenic form of ALS and frontotemporal dementia (FTD) due to a hexanucleotide (G4C2) repeat expansion within the first intron of the *C9ORF72* gene [15, 43, 55]. In *C9ORF72*-ALS/FTD patient-derived MNs, decreased cell survival was linked to dysfunction in Ca^2+^ homeostasis, increased ER stress, reduced mitochondrial membrane potential as well as mitochondrial dysfunction [13, 14, 23, 37, 48, 66].

Three neurodegenerative mechanisms are implicated in *C9ORF72*-ALS/FTD pathology: loss of function due to reduced *C9ORF72* transcript and proteins levels [2], a gain of function due to the formation of RNA foci [46], and a gain of toxic function, due to the generation and accumulation of five different dipeptide repeat proteins (DPRs), via repeat-associated non-AUG (RAN) translation of hexanucleotide repeat sequences from both sense and antisense strands [20]. Of the five DPR species, there is evidence for a toxic gain-of-function role for PolyGA, PolyGR, and PolyPR aggregates in promoting neurodegeneration in affected brain areas [30, 36, 40]. Previous studies have established that the positively charged arginine-rich PolyGR and PolyPR proteins, and the highly insoluble PolyGA aggregates are extremely toxic in neurons [44]. PolyGA is the most prominently detected DPR in cytoplasmic inclusions [68], and it accumulates in p62/ubiquitin-positive inclusions in the brain and spinal cord of *C9ORF72*-ALS/FTD patients [44, 68]. PolyGA-overexpressing mice exhibit motor and cognitive deficits along with TDP-43 pathology, cerebellar atrophy, and astrogliosis [20]. PolyGA induces toxicity by promoting ER stress and activating caspase 3-related apoptotic pathways [44, 68]. PolyGA directly connects with and inhibits the proteasome, thereby promoting TDP-43 pathology [33, 68]. Moreover, PolyGA sequesters proteins, such as Unc119 [], a lipid-binding chaperon involved in vesicular and protein trafficking, contributing to selective neuronal vulnerability in *C9ORF72*-ALS/FTD [44]. In addition, HR23 proteins involved in proteasomal degradation together with proteins involved in nucleocytoplasmic transport were sequestered along with GA aggregates [67]. A recent study reported that PolyGA aggregates sequestered VCP, impairing autophagy [1]. Taken together, these results have suggested contrasting potential scenarios of how a *C9ORF72* hexanucleotide repeat expansion leads to neurodegeneration. However, the molecular pathomechanism by which PolyGA might influence early phases of MN degeneration, and in particular, regulation of early ER–mitochondrial adaptive response and signaling pathway has not been studied in detail thus far.

We here show that the initiation of ER-stress-induced compensatory responses involve the enhanced expression of glucose-regulated protein 75 (GRP75) at mitochondria-associated membranes (MAMs), counteracting early mitochondrial Ca^2+^ imbalance in human *C9ORF72*-ALS/FTD and *C9orf72* mouse neurons. This protracted pre-symptomatic phase is followed by the near-complete loss of GRP75 expression coinciding with the appearance of unfolded protein response (UPR) signaling, mitochondrial dysfunction, and appearance of PolyGA aggregates. PolyGA inclusions sequestered GRP75, leading to its reduction at the MAMs and eventual loss of function, and mitochondrial dysfunction. Comparable pathological sequestration of GRP75 in PolyGA aggregate-bearing neurons was observed in human *C9ORF72*-ALS/FTD post-mortem tissue. Sustaining high GRP75 expression in spinal *C9orf72* MNs specifically prevented ER stress and normalized mitochondrial function, suggesting that impairment of GRP75 function is a critical early event in *C9ORF72*-ALS/FTD development and progression.

## Methods

### Mouse strains

The *C9-500* BAC mouse line (FVB/NJ-Tg(C9orf72)500Lpwr/J) described in [41] were purchased from Jackson Laboratory (https://www.jax.org/strain/029099) and maintained in heterozygosis crossed with (non-carrier) mice FVB/NJ (Janvier labs, SC-FVBN-F). Within our colony, an acute phenotype is observed in 25–30% of females with a median life span of 105 days. The remaining female and male mice exhibit a slow progressing phenotype [53]. Note: some labs were not able to observe the original phenotype [51]. For all immunochemical analyses, only males were included and both males and females were examined together as from P125, to eliminate the acute phenotype, and to include only the slow progressive phenotype. All survival and behavior cohorts included both genders and slow and fast progressing females. Long range PCR was routinely performed to define repeat length-matched cohorts. All behavior and survival assays were performed in repeat length-matched mice. Adult C57BL/6 J mice were transduced with lentiviral-PolyGA. Animal care, ethical usage and procedures were in accordance with the Swiss Veterinary Law guidelines, and the study was approved by the animal commission of Canton of Bern, Switzerland.

### iPSC differentiation into iMNs

The *C9ORF72*-ALS/FTD iPSCs were obtained from Biomedicum Stem Cell Center, GoEditStem platform, HiLIFE, Helsinki, Finland, University of Edinburgh, UK, and the iPSC Core, Cedar Sinai, USA. iPSCs were cultured in GeltrexTM (ThermoFischer) coated plates in mTeSR^TM^1 (StemCell technologies) media. MN differentiation was adapted from previous studies [45, 63]. Briefly, human iPSCs were dissociated to single cells using Accutase (StemCell technologies) and seeded at 3 × 10^6^ onto 10 cm plate with N2B27 differentiation medium (Advanced DMEM/F12:Neurobasal (1:1) medium, 1% Pen/strep (Gibco), 1% GlutaMAX (Gibco), 0.1 mM 2-mercaptoethanol (Gibco), 1 × B27 supplement (Gibco), 1 × N2 supplement (Gibco)), supplemented with 10 ng/mL basic fibroblast growth factor ((StemCell technologies), 20 µM SB431542 (StemCell technologies), 0.1 µM LDN193189 (StemCell technologies), 3 µM CHIR99021 (StemCell technologies), 10 µM L-Ascorbic Acid (L-AA; Sigma) and 1 × Revitacell supplement (Gibco)) to initiate embryoid bodies (EBs) formation. On day 2, media patterning of EBs was induced by adding media supplemented with 100 nM all-trans retinoic acid (RA; sigma) and 500 nM Smoothened Agonist (SAG; StemCell technologies). EBs were pelleted and fed with fresh media on every alternate day until day 14. 10 ng/mL Brain derived neurotrophic factor (BDNF; StemCell technologies) was added from day 7, while 10 µM DAPT (StemCell technologies) was added from day 9. EBs were dissociated using trypsin on day 16 and triturated with ice cold cell trituration and wash medium (PBS (Gibco), 0.45% Glucose, 0.1% Bovine Serum Albumin (BSA; Sigma), 2 mM MgCl2, 0.8 mM EDTA (Invitrogen), 2.5% Fetal Bovine Serum (FBS; Sigma), 1 × N2 supplement, 1 × B27 supplement and DNAse). Triturated EBs were plated on poly-ornithine/laminin (Sigma) coated plates in MN feeding medium Neurobasal medium (Gibco), 1 × glutaMAX, 1 × Non-essential amino acid (NEAA, Gibco), 0.1 mM 2-mercapthoethanol, 1 × N2 supplement, 1 × Pen/strep, 1 × B27 supplement, 10 ng/mL glial cell derived neurotrophic factor (GDNF; StemCell technologies), BDNF 10 ng/mL, 10 ng/mL insulin-like growth factor (IGF-1; StemCell technologies), 10 ng/mL Ciliary neurotrophic factor (CNTF; StemCell technologies), 100 nM RA and 10 µM AA and kept at 37 °C and 5% CO_2_ for maturation up to 4 weeks.

### Generation of direct-induced neurons (dNeus)

Fibroblast collection: skin punches were collected and fibroblasts were grown in fibroblast media (10% FBS (Gibco), 1% anti-anti (Gibco) in DMEM Glutamax (Gibco)) for up to 1 month, passaging once every 1–2 weeks. In addition, this study used a fibroblast sample from the NINDS Repository, as well as clinical data. NINDS Repository sample numbers corresponding to the samples used are: AG08620. Patient skin-derived fibroblasts were directly reprogramed into neural progenitor cells (NPCs) as previously described [50]. Briefly, 100,000–200,000 fibroblasts were seeded into a fibronectin (5 μg/mL, Millipore) coated 6-well plate and cultured in a 37 °C, 5% CO_2_ incubator. Next day, fibroblasts were transduced with a retroviruses for SOX2, cMyc, KLF4, and OCT3/4. 24 h later virus was removed and media replaced with fresh fibroblast media (10% FBS (Gibco), 1% anti-anti (Gibco) in DMEM Glutamax (Gibco)). After a 24-h rest period, media of transduced fibroblasts was changed to a neuralizing media (1% B27 (Gibco), 1% N2 (GIBCO), 1% anti-anti (Gibco), 20 ng/mL FGF2 (peprotech), 20 ng/mL EGF (peprotech), and 5 μg/mL heparin (Sigma)). Cells were cultured in this media until converted into NPC. NPCs were cultured in (1% B27 (Gibco), 1% N2 (Gibco), 1% anti-anti (Gibco), 20 ng/mL FGF2 (peprotech)). Induced direct neuron generation (dNeus): patient and healthy fibroblasts were directly converted to neurons using small molecules as previously described [28]. Briefly, 10 cm plates were coated overnight with polyornithine (10 μg/mL, Sigma) in borate buffer. Next day, plates were washed with DPBS and coated with laminin (5 μg/mL, Invitrogen) and fibronectin (2.5 μg/mL, Millipore Sigma) in DMEM/F12 at 37 °C for 2 h. Fibroblast cells (850,000) were seeded onto the plates in culture medium for 1 day. The cells were transferred to neuronal induction medium (DMEM/F12: Neurobasal (Gibco) [1:1] with 0.5% N-2 (Gibco), 1% B-27 (Gibco), 100 μM cAMP (Sigma), and 20 ng/mL bFGF (Peprotech)) with the following chemicals: VPA (0.5 mM, Sigma), CHIR99021 (3 μM, Axon medchem), repsox (1 μM, BioVision), forskolin (10 μM, Tocris), SP600125 (10 μM, Sigma), GO6983 (5 μM, Sigma) and Y-27632 (5 μM, Sigma). Half the medium was changed after 3 days with fresh induction medium. On the fifth day, cells were switched to neuronal maturation medium (DMEM/F12: Neurobasal [1:1] with 0.5% N-2 (Gibco), 1% B-27 (Gibco), 100 μM cAMP (Sigma), 20 ng/mL bFGF (Peprotech), 20 ng/mL BDNF (Gibco) and 20 ng/mL GDNF (Gibco)) with the following chemicals: CHIR99021 (3 μM), forskolin (10 μM) and SP600125 (10 μM).

### Intracerebroventricular (i.c.v.) viral injection

Unilateral injection of 1.5 μl of AAV6–GRP75 (viral titer: 1^12^–1^13^ gc/mL) in the left lateral ventricle was performed on neonatal mice (P1–P2), as previously described [16]. 0.1% fast green solution was added to the vector suspension, to visualize the spread of the virus.

### Stereotaxic lentiviral injection

A medial skin incision was performed on anesthetized animals to expose the skull, the incision extended to expose bregma and lambda. A driller was used to perform small holes on the skull surface. The following coordinates were used for LV::PolyGA-GFP/GFP injection: motor cortex AP + 1.5 ± 0.2, ML ± 1.5 ± 0.2, DV 2 ± 0.2, Ang 0°; AP + 0.8 ± 0.2, ML ± 1.5 ± 0.2, DV 1.8 ± 0.15, Ang 0°; hippocampus AP − 2.2 ± 0.2, ML ± 2 ± 0.4, DV 1.7 ± 0.2, Ang 0°; AP − 1.8 ± 0.2, ML ± 1 ± 0.4, DV 2 ± 0.2, Ang 0°. A 10 µL Hamilton syringe (Hamilton, 8314) was loaded with 1.0 µL of LV::PolyGA-GFP/LV::GFP and place into a nano injector (Pump 11 Elite, Harvard apparatus, 70–4507). Same coordinates as above were used for motor cortex and hippocampal injections of AAV6–GRP75 for MAM isolation. In addition, coordinates for injections into somatosensory cortex: AP − 0.5 ± 0.2, ML ± 1.5 ± 0.2 DV 1 ± 0.2, Ang 0°, and visual cortex: AP—3 ± 0.3, ML ± 2 ± 0.2, DV 1 ± 0.2, Ang 0°.The virus was injected at a rate of 150 nl/min, and the needle was left in place for 2 min before withdrawal. Next, the skin was sutured, animals were injected subcutaneously with warm saline and placed in a warm cage and monitored until fully awake.

### Immunofluorescence on iMNs

iMNs plated on coverslips were fixed using 4% paraformaldehyde (PFA) for 15 min and blocked for 1 h with 3% bovine serum albumin (BSA) and 0.1% TritonX-100 in phosphate buffered saline PBS. iMNs were incubated with the following primary antibody: mouse anti-GRP75 (Abcam, ab2799, 1:200), rabbit anti-GRP75 (1:200, Abcam, ab53098), rabbit anti-BiP (Abcam, ab21685, 1:500), goat anti-ChAT (Millipore, AB144P, 1:500), chicken anti-MAP2 (Sigma Aldrich, AB15452, 1:500), rat anti Isl1-2 (DSHB 39.4D5), mouse anti-HB9 (1:500, DSHB 81.5C10), mouse anti-TDP43 (ABCAM ab104223, 1:500), in blocking buffer overnight at 4 °C. After three washes with PBS, cells were incubated in blocking buffer with appropriate Alexa Fluor secondary antibodies and DAPI for 1 h at RT, followed by three PBS washes and coverslips were mounted on glass slides. Confocal images were acquired with FluoViewTM FV1000 (Olympus) fitted with a 20 × or 40 × air objective and 60 × immersion oil objective.

### Immunofluorescence and immunohistochemistry on rodent tissue

Mice were transcardially perfused with 4% PFA in PBS; brain, cerebellum and lumbar spinal cord were isolated and kept overnight at 4 °C in 4% PFA, followed by 30% sucrose in PBS for cryoprotection. After embedding in O.C.T compound (Bio system), spinal cord (50 µm), and brain (30 μm) sections were cut using a cryostat. Antibodies used for immunofluorescence were: rabbit anti-GRP78/BiP (1:500, Abcam, ab21685), mouse anti-KDEL/BiP (1:500, Enzo Life Science, SPA-827), rabbit anti-P_i_-eif2α (1:25, Cell Signaling, 3597L), rabbit anti P_i_-PERK (1:200, Abcam, ab192591), goat-anti ChAT (1:1000, Millipore, AB144P), mouse-anti myc (1:100, Cell Signaling, 2276S), mouse anti-GRP75 (1:200, Abcam, ab2799), rabbit anti-GRP75 (1:200, Abcam, ab53098), rabbit anti-IP_3_R (1:500, Abcam, ab5804), mouse anti-VDAC1 (1:500, Millipore, MABN504), rabbit anti-GFP (1:1000, Cell Signaling, 2956), mouse anti-GFP (1:1000, Abcam, ab1218) mouse anti-N-term PolyGA 1:1000, mouse anti-NeuN (1:500, Millipore, A60-MAB377). Heat-mediated antigen retrieval was performed using Sodium citrate buffer 10 mM pH 6 for Myc staining. Sections were kept for 2 h in PBS solution containing 0.05% Triton X-100 and 10% normal donkey serum ((NDS), Jackson immunoresearch). Antibodies were applied in PBS, 3% NDS, 0.05% Triton X-100, and incubated overnight (for brain) and for 2 days for spinal cord at 4 °C. Sections were briefly washed with PBS and incubated for 120 min at RT with appropriate secondary antibodies from Invitrogen. Heat-mediated antigen retrieval as above was performed on spinal cord sections for immunohistochemistry, then immersed in 3% H_2_O_2_ in PBS for 20 min to inhibit endogenous peroxidase activity. Blocking was done in PBS containing 0.05% Triton X-100 and 10% NDS for 2 h, incubated overnight at 4 °C with goat anti-ChAT (1:500, Millipore, AB144P) antibody diluted in the blocking solution. Next day, sections were incubated with the appropriate biotinylated secondary antibody (1:500) followed by 1 h incubation in PBS solution containing biotin–avidin complex (1:100, Vector Labs). Finally, 3,3′-diaminobenzidine (DAB) reaction was developed. The glass slides were dehydrated via ascending concentrations of EtOH and rinsed in xylene and mounted. Images were acquired using BX51 Olympus microscope.

### Imaging and image analysis

Confocal images were acquired using SP8 (Leica Microsystems) fitted with a 63 × oil objective, or FV1000 (Olympus) microscope, fitted with a 20 × , 40 × air objective or 60 × oil objective. All images were processed using Imaris software version 7.6.3 or Fiji. For the analysis of BiP, P_i_-eif2α, GRP75, labeling intensities, data were acquired using identical confocal settings, with signals at the brightest cells being non-saturated, and background levels outside MN pools still detectable. Images were analyzed quantitatively using FiJi or Imaris. Signal intensity values for the antigen of interest were calculated over several consecutive lumbar spinal cord Z-stack spaced 0.5 μm, after background subtraction from every channel. Lowest signals had values of below 50 and high-intensity neurons exhibited labeling values up to 255. Signal values below 50 in the case of BiP in *WT* animals were counted as basal expression. To count MNs numbers, cell counter plugin from Fiji was used. Imaris software was used to reconstruct the 3D isosurface for PolyGA aggregates volume. Fiji software was employed to analyze the mitochondria–ER contacts and sphericity for 3view EM images. SBF SEM volume reconstruction were done using the Volume viewer plug in from Fiji and IMOD software was used for the 3D segmentation of mitochondria.

### Human post-mortem tissue

The human post-mortem brain, hippocampus and lumbar spinal cord samples fixed in buffered formalin were obtained from the archives of the Department of (Neuro)Pathology (Amsterdam UMC, University of Amsterdam, The Netherlands), and included four *C9ORF72*-ALS/FTD, and four age-matched control cases. They were selected from a retrospective searchable neuropathological database that was reviewed independently by two neuropathologists (Eleonora Aronica and Dirk Troost), and included cases with consent for post-mortem brain/spinal cord autopsy and use of the autopsy tissue and their medical records for research purposes. All of the *C9ORF72-*ALS/FTD patients had shown clinical signs and symptoms of lower and upper MN disease. All the patients fulfilled the diagnostic criteria for ALS. The controls included in the present study were adult individuals without any history of neurological disease, based on their last clinical evaluation (Demographic information in Supplementary Table 2, online resource).

### Diaminobenzidine immunohistochemistry of human post-mortem tissue

Paraffin sections (3–4 µm) were placed on poly-l-lysine-coated slides and dried for 3–4 h on a heating plate at 40 °C. The sections were deparaffinized in xylene for 20 min and rehydrated in 100%, 95% and 70% EtOH for 5 min each, followed by endogenous peroxidase quenching (0.3% H_2_O_2_ in methanol) for 20 min. For antigen retrieval, the sections were heated in citrate buffer, pH 6 (Dako) for 20 min in a pressure cooker. After PBS washes, the sections were incubated with primary antibody (Supplementary Table 3, online resource) for 1 h at RT or at 4 °C overnight. After washing in PBS, they were incubated with the polymeric horseradish peroxidase linker secondary antibody (IL Immunologic) for 30 min at RT. For light microscopy, DAB reagent (DAB kits; DCS Innovative Diagnostic System) was used to stain the sections, and counter-stained with 6% haematoxylin for 3 min. Images of the DAB-stained sections were taken with a Zeiss Axioplan microscope equipped with a 40 × , 63 × objective and an Axiocam 506 color camera (Zeiss). Quantification was done manually using a 20 × lens on the DAB-stained sections from each case by counting the no. of α-MNs. Semi-quantitative assessment of GRP75 immunolabeling was performed on serial sections. Three lumbar spinal cord sections each from the four *C9ORF72*-ALS/FTD cases as well as four normal controls were examined. The level of GRP75 immunoreactivity in the majority (more than 95%) of the MNs of control cases were assigned as normal levels. Using a 10 × , 20 × objective, the number of MNs displaying GRP75 immunoreactivity were counted, and quantified based upon the observed GRP75 levels using the following scheme, A = MNs showing normal levels of GRP75, B = MNs showing moderate reduction of GRP75 levels, C = MNs showing strong reduction of GRP75 levels.

### Immunofluorescence labeling of human post-mortem tissue

Immunofluorescence labeling was performed as described previously [17, 64]. Briefly, deparaffinized tissue sections were boiled in citrate buffer, pH 6 (Dako) for 20 min in a pressure cooker for antigen retrieval. Sections were blocked with 10% normal goat serum (Life Technologies), for 1 h at RT, and incubated with the required primary antibody (Supplementary Table 3, online resource), at a dilution of 1:100 for each antibody at RT for 1 h or at 4 °C overnight. After washing in TBST for 10 min the sections were incubated with Alexa Fluor secondary antibodies (dilution 1:500 in PBS) at RT for 2 h. Sections were washed in TBST (2 × 10 min), and stained for 10 min with 0.1% Sudan Black in 80% EtOH to suppress endogenous lipofuscin auto-fluorescence and washed for 5 min in TBST and mounted with Vectashield mounting medium (Vector Laboratories) containing DAPI. Images were obtained with a LSM 700 (Zeiss) microscope using 40 × and 63 × objectives. Images were acquired by averaging 4 scans/area of interest resulting in an image size of 1024 × 1024 pixels. The laser intensity was kept constant for all the sections examined. Images were analysed using Adobe Photoshop CS5 and ZEN (Blue edition) 2009 software. For quantification of GRP75 immunofluorescence intensity in MNs, ROIs were manually defined and average intensity was measured using Fiji software. GRP75 intensities were binned into three classes (normal, medium reduction, and strong reduction) based on pixel intensity and graphs were plotted as percentage of MNs expressing different GRP75 levels. GRP75 sequestration by PolyGA in hippocampal dentate gyrus neurons was measured using IMARIS, wherein neurons harbouring PolyGA aggregates were analysed for GRP75 immunoreactivity either surrounding or colocalizing within aggregates. Graph was plotted as percentage of neurons, harbouring PolyGA aggregates exhibiting sequestration or devoid of GRP75 sequestration.

### Statistical analysis

Analysis were done using GraphPad Prism 6.0. Statistical significances were evaluated by two-tailed, unpaired Student’s *t* test and one way ANOVA. Post ANOVA Sidak test was used to evaluate statistical significance as indicated in the respective figure legend. Values are expressed as mean ± standard error of the mean (SEM) or standard deviation (SD) as indicated in the corresponding legends. *P < 0.05, **P < 0.01, ***P < 0.001 throughout the manuscript.

Additional methods are described in detail in Supplementary Information.

## Results

### *C9ORF72* patient-derived neurons exhibit early ER stress-mediated adaptive response

**Fig. 1:**
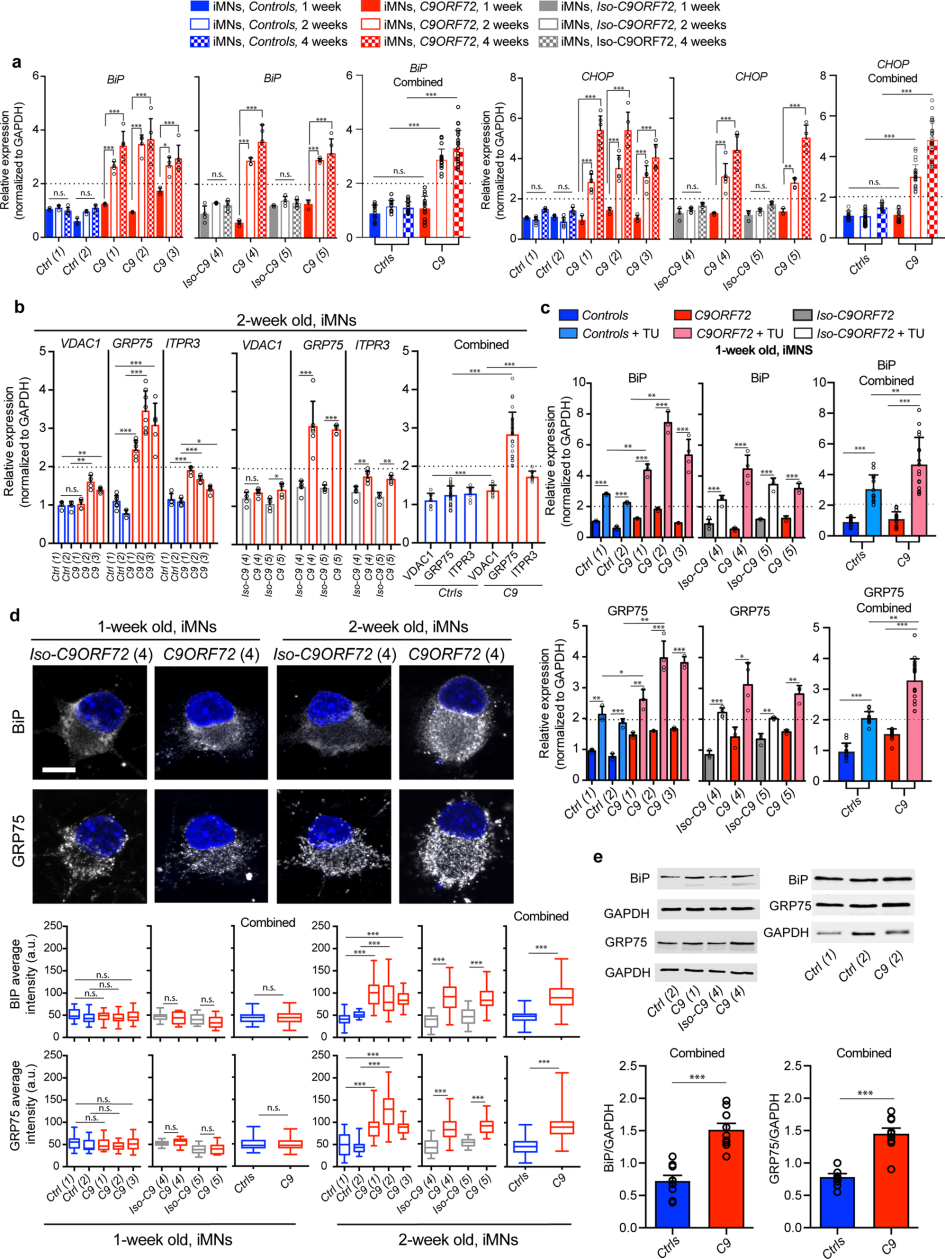
*C9ORF72* iMNs display ER stress-mediated increase in GRP75 expression. **a** qPCR analysis of IPSC-derived motoneurons (iMNs) after 1, 2 and 4 weeks of maturation from five different ALS patient lines (*C9(1),(2),(3),(4),(5)*), displaying increased levels of ER stress markers (*BiP* and *CHOP*) at 2 and 4 weeks, but not at 1 week compared to two healthy control lines (*Ctrl(1) and (2))* or corresponding isogenic control (*Iso-C9(4)*, *Iso-C9(5)*). One-way ANOVA; *BiP: Controls*: *F* = 38.94***, Sidak’s multiple comparison test: *Ctrl(1)* 1 week vs *C9(1)* 1 week n.s.; *Ctrl(2)* 1 week vs *C9(2)* 1 week n.s.; *Ctrl(1)* 1 week vs *C9(3)* 1 week n.s.; *C9(1)* 1 week vs *C9(1)* 2 and 4 weeks***; *C9(2)* 1 week vs *C9(2)* 2 and 4 weeks***; *C9(3)* 1 week vs *C9(3)* 2 weeks*; *C9(3)* 1 week vs *C9(3)* 4 week***. One-way ANOVA *BiP Isogenic*: *F* = 46.01***, Sidak’s multiple comparison test: *Iso-C9(4)* 1 week vs *C9(4)* 1 week n.s.; *Iso-C9(5)* 1 week vs *C9(5)* 1 week n.s.; *C9(4)* 1 week vs *C9(4)* 2 and 4 weeks***; *C9(5)* 1 week vs *C9(5)* 2 and 4 weeks***. One-way ANOVA; *CHOP: Controls*: *F* = 63.65***, Sidak’s multiple comparison test: *Ctrl(1)* 1 week vs *C9(1)* 1 week n.s.; *Ctrl(2)* 1 week vs *C9(2)* 1 week n.s.; *Ctrl(1)* 1 week vs *C9(3)* 1 week n.s.; *C9(1)* 1 week vs *C9(1)* 2 and 4 weeks***; *C9(2)* 1 week vs *C9(2)* 2 and 4 weeks***; *C9(3)* 1 week vs *C9(3)* 2 and 4 weeks***. One-way ANOVA *CHOP Isogenic*: *F* = 40.15***, Sidak’s multiple comparison test: *Iso-C9(4)* 1 week vs *C9(4)* 1 week n.s.; *Iso-C9(5)* 1 week vs *C9(5)* 1 week n.s.; *C9(4)* 1 week vs *C9(4)* 2 and 4 weeks***; *C9(5)* 1 week vs *C9(5)* 2 weeks**; *C9(5)* 1 week vs *C9(5)* 4 weeks***. Combined graph represents the average values for *Ctrls* lines (*Cntrl 1–2, Iso-C9 4–5*) and *C9* (*C9 1–2–3–4–5*), Unpaired *t* test; *BiP*: *Ctrls* 1 week vs *C9* 1 week *t* = 1.334, *P* = 0.1935, n.s.; *Ctrls* 2 weeks vs *C9* 2 weeks *t* = 16.59, *P *< 0.0001, ***; *Ctrls* 4 weeks vs *C9* 4 weeks *t* = 15.04, *P* < 0.0001, ***; *CHOP*: *Ctrls* 1 week vs *C9* 1 week *t* = 0.6029, *P* = 0.5512, n.s.; *Ctrls* 2 weeks vs *C9* 2 weeks *t* = 14.84, *P* < 0.0001, ***; *Ctrls* 4 weeks vs *C9* 4 weeks *t* = 15.40, *P* < 0.0001, ***. Values were normalized to relative expression of *GAPDH*. qPCR graphs plotted with s.d., *n* = 3–8 independent qPCR experiments repeated in triplicate. **b** qPCR analysis of mitochondria associated membrane (MAM) molecules *GRP75* and its binding partners *ITP3R* and *VDAC1* from 2-week-old iMNs. All *C9ORF72* patient lines display significant increase in *GRP75* transcript. (Unpaired *t* test; *VDAC1*: *Ctrl(1)* vs *C9(1)* *t* = 0.4234, *P* = 0.6896, n.s.; *Ctrl(2)* vs *C9(2)* *t* = 5.720, *P* = 0.0012**; *Ctrl(1)* vs *C9(3)* *t* = 6.024, *P* = 0.0018**; Iso-*C9(4)* vs *C9(4)* *t* = 1.843, *P* = 0.1026 n.s.; Iso-*C9(5)* vs *C9(5)* *t* = 3.090, *P* = 0.0214*. Unpaired *t* test; *GRP75*: *Ctrl(1)* vs *C9(1)* *t* = 12.21, *P* < 0.0001***; *Ctrl(2)* vs *C9(2)* *t* = 8.460, *P* < 0.0001***; *Ctrl(1)* vs *C9(3)* *t* = 9.333, *P* < 0.0001***; Iso-*C9(4)* vs *C9(4)* *t* = 5.128, *P* = 0.0006***; Iso-*C9(5)* vs *C9(5)* *t* = 19.32, *P* < 0.0001***. Unpaired *t* test; *ITPR3*: *Ctrl(1)* vs *C9(1)* *t* = 7.679, *P* = 0.0003***; *Ctrl(2)* vs *C9(2)* *t* = 8.262, *P* = 0.0002***; *Ctrl(1)* vs *C9(3)* *t* = 2.543, *P* = 0.0439*; Iso-*C9(4)* vs *C9(4)* *t* = 3.726, *P* = 0.0074**; Iso-*C9(5)* vs *C9(5)* *t* = 5.470, *P* = 0.0016**). Combined graph for *Ctrls* lines (*Ctrl 1–2, Iso-C9 4–5*) and *C9* (*C9 1–2–3–4–5*), Unpaired *t* test; *VDAC1*: *Ctrls* 2 weeks vs *C9* 2 weeks *t* = 4.026, *P* = 0.0003, ***; *GRP75*: *Ctrls* 2 weeks vs *C9* 2 weeks *t* = 12.06, *P* < 0.0001, ***; *ITP3R*: *Ctrls* 2 weeks vs *C9* 2 weeks *t* = 7.830, *P* < 0.0001, ***. **c** 1-week-old iMNs treated with TU (1 µg/mL) for 18 h display significant increase in transcripts of *BiP* as well as *GRP75*, increased expression was not only found in *C9ORF72* patient lines but also in *Controls* and isogenic lines. Unpaired *t* test; *BiP*: *Ctrl(1)* vs *Ctrl(1)* + TU *t* = 13.90, *P* = 0.0002***; *Ctrl(2)* vs *Ctrl(2)* + TU *t* = 20.24, *P* < 0.0001***; *C9(1)* vs *C9(1)* + TU *t* = 33.32, *P* < 0.0001***; *C9(2)* vs *C9(2)* + TU *t* = 7.267, *P* = 0.0008***; *C9(3)* vs *C9(3)* + TU *t* = 13.29, *P* = 0.0002***; *Ctrl(1)* + TU vs *C9(1)* + TU *t* = 6.460, *P* = 0.0030**; *Ctrl(2)* + TU vs *C9(2)* + TU *t* = 0.0038, *P* = 0.0038** Iso-*C9(4)* vs Iso-*C9(4)* + TU *t* = 7.330, *P* = 0.0003***; *C9(4)* vs *C9(4)* + TU *t* = 8.578, *P* = 0.0001***; Iso-*C9(5)* vs Iso-*C9(5)* + TU *t* = 9.384, *P* = 0.0007***; *C9(5)* vs *C9(5)* + TU *t* = 8.913, *P* = 0.0009***. Unpaired *t* test *GRP75*: *Ctrl(1)* vs *Ctrl(1)* + TU *t* = 7.643, *P* = 0.0016**; *Ctrl(2)* vs *Ctrl(2)* + TU *t* = 11.02, *P* = 0.0004***; *C9(1)* vs *C9(1)* + TU *t* = 5.953, *P* = 0.0040**; *C9(2)* vs *C9(2)* + TU *t* = 7.427, *P* = 0.0007***; *C9(3)* vs *C9(3)* + TU *t* = 18.03, *P* < 0.0001***; *Ctrl(1)* + TU vs *C9(1)* + TU *t* = 4.218, *P* = 0.0135*; *Ctrl(2)* + TU vs *C9(2)* + TU *t* = 6.485, *P* = 0.0013**. Iso-*C9(4)* vs Iso-*C9(4)* + TU *t* = 12.32, *P* < 0.0001***; *C9(4)* vs *C9(4)* + TU *t* = 3.749, *P* = 0.0133*; Iso-*C9(5)* vs Iso-*C9(5)* + TU *t* = 6.160, *P* = 0.0035**; *C9(5)* vs *C9(5)* + TU *t* = 7.524, *P* = 0.0017**. Combined graph for *Ctrl* lines (*Ctrl 1–2, Iso-C9 4*) and *C9* (*C9 1–2–3–4*), Unpaired *t* test; *BiP*: *Ctrls* vs *Ctrls* + TU *t* = 8.187, *P* < 0.0001, ***; *C9* vs *C9* + TU *t* = 7.885, *P* < 0.0001***; *Ctrls* + TU vs *C9* + TU *t* = 2.995, *P* = 0.0057, **; *GRP75 *: *Ctrls* vs *Ctrls* + TU *t* = 11.75, *P* < 0.0001, ***; *C9* vs *C9* + TU *t* = 9.563, *P* < 0.0001,***; *Ctrls* + TU vs *C9* + TU *t* = 6.187, *P* < 0.0001***. **d** Representative immunofluorescence images showing BiP and GRP75 protein expression in 1- and 2-week-old iMNs for *C9ORF72* patient line 4 together with the corresponding isogenic control. Quantitative Analysis (Q.A.) of BiP and GRP75 expression reveals significantly increased levels of GRP75 and BiP in all *C9ORF72* lines when compared to controls or isogenic controls at 2-week post-differentiation, but not at 1 week. Unpaired *t* test; BiP: 2 weeks: *Ctrl(1)* vs *C9(1)* *t* = 12.24, *P* < 0.0001***; *Ctrl(2)* vs *C9(2)* *t* = 5.005, *P* < 0.0001***; *Ctrl(1)* vs *C9(3)* *t* = 13.41, *P* < 0.0001***; Iso-*C9(4)* vs *C9(4)* *t* = 10.02, *P* < 0.0001***; Iso-*C9(5)* vs *C9(5)* *t* = 7.109, *P* < 0.0001***. Combined graph for *Ctrl* lines (*Ctrl 1–2, Iso-C9 4–5*) and *C9* (*C9 1–2–3–4–5*), Unpaired *t* test; *BiP*: *Ctrls* 1 week vs *C9* 1 week *t* = 0.6497, *P* = 0.5167, n.s.; *Ctrls* 2 weeks vs *C9* 2 weeks *t* = 14.46, *P* < 0.0001, ***. Unpaired *t* test; GRP75 2 weeks: *Ctrl(1)* vs *C9(1)* *t* = 7.703, *P* < 0.0001***; *Ctrl(2)* vs *C9(2)* *t* = 11.08, *P* < 0.0001***; *Ctrl(1)* vs *C9(3)* *t* = 6.847, *P* < 0.0001***; Iso-*C9(4)* vs *C9(4)* *t* = 9.892, *P* < 0.0001***; Iso-*C9(5)* vs *C9(5)* *t* = 10.11, *P* < 0.0001***. Combined graph for *Ctrl* lines (*Ctrl 1–2, Iso-C9 4–5*) and *C9* (*C9 1–2–3–4–5*), Unpaired *t* test; *GRP75*: *Ctrls* 1 week vs *C9* 1 week *t* = 0.4153, *P* = 0.6788, n.s.; *Ctrls* 2 weeks vs *C9* 2 weeks *t* = 19.39, *P* < 0.0001, ***. **e** Representative western blot (WB) displaying increased expression of BiP and GRP75 in *C9(1–2)* and (4) compared to healthy *control(1–2)* or *Iso-C9(4)*. (Right) Q.A. of relative BiP and GRP75 expression, normalized to GAPDH; combined graph for *Ctrls(1–2–4)* and *C9(1–2–4)*. Unpaired *t* test; BIP: *Ctrl* vs *C9* *t* = 6.142, *P* < 0.0001***; Unpaired *t* test GRP75: *Ctrl* vs *C9* *t* = 6.536, *P* < 0.0001***. (*n* = 3 experiments from 3 different culture differentiations). Scale bar: **d** 5 μm

Neurons are selectively prone to ER stress in NDs, such as ALS and FTD, influencing disease manifestation and kinetics [25, 58]. The early phase of ER stress is accompanied by an increase in mitochondrial–ER contact sites and Ca^2+^ uptake, thus increasing ATP production [3, 25, 56]. We longitudinally evaluated ER stress responses by qPCR in five *C9ORF72*-ALS/FTD patient and four control iPSC-derived MN (iMN) cell lines, two of which were corresponding isogenic iMNs (see Supplementary Fig. 1a–d, online resource for phenotypic description, quality of differentiation and pathological hallmarks). Transcripts of the major luminal ER chaperone *BiP/GRP78* and its downstream effector *CHOP* were significantly higher in all 2-week-old *C9ORF72* iMNs; these levels increased further by 4 weeks, indicating growing impairment in ER homeostasis (Fig. 1a). We next examined whether the early phase of ER stress at 2 weeks modulates or affects MAM-associated molecules. Of the four types of connectors between the ER and mitochondria, the expression of *GRP75* transcript*,* involved in the tethering and Ca^2+^-signaling complex at MAMs, was consistently and significantly upregulated (two-to-fourfold higher) in all *C9ORF72* iMNs. Transcripts of both GRP75 binding partners, i.e., *ITPR3* and *VDAC1,* exhibited a significant trend toward higher expression (Fig. 1b). In contrast, other MAM connectors, *MFN1*, *MFN2*, *VAPB*, *RMDN3, FIS1,* and *BAP31* remained largely unchanged compared to the healthy or isogenic control iMNs (Supplementary Fig. 2a, online resource). Importantly, the two isogenic controls had reverted the observed *C9ORF72* phenotype involving ER stress and elevated *GRP75* expression (Fig. 1a, b).

To examine whether the increase in *GRP75* mRNA levels was a direct response to ER stress, we treated 1-week-old iMNs, which at this point lack ER stress, with tunicamycin (TU). To exclude cell death responses, which in cultured neurons are initiated at ~ 24 h and become significant around 48 h [54], we performed a mild treatment (TU: 1 µg/mL) for 18 h. TU treatment augmented *GRP75* mRNA levels in both control and mutant 1-week-old iMNs, suggesting that higher GRP75 transcripts reflected ongoing ER stress in iMNs (Fig. 1c). Of note, mutant iMNs exhibited a stronger response to TU treatment than healthy control iMNs. Moreover, other MAM molecules remained largely unaltered in response to the mild TU treatment: no significant alteration of twofold or more was observed (Supplementary Fig. 2b, online resource). We further validated GRP75 protein levels in iMNs at 1, 2, and 4 weeks by immunostaining. Corroborating the qPCR data, no change in BiP and GRP75 protein levels was observed in 1-week-old iMNs, whereas all *C9ORF72*-ALS/FTD patient lines exhibited higher expression of the ER stress marker BiP and GRP75 compared with healthy control iMNs and isogenic control lines after 2 weeks (Fig. 1d) and 4 weeks of differentiation (Supplementary Fig. 2c, online resource). As additional evidence, immunoblotting performed on 2-week-old iMNs confirmed elevated BiP and GRP75 expression in *C9ORF72-*ALS/FTD patient-derived iMNs (Fig. 1e). As shown in (Supplementary Fig. 1c, d, online resource), we detected no TDP-43 mislocalization or PolyGA aggregates in 2-week-old iMNs, suggesting that both ER stress and increased GRP75 levels are observed in iMNs before the appearance of these major *C9ORF72*-linked pathological hallmarks.

We next validated these observations using three *C9ORF72*-ALS/FTD patient-derived and three healthy control neuronal cell lines which were generated via direct conversion of fibroblasts to neurons (dNeus) [50], thereby preserving hallmarks of cellular aging (see Supplementary Fig. 3a, b, online resource for phenotypic description and quality of differentiation). Two-week-old *C9ORF72* dNeus showed increased levels of *BiP* and *CHOP* mRNA, and concomitantly higher protein levels of GRP75 was detected by immunostaining Supplementary Fig. 3c, d, online resource). Comparable to iMNs, dNeus also exhibited higher expression of *ITPR3* transcripts (Supplementary Fig. 3c, d, online resource), suggestive of a conserved ER stress response and associated changes in GRP75 expression in *C9ORF72*-ALS/FTD patient neurons.

### Enhanced ER–mitochondria association are specific to *C9ORF72* iMNs

**Fig. 2:**
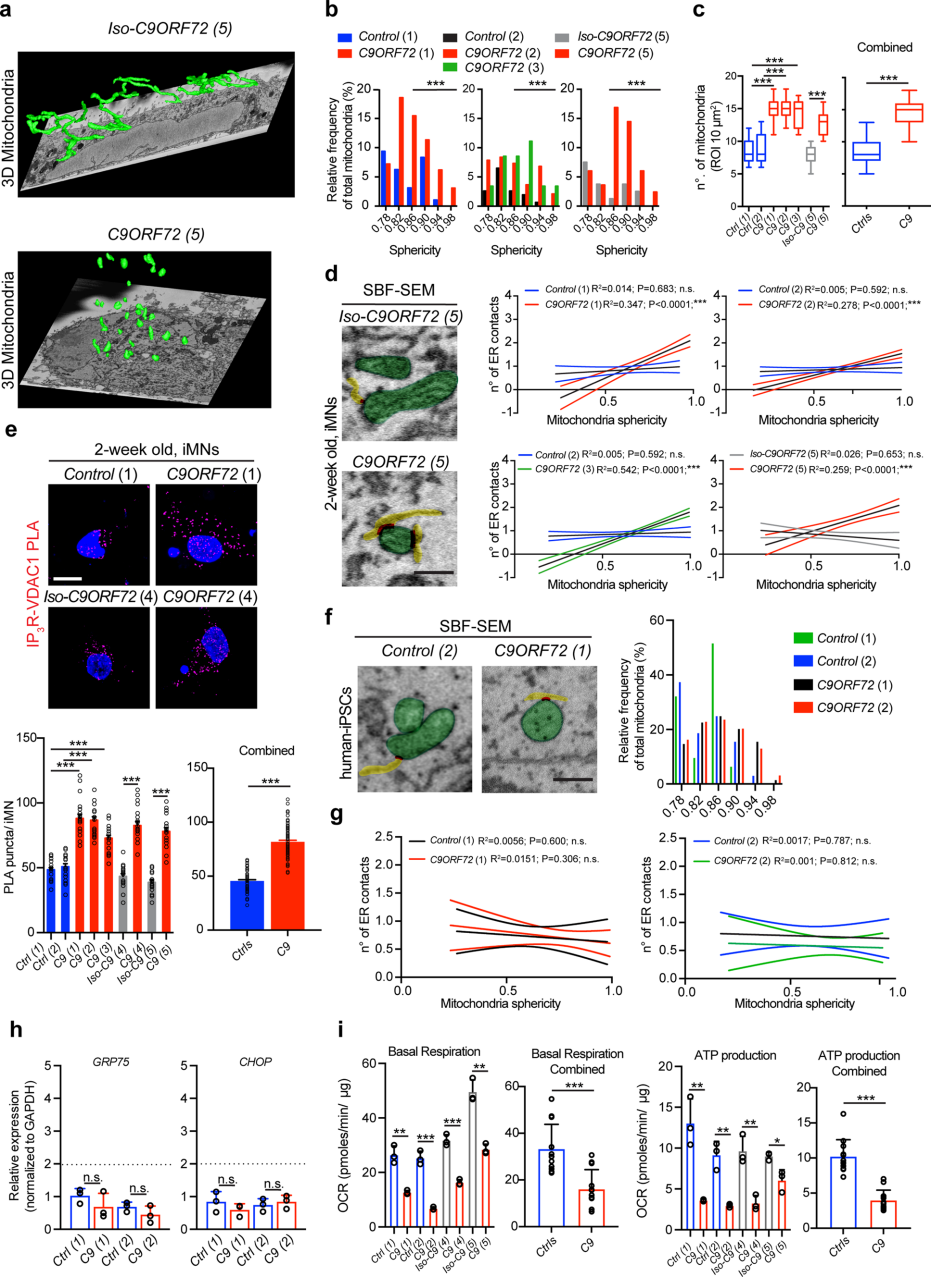
Mitochondrial alterations are present in *C9ORF72* iMNs and iPSCs. **a** Representative 3D segmentation of mitochondria morphology from SBF–SEM images in 2-week-old iMNs. The Isogenic line (5) displays tubular and elongated mitochondria, while the corresponding *C9ORF72* line displays small and rounded mitochondria. (no. of images: *Iso-C9ORF72(5)*: 285; *C9ORF72(5)*: 300). **b** Sphericity analysis performed on SBF–SEM image stacks, plotting sphericity values as relative frequency distribution histogram. Note the higher percentage of spherical mitochondria in mutant iMNs lines *C9ORF72(1), (2), (3)* and *(5)*; (*Ctrl(1)* bin center 0.86 = 3.125, 0.90 = 8.333, 0.94 = 1.0412, 0.98 = 0; *C9(1)* bin center 0.86 = 15.464, 0.90 = 11.34, 0.94 = 6.186, 0.98 = 3.093; Unpaired *t* test, mitochondria numbers *Ctrl(1)* *n* = 96 vs *C9ORF72(1)* *n* = 97, *t* = 4.833, *P* < 0.0001***; *Ctrl(2)* bin center 0.86 = 2.597, 0.90 = 1.948, 0.94 = 0.649, 0.98 = 0; *C9ORF72(2)* bin center 0.86 = 7.33, 0.90 = 3.665, 0.94 = 6.806, 0.98 = 2.094; *C9ORF72(3)* bin center 0.86 = 8.547, 0.90 = 11.111, 0.94 = 3.419, 0.98 = 3.419; Unpaired *t* test, mitochondria numbers *Ctrl(2)* *n* = 154 vs *C9ORF72(2*) *n* = 191, *t* = 4.369, *P* < 0.0001***; Unpaired *t* test, mitochondria numbers *Ctrl(2)* *n* = 154 vs *C9ORF72(3)* *n* = 117, *t* = 5.148, *P* < 0.0001***; *Iso-C9ORF72(5)* bin center 0.86 = 1.25, 0.90 = 3.75, 0.94 = 2.50, 0.98 = 0; *C9ORF72(5)* bin center 0.86 = 16.867, 0.90 = 14.458, 0.94 = 6.024, 0.98 = 2.410; Unpaired *t* test, mitochondria numbers *Iso-C9ORF72(5)* *n* = 80 vs *C9ORF72(5)* *n* = 83, *t* = 4.206, *P* < 0.0001***). **c** SBF–SEM Q.A. of mitochondria numbers in 2-week-old iMNs, showing increased number of mitochondria in all *C9ORF72* patient lines compared to controls or corresponding isogenic (Unpaired *t* test: *Ctrl(1)* vs *C9ORF72(1)* *t* = 7.556, *P* < 0.0001***; *Ctrl(2)* vs *C9ORF72(2)* *t* = 6.815, *P* < 0.0001***; *Ctrl(1)* vs *C9ORF72(3)*, *t* = 6.678, *P* < 0.0001***; *iso-C9ORF72(5)* vs *C9ORF72(5)*, *t* = 6.750, *P* < 0.0001***). Combined graph for *Ctrls* lines (*Ctrl 1–2, Iso-C9(5)*) and *C9* (*C9 1–2–3–5*), Unpaired *t* test *Ctrls* vs *C9* *t* = 12.53, *P* < 0.0001, ***. **d** Representative images of mitochondria contacts with ER for *Iso-C9ORF72(5)* and *C9ORF72(5)*. Linear regression between the no. of contacts per mitochondria and their sphericity in 2-week-old iMNs *(Ctrl(1);* *Y* = 0.4315*X + 0.5801, *P* = 0.2610, n.s.; *C9ORF72(1);* *Y* = 3.315*X − 1.161, *P* < 0.0001***; *Ctrl(2);* *Y* = 2.092*X + 0.7326, *P* = 0.3874, n.s.; *C9ORF72(2)*; *Y* = 1.856*X − 0.313, *P* < 0.0001 ***; *C9ORF72(4)* *Y* = 2.736*X − 0.9271, *P* < 0.0001***; *Iso-C9ORF72(6)*: *Y* =  − 0.5363*X + 1.129, *P* = 0.1530, n.s.; *C9ORF72(6)*:*Y* = 2.226*X − 0.1446, *P* < 0.0001 ***). **e** Representative images depicting increased number of PLA puncta between IP_3_R and VDAC1 in 2-week-old *C9ORF72(1)* iMNs compared to *Ctrl(1)* or *C9ORF72(5)* compared to the respective isogenic control. PLA IP_3_R–VDAC1: Unpaired *t* test: *Ctrl(1),* *n* = 21 vs *C9ORF72(1),* *n* = 21, *t* = 11.01, *P* < 0.0001***; *Ctrl(2),* *n* = 19 vs *C9ORF72(2),* *n* = 22, *t* = 9.979, *P* < 0.0001*** *Ctrl(1),* *n* = 21 vs *C9ORF72(3),* *n* = 20, *t* = 8.637, *P* < 0.0001***; *Iso-C9ORF72(4),* *n* = 19 vs *C9ORF72(4),* *n* = 21, *t* = 10.55, *P* < 0.0001***; *Iso-C9ORF72(5),* *n* = 17 vs *C9ORF72(5),* *n* = 18, *t* = 11.08, *P* < 0.0001***, *n* = cell numbers. Combined graph for *Ctrl* lines (*Ctrl 1–2, Iso-C9 4–5*) and *C9* (*C9 1–2–3–4–5*), Unpaired *t* test *Ctrls* vs *C9* *t* = 19.60, *P* < 0.0001, ***. **f** Sphericity analysis performed on SBF–SEM image stacks of iPSCs from *Ctrl 1 and 2* and *C9 1 and 2,* plotting sphericity values as relative frequency distribution histogram. Note the higher percentage of spherical mitochondria in mutant iPSCs lines *C9ORF72(1) and (2)*. **g** Linear regression between the no. of contacts per mitochondria and their sphericity in iPSCs *(Ctrl(1);* *Y* =  − 0.2617*X + 0.8871, *P* = 0.6003, n.s.; *C9ORF72(1);* *Y* =  − 0.4234*X + 1.024, *P* = 0.3064, n.s.; *Ctrl(2);* *Y* =  − 0.1084*X + 0.8194, *P* = 0.7869, n.s.; *C9ORF72(2);* *Y* =  − 0.1102*X + 0.6515, *P* = 0.8117, n.s.). **h** qPCR analysis for *GRP75* and *CHOP* transcripts from *Ctrls(1–2)* and *C9ORF72(1–2)* iPSCs lines, show no changes between *Ctrls* and *C9ORF72* iPSCs. **i** Mitochondria oxygen consumption rate (OCR) analysis on iPSCs reveals impairments in basal respiration and ATP production in all *C9ORF72* patients’ lines compared to *Ctrls* or respective isogenic. Unpaired *t* test basal respiration*: Ctrl(1)* vs *C9(1)* *t* = 7.102, *P* = 0.0021**; *Ctrl(2)* vs *C9(2)* *t* = 12.45, *P* = 0.0002***; Iso-*C9(4)* vs *C9(4)* *t* = 11.41, *P* = 0.0003***; *Iso-C9(5)* vs *C9(5)* *t* = 7.677, *P* = 0.0015**; ATP production*: Ctrl(1)* vs *C9(1)* *t* = 5.473, *P* = 0.0054**; *Ctrl(2)* vs *C9(2)* *t* = 6.401, *P* = 0.0031**; Iso-*C9(4)* vs *C9(4)* *t* = 5.488, *P* = 0.0054**; *Iso-C9(5)* vs *C9(5)* *t* = 3.708, *P* = 0.0207*; Combined graph for *Ctrl* lines (*Ctrl 1–2, Iso-C9 4–5*) and *C9* (*C9 1–2–4–5*), Unpaired *t* test, basal respiration *Ctrls* vs *C9* *t* = 4.438, *P* = 0.0002, ***; ATP production *Ctrls* vs *C9* *t* = 7.741, *P* < 0.0001, ***. Scale bar: **d** and **f** 0.5 μm, **e** 10 μm

GRP75 serves as a functional linker protein between the ER and the mitochondria. Thus, any changes in its expression pattern would influence both cellular compartments. Therefore, we examined whether mitochondria in *C9ORF72* iMNs display early structural or functional alterations. To this end, we performed serial block-face scanning electron microscopy (SBF–SEM) on four *C9ORF72*, two healthy and one isogenic control iMNs. 3D reconstruction of images revealed that in all four *C9ORF72* iMNs, other than in the healthy or isogenic controls, a large fraction of mitochondria were rounded in shape (Fig. 2a). To obtain a quantitative measure for the observed mitochondrial shape changes, we measured mitochondrial sphericity and plotted the values as a frequency distribution [61]. Applying a cutoff from 0.86 to 0.98, where values closer to 1.0 indicate a perfectly spherical object, all four *C9ORF72* iMNs presented a significantly higher proportion of rounded mitochondria compared to healthy/isogenic controls (Fig. 2b). Subsequently, we assessed mitochondrial numbers, which were also on average higher in all four *C9ORF72* iMNs compared to the control iMNs (Fig. 2c). Notably, the isogenic control *Iso-C9ORF72(5)* again had reverted the mitochondrial phenotype displayed by the mutant *C9ORF72(5)* line. Measuring the number of ER contacts that each mitochondrion made revealed that spherical mitochondria in mutant MNs on average exhibited a higher number of contact points (Fig. 2d). As GRP75 physically tethers IP_3_R–VDAC1, thereby promoting mitochondrial Ca^2+^ uptake, we quantified the IP_3_R–VDAC1 interaction via proximity ligation assay (PLA). All *C9ORF72* iMNs at 2 weeks presented increased interaction between the two partners (Fig. 2e).

We next assessed whether the changes in mitochondrial structure were present within *C9ORF72* iPSCs and might reflect mutation-related alterations. SBF–SEM was performed on two *C9ORF72* and two healthy control iPSCs. Both *C9ORF72* iPSCs displayed higher proportion of spherical mitochondria compared to controls (Fig. 2f). Notably, the number of ER–mitochondria contacts remained unchanged between mutant and control-iPSCs (Fig. 2g). Further, measurement of *GRP75* and *CHOP* transcripts revealed no change in expression levels, suggestive of those expression changes being specific to iMNs (Fig. 2h). We evaluated key parameters of mitochondrial function using the Seahorse assay and found reduced basal respiration and consequently lower ATP production in *C9ORF72* iPSCs (Fig. 2i). Taken together, these data indicate that intrinsic mitochondrial deficits are present in *C9ORF72* cells and that specifically *C9ORF72* iMNs undergo early increase in ER–mitochondria associations in parallel to the observed ER stress.

### Increased ER–mitochondria association via GRP75 normalizes Ca^2+^ uptake and ATP generation in *C9ORF72* iMNs

**Fig. 3:**
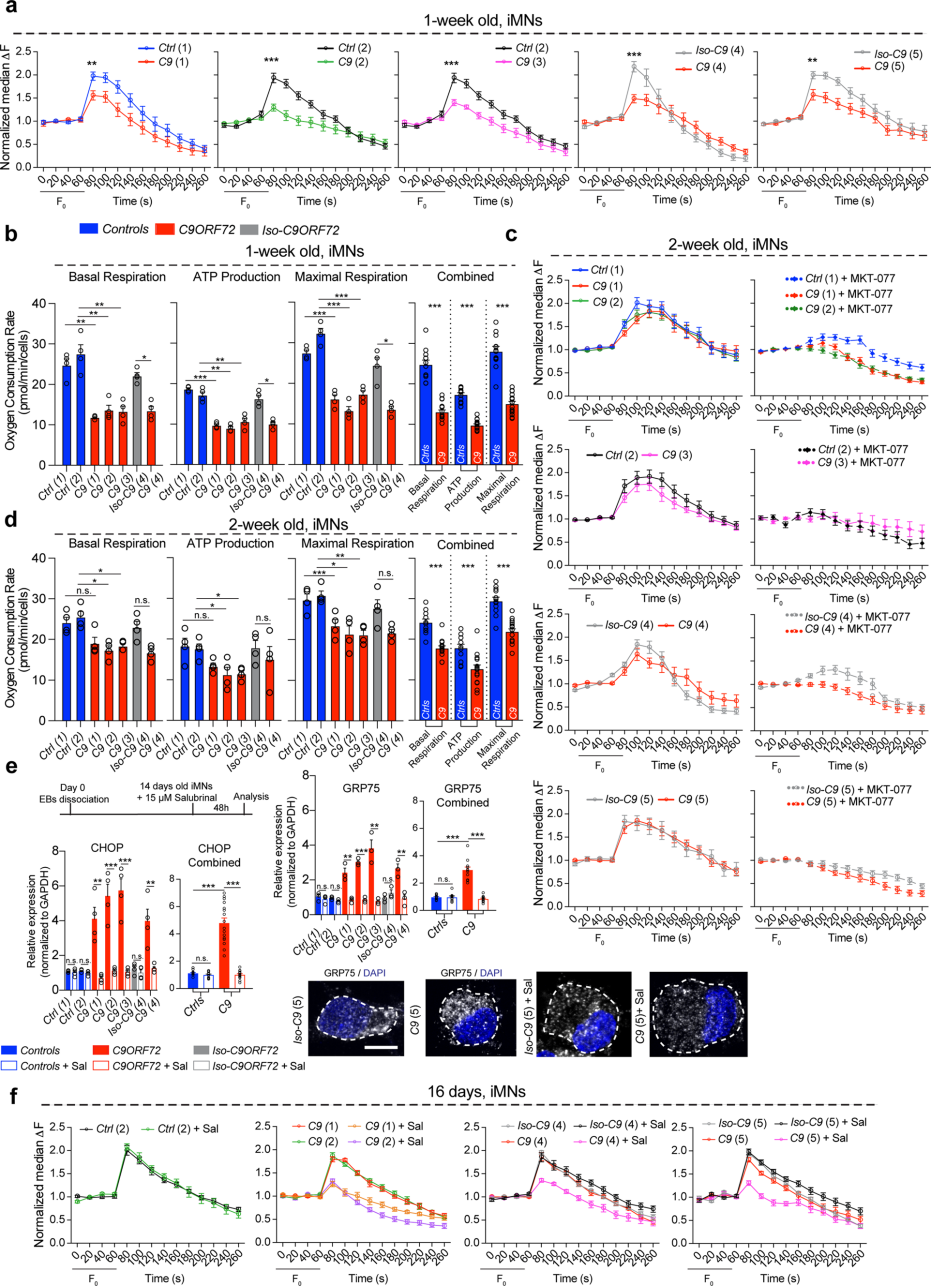
Enhanced ER–mitochondria coupling via GRP75 promotes optimal mitochondrial calcium uptake and bioenergetics. **a** Baseline (0–60 s) and stimulated mitochondrial calcium (Ca^2+^) uptake (80 s onward) traces from *Ctrls(1 and 2)* and *C9ORF72(1,2,3)* iMNs and *Iso-C9ORF72(4 and 5)* and *C9ORF72(4 and 5)* iMNs at 1-week post-differentiation when there is no ER stress or GRP75 upregulation. All patient lines exhibited decreased mitochondrial Ca^2+^ transients compared to controls or respective isogenic controls. (No. of iMNs: *Ctrl(1)*: 38, *Ctrl(2)*: 22, *C9(1)* 47, *C9(2)* 20; *C9(3)*: 22, *Iso-C9(4)*: 40, *C9(4)*: 38, *Iso-C9(5)*: 19, *C9(5)*: 20, from 3–4 independent cultures). Multiple *t* test at 80 s: *Ctrl(1)* mean = 1.97, *C9(1)* mean = 1.56, *P* = 0.0046; *Ctrl(2)* mean = 1.94, *C9(2)* mean = 1.30, *P* < 0.0001; *Ctrl(2)* mean = 1.94, *C9(3)* mean = 1.40, *P* = 0.0002; *Iso-C9(4)* mean = 2.18, *C9(4)* mean = 1.49, *P* < 0.0001, *iso-C9(5)* mean = 2.00, *C9(5)* mean = 1.57, *P* = 0.004). **b** Mitochondria oxygen consumption rate analysis (Seahorse) on 1-week-old iMNs reveals impairments in basal respiration, ATP production and maximal respiration in all *C9ORF72* patients lines compared to control or respective isogenic lines. (Paired *t* test: basal respiration*: Ctrl(1)* vs *C9(1)* *t* = 7.756, *P* = 0.0045**; *Ctrl(2)* vs *C9(2)* *t* = 11.33, *P* = 0.0015**; *Ctrl(2)* vs *C9(3)* *t* = 7.043, *P* = 0.0059**; *Iso-C9(4)* vs *C9(4)* *t* = 4.99, *P* = 0.0155*; ATP production*: Ctrl(1)* vs *C9(1)* *t* = 26.13, *P* = 0.0001***; *Ctrl(2)* vs *C9(2)* *t* = 6.916, *P* = 0.0062**; *Ctrl(2)* vs *C9(3)* *t* = 6.924, *P* = 0.0062**; *Iso-C9(4)* vs *C9(4)* *t* = 4.432, *P* = 0.0213*; maximal respiration*: Ctrl(1)* vs *C9(1)* *t* = 30.19, *P* < 0.0001***; *Ctrl(2)* vs *C9(2)* *t* = 18.27, *P* = 0.0004***; *Ctrl(2)* vs *C9(3)* *t* = 14.10, *P* = 0.0008***; *Iso-C9(4)* vs *C9(4)* *t* = 4.544, *P* = 0.020*). Combined graph for *Ctrl* lines (*Ctrl 1–2, Iso-C9 4*) and *C9* (*C9 1–2–3–4*), Unpaired *t* test basal respiration *Ctrls* vs *C9* *t* = 10.47, *P* < 0.0001, ***; ATP production *Ctrls* vs *C9* *t* = 13.79, *P* < 0.0001, ***; maximal respiration *Ctrls* vs *C9* *t* = 10.26, *P* < 0.0001, ***. **c** Baseline (0–60 s (s)) and stimulated mitochondrial Ca^2+^ uptake (80 s onward) traces from *Ctrls(1 and 2)* and *C9(1,2,3)* iMNs and *Iso-C9(4 and 5)* and *C9(4 and 5)* iMNs at 2-week post-differentiation. All the patients’ lines show comparable mitochondria Ca^2+^ transient to controls or respective isogenic controls. (Number of iMNs: *Ctrl(1)*: 38, *Ctrl(2)*: 22, *C9(1)* 47, *C9(2)* 20; *C9(3)*: 22, *Iso-C9(4)*: 40, *C9(4)*: 38, *Iso-C9(5)*: 19, *C9(5)*: 20, from 3–4 independent cultures). Multiple *t* test at 120 s: *Ctrl(1)* mean = 1.93, *C9(1)* mean = 1.83, *P* = 0.577; *Ctrl(1)* mean = 1.93, *C9(2)* mean = 1.81, *P* = 0.198; *Ctrl(2)* mean = 1.91, *C9(3)* mean = 1.75, *P* = 0.416; Multiple *t* test at 100 s: *Iso-C9(4)* mean = 1.84, *C9(4)* mean = 1.63, *P* = 0.218, *iso-C9(5)* mean = 1.80, *C9(5)* mean = 1.86, *P* = 0.776). (Right) Treatment with GRP75 inhibitor MKT-077 completely abolished Ca^2+^ transients in *C9ORF72* patient iMNs. Control and Isogenic lines also reveal reduction in Ca^2+^ transients after MKT-077 treatment. MKT-077 curves are plotted as dotted lines (Number of iMNs*: Ctrl(1)* + *MKT-077*: 39, *Ctrl(2)* + *MKT-077*: 13, *C9(1)* + *MKT-077:* 40, *C9(2)* + *MKT-077:* 21; *C9(3)* + *MKT-077*: 11, *Iso-C9(4)* + *MKT-077*: 39, *C9(4)* + *MKT-077*: 39, *Iso-C9(5)* + *MKT-077*: 21, *C9(5)* + *MKT-077*: 21, from 5–6 independent cultures). Multiple *t* test at 100 s: *Ctrl(1)* mean = 2.008, *Ctrl(1)* + *MKT-077* mean = 1.26, *P* < 0.0001. *Ctrl(2)* mean = 1.89, *Ctrl(2)* + *MKT-077* mean = 1.104, *P* = 0.0003; *C9(1)* mean = 1.64, *C9(1)* + *MKT-077,* mean = 1.14, *P* < 0.0001; *C9(2)* mean = 1.75, *C9(2)* + *MKT-077* mean = 0.98, *P* < 0.0001; *C9(3),* mean = 1.74, *C9(3)* + *MKT-077,* mean = 1.07, *P* = 0.005; *Iso-C9(4),* mean = 1.84, *Iso-C9(4)* + *MKT-077,* mean = 1.30, *P* = 0.0002; *C9(4),* mean = 1.63, *C9(4)* + *MKT-077,* mean = 0.999, *P* < 0.0001; *Iso-C9(6),* mean = 1.80, *Iso-C9(6)* + *MKT-077* mean = 0.93, *P* < 0.0001; *C9(6),* mean = 1.86, *C9(6)* + *MKT-077,*mean = 0.83, *P* < 0.0001). **d** Mitochondria oxygen consumption rate analysis (OCR) via Seahorse assay in 2-week-old iMNs. When GRP75 is upregulated patient lines show improvement in basal respiration, ATP production and maximal respiration compared to the 1-week condition. (Paired *t* test basal respiration*: Ctrl(1)* vs *C9(1)* *t* = 2.064, *P* = 1309 n.s.; *Ctrl(2)* vs *C9(2)* *t* = 5.624, *P* = 0.0111*; *Ctrl(2)* vs *C9(3)* *t* = 4.417, *P* = 0.0215*; *Iso-C9(4)* vs *C9(4)* *t* = 2.741, *P* = 0.0713 n.s.; ATP production*: Ctrl(1)* vs *C9(1)* *t* = 2.138, *P* = 0.122 n.s.; *Ctrl(2)* vs *C9(2)* *t* = 4.371, *P* = 0.0222*; *Ctrl(2)* vs *C9(3)* *t* = 4.183, *P* = 0.0249*; *Iso-C9(4)* vs *C9(4)* *t* = 0.9993, *P* = 0.3913 n.s.; maximal respiration*: Ctrl(1)* vs *C9(1)* *t* = 14.82, *P* = 0.0007***; *Ctrl(2)* vs *C9(2)* *t* = 3.547, *P* = 0.0382*; *Ctrl(2)* vs *C9(3)* *t* = 8.631, *P* = 0.0033**; *Iso-C9(4)* vs *C9(4)* *t* = 2.982, *P* = 0.0585 n.s.). Combined graph for *Ctrl* lines (*Ctrl 1–2, Iso-C9 4*) and *C9* (*C9 1–2–3–4*), Unpaired *t* test basal respiration *Ctrls* vs *C9* *t* = 6.807, *P* < 0.0001***; ATP production *Ctrls* vs *C9* *t* = 3.865, *P* = 0.0007***; maximal respiration *Ctrls* vs *C9* *t* = 6.613, *P* < 0.0001***. **e** qPCR analysis of *CHOP* and *GRP75* transcripts after 48 h of 15 μM Salubrinal treatment. Salubrinal treatment suppress ER stress and reduce *GRP75* transcript levels to controls in *C9ORF72* patients lines but no change was detected in control or isogenic control lines. Unpaired *t* test *CHOP*: *Ctrl(1)* vs *Ctrl(1)* + Sal *t* = 0.379, *P* = 0.7177 n.s.; *Ctrl(2)* vs *Ctrl(2)* + Sal *t* = 1.289, *P* = 0.2448 n.s.; *C9(1)* vs *C9(1)* + Sal *t* = 4.975, *P* = 0.0025**; *C9(2)* vs *C9(2)* + Sal *t* = 6.161, *P* = 0.0008***; *C9(3)* vs *C9(3)* + Sal *t* = 6.702, *P* = 0.0005***; *Iso-C9(4)* vs *Iso-C9(4)* + Sal *t* = 1.004, *P* = 0.3542 n.s.; *C9(4)* vs *C9(4)* + Sal *t* = 3.876, *P* = 0.0082**; Combined graph for control lines (*Ctrl 1–2, Iso-C9 4*) and *C9* (*C9 1–2–3–4*): unpaired *t* test *CHOP*: *Ctrls* vs *C9* *t* = 8.571, *P* < 0.0001***; *Ctrls* vs *Ctrls* + Sal *t* = 1.507, *P* = 0.1459, n.s.; *C9* vs *C9* + Sal *t* = 10.10, *P* < 0.0001, ***. Unpaired *t* test *GRP75*: *Ctrl(1)* vs *Ctrl(1)* + Sal *t* = 0.7898, *P* = 0.4738 n.s.; *Ctrl(2)* vs *Ctrl(2)* + Sal *t* = 2.356, *P* = 0.078 n.s.; *C9(1)* vs *C9(1)* + Sal *t* = 5.251, *P* = 0.0063**; *C9(2)* vs *C9(2)* + Sal *t* = 14.76, *P* = 0.0001***; *C9(3)* vs *C9(3)* + Sal *t* = 5.881, *P* = 0.00042***; *Iso-C9(4)* vs *Iso-C9(4)* + Sal *t* = 1.289, *P* = 0.2670 n.s.; *C9(4)* vs *C9(4)* + Sal *t* = 4.754, *P* = 0.0089***). Combined graph for *Ctrl* lines (*Ctrl 1–2, Iso-C9 4*) and *C9*(*C9 1–2–3–4*): unpaired *t* test *GRP75*: *Ctrls* vs *C9* *t* = 7.922, *P* < 0.0001***; *Ctrls* vs *Ctrls* + Sal *t* = 0.111, *P* = 0.9130, n.s.; *C9* vs *C9* + Sal *t* = 9.617, *P* < 0.0001, ***. qPCR graphs plotted with s.d., *n* = 3–4 independent qPCR experiments repeated in triplicate. **f** Baseline (0–60 s) and stimulated mitochondrial Ca^2+^ uptake (80 s onward), traces from *Ctrl(2)* and *C9ORF72(1,2,3)* iMNs and *Iso-C9ORF72(4 and 5)* and *C9ORF72(4 and 5)* iMNs after Salubrinal treatment are shown. All patient lines show reduced mitochondria Ca^2+^ transients compared to controls or respective isogenic controls. (Number of iMNs: *Ctrl(2)*: 10, *Ctrl(2)* + Sal: 13, *C9(1):* 10, *C9(1)* + Sal: 12, *C9(2):* 10, *C9(2)* + Sal: 13, *Iso-C9(4)*: 10, *Iso-C9(4)* + Sal: 11, *C9(4)*: 10, *C9(4)* + Sal: 12, *Iso-C9(5)*: 10, *Iso-C9(5)* + Sal: 11, *C9(5)*: 11, *C9(5)* + Sal: 12). Multiple *t* test at 80 s: *Ctrl(2)* mean: 2.00, *Ctrl(2)* + Sal mean 2.08, *P* = 0.55; *C9(1)* mean: 1.79, *C9(1)* + Sal mean:1.26, *P* < 0.0001, *C9(2)* mean: 1.87, *C9(2)* + Sal mean:1.33, *P* < 0.0001; *Iso-C9(4)* mean: 1.96, *Iso-C9(4)* + Sal mean: 1.85, *P* = 0.354, *C9(4)* mean: 1.87, *C9(4)* + Sal mean: 1.35, *P* < 0.0001, *Iso-C9(5)* mean: 1.94, *Iso-C9(5)* + Sal mean: 1.98, *P* = 0.632; *C9(5)* mean: 1.81, *C9(5)* + Sal mean: 1.30, *P* < 0.0001. Scale bar: **e** 10 μm

GRP75 creates a physical link between the ER membrane and the outer mitochondrial membrane by facilitating the interaction between ER-bound IP_3_Rs and mitochondrial VDAC1 to promote core mitochondrial Ca^2+^ uptake and optimal mitochondrial bioenergetics [62]. Dysregulation of mitochondrial Ca^2+^ homeostasis and mitochondrial Ca^2+^ overload has been linked to neuronal death in neurodegenerative disorders [10, 59, 60]. Therefore, we measured mitochondrial Ca^2+^ uptake in *C9ORF72* and control iMNs after 1 and 2 weeks of differentiation. Fluo-4AM was combined with an intracellular buffer that eliminated cytosolic and ER Ca^2+^ signals [47], thereby enabling specifically the measurement of mitochondrial Ca^2+^ uptake. Unexpectedly, all 1-week-old *C9ORF72* iMNs exhibited significant mitochondrial Ca^2+^ uptake deficits when compared with healthy or isogenic control iMNs, even though iMNs at 1 week do not yet manifest ER stress or GRP75 expression changes (Fig. 3a). Not only were Ca^2+^ transients lower within the mitochondria, but also the mitochondrial membrane potential, which is the driving force for Ca^2+^ uptake, was significantly reduced when measured simultaneously using ΔΨM probe tetramethylrhodamine methyl ester (TMRM) dye (Supplementary Fig. 4a, b, online resource). To follow up on this, we evaluated key parameters of mitochondrial function using the Seahorse assay and found reduced basal and maximal respiration, and consequently lower ATP production, suggesting that mitochondrial Ca^2+^ uptake deficits observed in 1-week-old *C9ORF72* iMNs likely precede the ER stress response (Fig. 3b).

We next measured mitochondrial Ca^2+^ uptake in 2-week-old *C9ORF72* iMNs, which exhibit ER stress and increased GRP75 expression. Upon Ca^2+^ release from the ER, the maximum mitochondrial Ca^2+^ uptake and mitochondrial membrane potentials were not significantly altered in these neurons compared with their healthy/isogenic controls (Fig. 3c and Supplementary Fig. 4c, online resource). The mitochondrial Ca^2+^ transients thus detected are a cumulative readout of the release of Ca^2+^ from ER stores not only by IP_3_R, but also via ryanodine receptors and sphingolipid Ca^2+^ release-mediating proteins of the ER. Therefore, to reveal the contribution of increased GRP75 to mitochondrial Ca^2+^ uptake, iMNs were treated with MKT-077, an established pharmacological inhibitor of GRP75 [26], at different concentrations to establish an optimum dosage curve for the inhibition of mitochondrial Ca^2+^ uptake specifically in iMNs (Supplementary Fig. 4d, online resource). 5 µM MKT-077 treatment led to reduced mitochondrial Ca^2+^ transients within both healthy and isogenic controls. However, in all *C9ORF72* iMNs mitochondrial Ca^2+^ transients were significantly impaired (Fig. 3c), suggesting that elevated GRP75 levels likely promoted optimal ER–mitochondrial association, thereby reversing mitochondrial Ca^2+^ uptake deficits in *C9ORF72* iMNs. Notably, the ameliorated mitochondrial Ca^2+^ uptake within 2-week-old iMNs was also reflected by almost normalized mitochondrial respiration and ATP production in *C9ORF72* iMNs (Fig. 3d and Supplementary Fig. 4e, online resource).

As further evidence for the involvement of ER stress-mediated GRP75 increase in normalizing mitochondrial Ca^2+^ uptake, we treated 2-week-old iMNs harboring higher GRP75 levels with 15 μM ER stress inhibitor salubrinal (Sal) for 48 h. This abrogated ER stress and normalized GRP75 expression in *C9ORF72* iMNs (Fig. 3e). Sal treatment led to reduced mitochondrial Ca^2+^ transients within *C9ORF72* iMNs, but had no effect on control/isogenic control iMNs (Fig. 3f). Our data suggest that *C9ORF72* iMNs exhibit early mitochondrial impairments, which are neutralized by ER stress-mediated elevated GRP75 expression, suggestive of an early adaptive response crucial for sustaining mitochondrial function.

### Reduced GRP75 levels in *C9ORF72*-ALS/FTD post-mortem tissue and *C9-500* rodent neurons

**Fig. 4:**
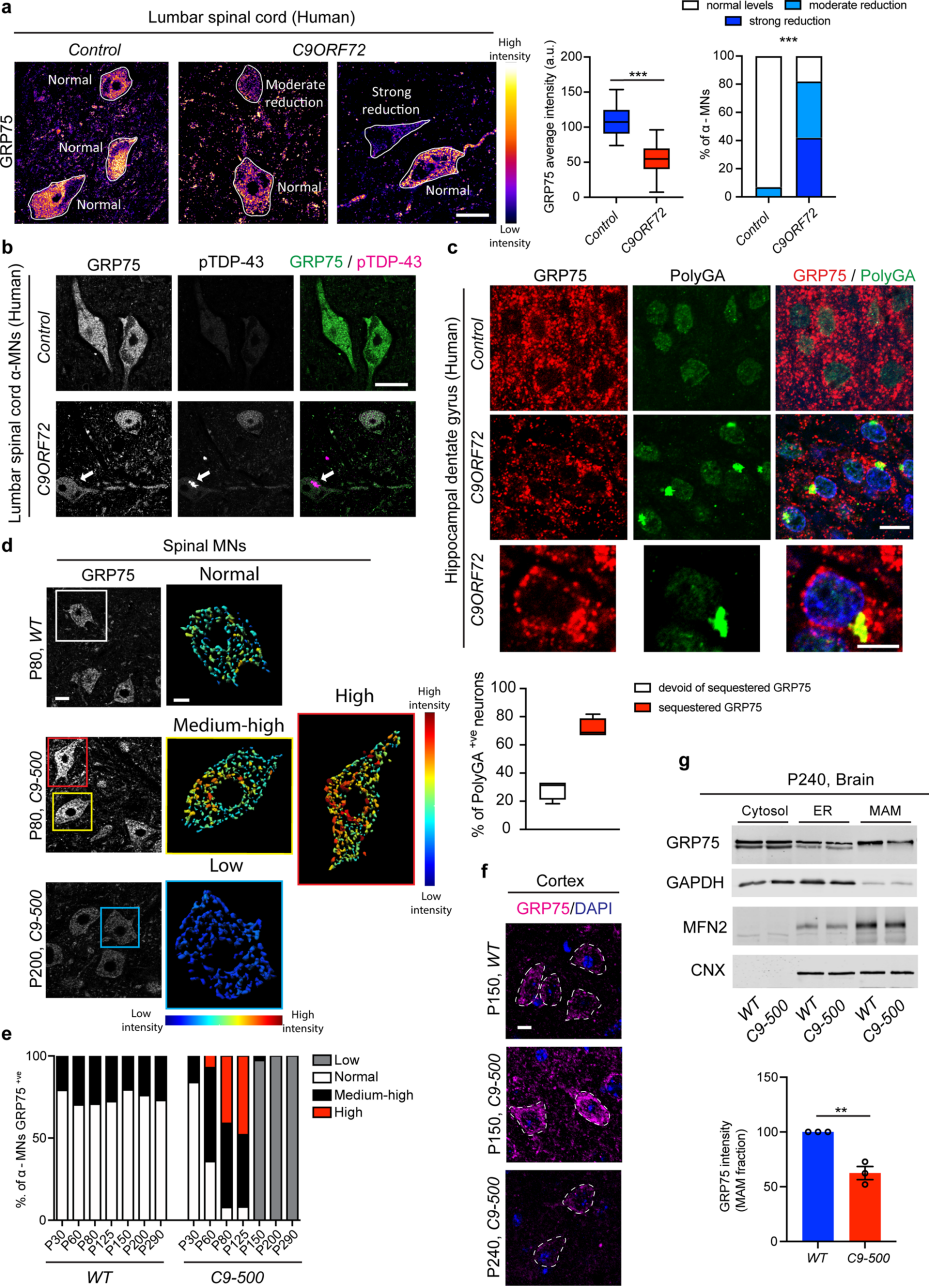
Age-dependent reduction in GRP75 expression in human and rodent *C9ORF72* CNS tissue. **a** Representative images showing differential GRP75 immunoreactivity in human lumbar spinal cord anterior horns of *C9ORF72* ALS patients compared to the normal controls. The variable immunofluorescence intensity was further scored as low, normal, medium and high as depicted. Images are shown from two *C9ORF72* patients and rendered for expression intensity. Right: Q.A. of GRP75 average fluorescence intensity: unpaired *t* test *Control* vs *C9ORF72*, *t* = 11.61, *P* < 0.0001***; Q.A. of GRP75 immunoreactivity: normal (*C9ORF72* 18%), moderate reduction (*C9ORF72* 40%), strong reduction (*C9ORF72* 42%), Chi-square analysis *P* < 0.0001***. (Number of MNs analyzed, *Control*: 29; *C9ORF72*: 77), *n* = 4 *C9ORF72*-fALS patients and *n* = 4 age-matched controls. Note that in *Control* α-MNs, GRP75 immunoreactivity was uniform, whereas more α-MNs from *C9ORF72*-ALS/FTD cases were examined in detail to account for the high degree of variability in GRP75 staining. **b** Representative double immunofluorescence labeling of human *C9ORF72*-ALS/FTD α-MNs within the lumbar spinal cord using antibodies against GRP75 and pTDP-43. Arrow: pTDP-43-positive inclusion. **c** Representative double immunofluorescence labeling of *C9ORF72*-ALS/FTD patient hippocampal dentate gyrus neurons compared to normal control. Note the overall reduced labeling of GRP75 and its focal sequestration with PolyGA aggregates in *C9ORF72-*ALS/FTD patient dentate gyrus hippocampal neurons, where 71.50% ± 3.6 of the neurons showed GRP75 sequestration within PolyGA aggregates. Sixty PolyGA aggregate bearing neurons were analyzed. **d** Representative confocal images of spinal MNs stained for GRP75 from *WT* and *C9-500* animals at different ages. 3D rendering of MNs based on the intensity level of GRP75 staining show increased levels of GRP75 expression at P80 and decreased levels of GRP75 expression in P200 *C9-500* animals compared to *WT*. **e** Longitudinal Q.A. of GRP75 expression in *WT* and *C9-500* animals showing MNs with high GRP75 levels at P80 and P120 (41–48%) with subsequent drop in expression after P150, where GRP75 levels are below WT expression levels. **f** Representative confocal immunofluorescence imaging of GRP75 expression in *WT* and *C9-500* cortex showing increased GRP75 levels at P150 and below *WT* levels of expression at P240. **g** Representative WB of brain extracts from *WT* and *C9-500* animals at P240. MAM isolation using sucrose gradient revealed decreased GRP75 levels in *C9-500* animals compared to *WT*, while no difference was detected for the loading controls MFN2. Quantitative analysis of the intensity of GRP75 at the MAM compared to *WT* (Unpaired *t* test: *t* = 6.263, *P* = 0.0033**). *N* = 3 mice, repeated thrice as separate experiments. Scale bars: **a** 15 µm, **b** 60 µm, **c** 8 µm, **d** 30 and 10 µm (zoom), **f** 30 µm

We next examined lumbar spinal cord specimens of four *C9ORF72-*ALS/FTD and four control cases by immunofluorescence and DAB immunohistochemistry. We found homogeneous, strong cytoplasmic GRP75 immunoreactivity of numerous large and small anterior horn neurons in the control cases. In contrast, a large fraction of the remaining neurons in *C9ORF72*-ALS/FTD cases showed a considerable reduction in average GRP75 immunoreactivity (Fig. 4a and Supplementary Fig. 5a, online resource). Many α-MNs in the lumbar spinal cord of the *C9ORF72*-ALS/FTD patients contained characteristic pTDP-43 aggregates of varying morphology (dash- or dot-like/granular, skein-like, dense/globular, Supplementary Fig. 5b, online resource), probably depending upon their stage of maturation [52]. We consistently observed that α-MNs harboring large, compact globular or skein-like pTDP-43 aggregates showed reduced cytoplasmic GRP75 immunoreactivity in comparison to the adjacent α-MNs with high levels of GRP75 that were either completely devoid of pTDP-43 aggregates or harbored only minor amounts of small, dispersed, granular pTDP-43 microaggregates. Of note, we also observed rare α-MNs, in which intense GRP75 immunoreactivity coincided with larger amounts of mostly granular pTDP-43 (Fig. 4b, and Supplementary Fig. 5c, online resource, bottom image).

Similarly, an overall reduction in cytoplasmic GRP75 immunoreactivity of hippocampal dentate gyrus neurons in the same *C9ORF72*-ALS/FTD patients as studied above was observed (Supplementary Fig. 5d, online resource). Notably, this was prominent in nearly all pTDP-43 aggregate-bearing dentate gyrus neurons (Supplementary Fig. 5d, online resource, red arrows). In line with this observation, PolyGA aggregate-bearing dentate gyrus neurons generally showed reduced GRP75 immunoreactivity in comparison to the adjacent neurons devoid of PolyGA aggregates (Fig. 4c). On the other hand, in those neurons which harbored PolyGA aggregates, the remaining GRP75 immunoreactivity often showed a tendency to preferentially co-localize with PolyGA accumulations (Fig. 4c).

Reprogramming resets the epigenetic age of induced pluripotent stem cells (iPSCs), [49]; therefore, we assume that iPSC-derived neurons used in this study most closely resemble those in “young” mutation carriers. This renders them suitable tools for identifying the molecular mechanism by which “young” MNs might be still able to protect themselves against abnormal ALS proteins. Thus, for the assessment of GRP75 expression relevant to the chronic course and later stages of the disease, we examined GRP75 levels in the *C9-500* BAC mouse model. These mice display RNA foci, pTDP43 aggregates and robust accumulation of DPRs, all of which are pathological hallmarks of *C9ORF72*-ALS/FTD [9, 41]. We first found an early transient increase in GRP75 immunoreactivity in *C9-500* spinal cord MNs compared to *WT*. Notably, this increase was elevated within MN pools, but variable levels of GRP75 expression were observed (Fig. 4d). Interestingly, this increase in GRP75 expression (classified as medium–high to high) was transient from postnatal (P) days 60–125 and abruptly reduced to below *WT* levels from P150 to beyond 200 days (Fig. 4e). Within the cortex, we found a similar temporary increase in GRP75 expression, albeit slightly shifted in time compared with spinal MNs (Fig. 4f), thus corroborating both our previous observations made in iMNs and post-mortem tissue. Next, we investigated whether the overall decline in GRP75 expression was also reflected by its specific reduction at the MAM. To this end, we isolated MAM fractions from the brain of P240 *C9-500* mice. Immunoblotting MAM fractions confirmed lower levels of GRP75 localized at the MAM, indicating that this reduction might negatively affect mitochondrial Ca^2+^ uptake and downstream function (Fig. 4g).

### Loss of GRP75 expression and mitochondrial dysfunction coincides with the onset of UPR

**Fig. 5:**
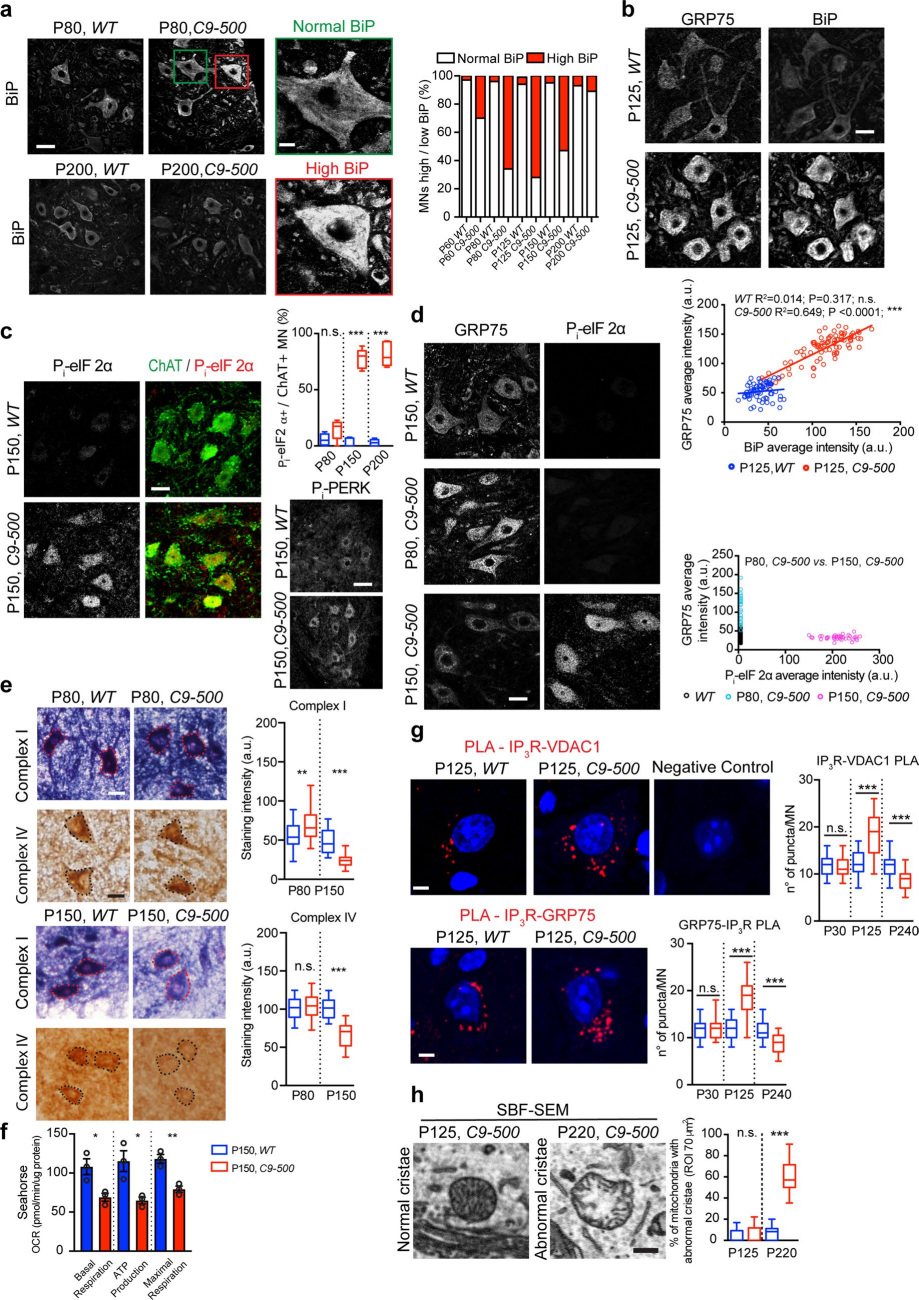
Diminished GRP75 expression and mitochondrial impairments coincide with UPR. **a** Immunolabeling for the ER stress marker BiP in *WT and C9-500* spinal cord reveals an increase in BiP intensity in mutant MNs at P60. As early as P60, high BiP expression is observed in ∼30%, which spreads to more than 70% of MNs by P150 and is subsequently downregulated at P200. *n* = 3 mice/genotype/age. **b** Immunolabeling for GRP75 and BiP in *WT* and *C9-500* spinal cord sections. Bottom: Q.A. indicates a direct correlation between higher levels of BiP and increased expression intensity of GRP75 in *C9-500* MNs at P125: Pearson correlation *WT* *r*^2^ = 0.01412, *P* = 0.3167, *C9-500* *r*^2^ = 0.6486, *P* < 0.0001 (*n* = 73 MNs for *WT* and 86 MNs for *C9-500*, from 3 mice/genotype). **c** Appearance of advanced UPR in mutant MNs as measured by the presence of phosphorylated eIF2α (P_i_-eIF2α) as from P150 (78% ± 3.403) and P_i_-PERK. Right: number of ChAT + ve MNs expressing P_i_-eIF2α analyzed at P80 *WT* n = 68 and *C9-500,* *n* = 73, P150 *WT,* *n* = 76 and *C9-500,* *n* = 71, P200 *WT,* *n* = 192 and *C9-500,* *n* = 151). **d** Representative confocal images of GRP75 and P_i_-eIF2α immunofluorescence staining, depicting high levels of GRP75 levels in *C9-500* animals at P80 when there is no UPR activation, while at P150, when ~ 78% of MNs are positive for P_i_-eIF2α, GRP75 levels are lower compared to *WT*. Bottom right: Q.A. of GRP75 and P_i_-eIF2α intensity (*n* of MNs analyzed *WT*: 66 *C9-500* P80:22, *C9-500* P150:45. **e** Representative images from a colorimetric assay on 20 µm fresh spinal cord sections and relative Q.A. reveal no change in staining intensities for electron transport chain **(**ETC**)** complexes I and IV in *WT and C9-500* MN soma at P80, when GRP75 is upregulated, whereas decreased staining intensity for both complexes is observed in *C9-500* MNs by P150 (Complex I: Unpaired *t* test: *WT* P80, *n* = 36 vs *C9-500* P80, *n* = 32, *t* = 3.095, *P* = 0.0029**, *WT* P150, *n* = 25 vs *C9-500* P150, *n* = 27, *t* = 7.222, *P* < 0.0001***; Complex IV: Unpaired *t* test: *WT* P80, *n* = 21 vs *C9-500* P80, *n* = 21, *t* = 0.2275, *P* = 0.8212 n.s., *WT* P150, *n* = 22 vs *C9-500* P150 *n* = 25, *t* = 8.179, *P* < 0.0001***). 3 independent experiments, *n* = 3 mice/genotype. **f** Oxygen consumption rate measured via seahorse on isolated mitochondria from P150 *WT* and *C9-500* spinal cords present decreased basal respiration, ATP production and maximal respiration in mutant vs *WT* mitochondria. Unpaired *t* test basal respiration: *WT* vs *C9-500* *t* = 0.026, *P* = 0.026*; ATP production: *WT* vs *C9-500* *t* = 3.600, *P* = 0.0228*; maximal respiration: *WT* vs *C9-500* *t* = 5.571, *P* = 0.0051**. **g** Representative confocal images of proximity ligation assay (PLA) between MAM interacting proteins, IP_3_R and VDAC1 and IP3R–GRP75, showing increased number of puncta in *C9-500* animals at P125, when GRP75 is upregulated, and significant decrease at P200, when GRP75 is lost. Q.A. analysis of the number of puncta per MN (PLA: IP_3_R–VDAC1, One-way ANOVA: *F* = 51.52***, Sidak multiple comparison test: *WT* P30, *n* = 38 vs *C9-500* P30, *n* = 39, *t* = 0.157, n.s., *WT* P125, *n* = 37 vs *C9-500* P125, *n* = 41, *t* = 9.34***; *WT* P200, *n* = 39 vs *C9-500* P200, *n* = 39, *t* = 4.84***. (PLA: IP_3_R–GRP75) One-way ANOVA: *F* = 71.56***, Sidak multiple comparison test: *WT* P30, *n* = 40 vs *C9-500* P30, *n* = 40, *t* = 0.0.42, n.s., *WT* P125, *n* = 40 vs *C9-500* P125, *n* = 42, *t* = 12.05***; *WT* P200, *n* = 41 vs *C9-500* P200, *n* = 39, *t* = 4.42***, 3 mice/genotype/time point). **h** Representative 2D SBF–SEM images of mitochondria displaying normal internal cristae at P125 in *WT* MNs and mitochondria with abnormal internal cristae at P220 in *C9-500* MNs. Q.A. of mitochondria with abnormal cristae (Unpaired *t* test, P125 *WT,* *n* = 31 vs P125 *C9-500,* *n* = 31, *t* = 1.54, n.s.; P220 *WT,* *n* = 27 vs P125 *C9-500,* *n* = 31, *t* = 18.08, *P* < 0.0001***). Scale bars **a** 30 and 10 µm (zoom), **b**, **c** and **d** 20 µm, **e** 30 µm, **g** 10 µm, **h** 0.5 µm

Since GRP75 expression was modulated by ER stress in *C9ORF72*-ALS/FTD patient iMNs, we addressed the early phase of ER stress by labeling the *C9-500* mouse spinal cord sections with antibodies against the luminal ER protein BiP/GRP78. From P60, a gradual increase in BiP expression in mutant *C9-500* rodent MNs was observed. From P80 onward, 66–72% of spinal MNs exhibited signs of ER stress as demonstrated by high BiP levels (Fig. 5a). A similar increase in BiP levels was also observed in the cortex of the mice (Supplementary Fig. 6, online resource). Moreover, MNs displaying ER stress also exhibited higher expression levels of GRP75, and a significant direct relationship between high BiP levels and augmented GRP75 expression was observed within spinal MNs at P125 (Fig. 5b, bottom).

To better understand the relationship between ER stress and modulation of GRP75 expression, we induced mild ER stress in *WT* mice for 3 days with TU (0.1 µg/g) IP injections, followed by laser dissection of spinal MNs and qPCR analysis. High levels of *BiP* and *Chop* mRNA indicated ongoing ER stress due to TU treatment. Notably, several-fold upregulation of *Grp75* transcripts was observed in *WT* MNs, reconfirming that GRP75 levels are modulated by ER stress in MNs (Supplementary Fig. 7a, online resource). Elevated immunoreactivities for BiP and GRP75 proteins were observed in TU-treated ventral spinal MNs, and a correlation between high BiP levels and elevated GRP75 levels was present (Supplementary Fig. 7b, online resource). Interestingly, spinal MNs reacted most strongly to TU-induced ER stress, as both BiP and GRP75 levels in other spinal neurons (ChAT-negative) increased only very moderately (Supplementary Fig. 7c, online resource), again emphasizing the higher vulnerability of spinal MNs to ER stress.

Next, we focused our attention on P150, a time point when an abrupt loss in GRP75 expression is observed. P_i_-eIF2α immunoreactivity, which was hardly noticeable at P80, was prominent at P150 (78.2 ± 3.4%) and P200 (80.9 ± 4.317), and restricted in expression within ChAT-positive spinal ventral horn MNs (Fig. 5c). Moreover, the increased expression of phosphorylated PERK (P_i_-PERK) in *C9-500* MNs confirmed UPR signaling mediated via the PERK pathway (Fig. 5c, bottom right). Notably, when MNs transited to UPR signaling, as observed in P150 *C9-500* mice, those MNs also displayed negligible GRP75 expression and an inverse correlation was observed between higher P_i_-eIF2α expression and GRP75 levels (Fig. 5d), suggesting an ER stress state-dependent modulation of GRP75 expression.

We examined the impact of reduced GRP75 expression on mitochondrial function by a colorimetric assay specifically assessing electron transport chain (ETC) activity. At P80, complex I and IV exhibited normal activity comparable to *WT* MNs. However, at P150 coinciding with UPR, both complexes were strongly reduced within MNs (Fig. 5e). Further, we confirmed by seahorse measurements deficits in key mitochondrial respiration states within P150 *C9-500* lumbar neurons (Fig. 5f). Importantly, the timing of mitochondrial dysfunction coincided with the expression levels of GRP75, which are transiently increased between P60–P125, followed by a significant reduction at P150. As GRP75 physically interacts with both IP_3_R and VDAC1, we assessed this interaction via proximity ligation assay (PLA). While at P30, no difference in the number of IP_3_R–VDAC1 positive puncta was observed in *C9-500* MNs compared with *WT* MNs, a significant increase in IP_3_R–VDAC1 association was observed at P125, followed by a dramatic reduction in interaction at P240 in *C9-500* spinal MNs (Fig. 5g). As GRP75 is mainly localized to mitochondria, we evaluated its interaction at the MAM, which it normally achieves by binding to IP_3_R, located on the ER membrane. PLA measurements for GRP75–IP_3_R association revealed a similar time course of interaction as observed for IP_3_R–VDAC1 (Fig. 5g).

We next performed ultrastructural analyses of mitochondria in MNs by SBF–SEM and 3D-reconstruction of the images. Focusing on mitochondrial morphology in both conditions, we found that the *WT* MN soma presented mainly tubular and elongated mitochondria, whereas such tubular and elongated mitochondria were negligibly present in mutant MNs at P125 (Supplementary Fig. 8a, online resource). Instead, nearly spherical mitochondria were abundantly seen in mutant P125 *C9-500* MNs. In fact, *C9-500* MNs presented an overall higher number of mitochondria, and, in line with our iMN data (see Fig. 2a, b), a significant percentage of those mitochondria were rounded in shape, when the sphericity of mitochondria was examined at P60 and P125 (Supplementary Fig. 8b, online resource). In line with our observations in iMNs (Fig. 2d), we found that spherical mitochondria on average exhibited increased contact lengths as well as a higher number of contact points with the ER membrane in mutant MNs (Supplementary Fig. 8c, d, online resource). Analyzing mitochondrial integrity revealed that despite being rounded, mitochondria in P125 *C9orf72* MNs presented no increase in abnormal cristae compared to *WT* MNs. However, by P220, an age coinciding with drastically reduced GRP75 levels, nearly 80% of all mitochondria lacked intact cristae in mutant MNs, thus indicating impairment in mitochondrial integrity and function (Fig. 5h).

### PolyGA sequesters GRP75 and impairs its localization at the MAM

**Fig. 6:**
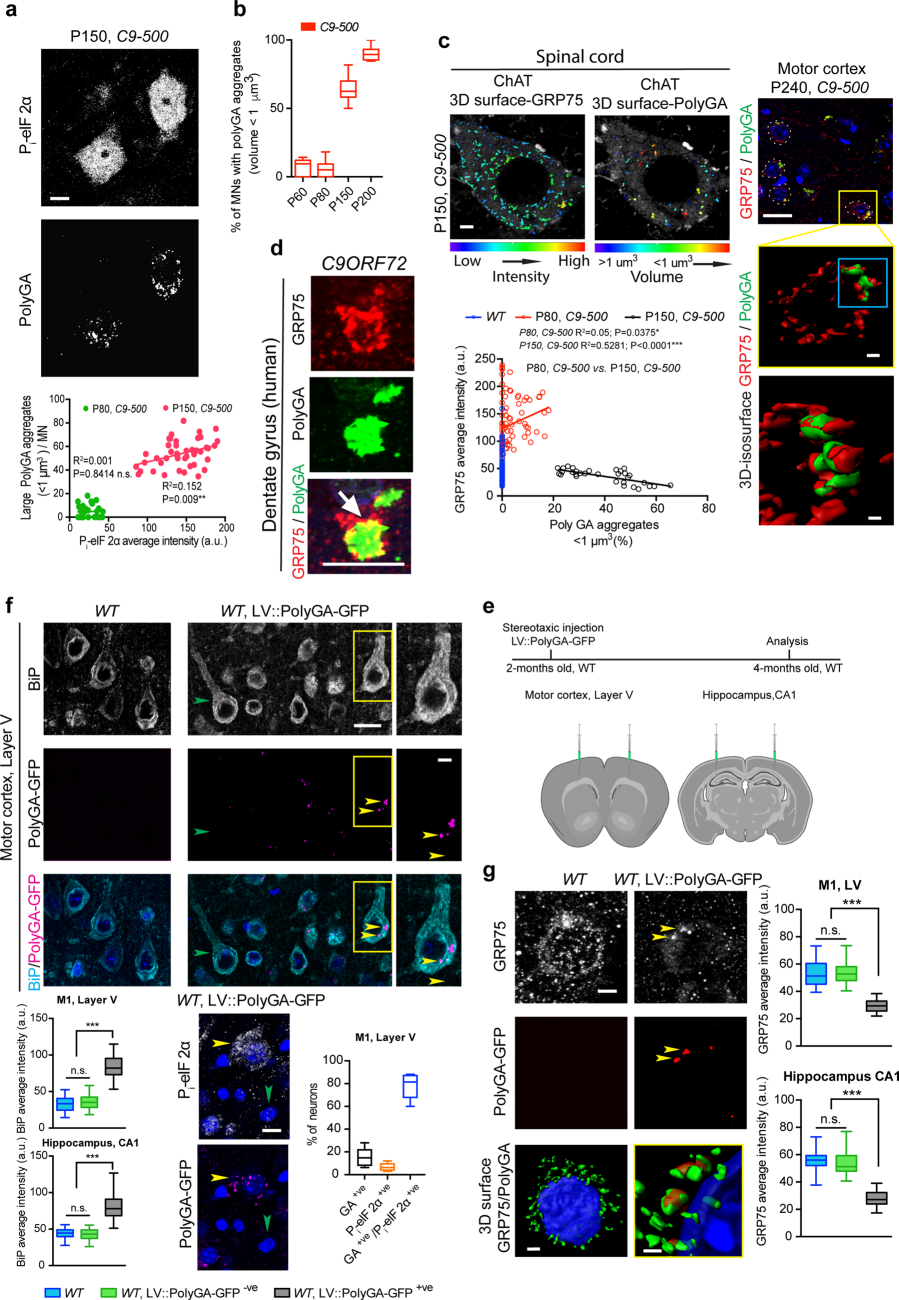
PolyGA expression and sequestration of GRP75 correlates with UPR appearance in *C9-500* MNs. **a** Immunolabeling for P_i_-eIF2α and PolyGA at P80 and P150 reveals prominent PolyGA aggregates in *C9-500* spinal MNs at P150. Q.A. indicates a direct correlation between higher levels of P_i_-eIF2α and large PolyGA aggregates. Each dot represents average intensity values within one MN. Pearson correlation P80: *r*^2^ = 0.001454, *P* = 0.8414, n.s., P150: *r*^2^ = 0.1519, *P* = 0.009**. *n* = 3 mice/genotype/age. **b** Q.A of percentage of spinal MNs with PolyGA aggregates with volume larger than 1 μm^3^ in *C9-500* mice across different disease stages (mean ± SEM P60 = 14.67 ± 1.733; P80 = 5.720 ± 1.110; P150 = 64.19 ± 3.801; P200 = 90.02 ± 76 2.232, One-way ANOVA: *F* = 377.8, *P* < 0.0001***, P60 vs P80 *t* = 0.5319 n.s., P80 vs P150 *t* = 21.03***, P150 vs P200 *t* = 7.057***). **c** Representative 3D rendering for GRP75 and PolyGA expression within ChAT positive spinal MNs in *C9-500* mice at P80 and P150. Four z stacks at distance of 0.5 µm each were used for this rendering, revealing that at P150 with the appearance of PolyGA expression, intensity of GRP75 immunopositivity decreased. Bottom: Q.A. revealed an inverse correlation between GRP75 expression and PolyGA aggregates. Pearson correlation: P80: *r*^2^ = 0.05, *P* = 0.0375*; P150: *r*^2^ = 0.5281, *P* < 0.0001***. Right: Confocal image of *C9-500* motor cortex, stained for GRP75 and PolyGA, depicting GRP75 sequestered together with PolyGA aggregates and 3D volume reconstruction. **d** Representative immunofluorescence staining for GRP75 and PolyGA in human *C9ORF72* ALS patient hippocampal dentate gyrus showing GRP75 sequestration with PolyGA aggregate. **e** Experimental timeline for lentiviral (LV) PolyGA-GFP (LV::PolyGA-GFP) injection in the motor cortex and CA1 hippocampus of *WT* animals. **f** Representative images of layer V motor cortex stained for BiP and PolyGA-GFP in *WT* control and *WT* LV::PolyGA transduced neurons. Note that neurons expressing PolyGA-GFP show increased levels of ER stress (yellow arrows). Bottom: representative confocal images of motor cortex from *WT* LV::PolyGA-GFP showing GA aggregates colocalizing with P_i_-eIF2α (yellow arrow) and neurons spared from infection (green arrow). Percentage of neurons positive only for PolyGA: 15.01 ± 3.797; only P_i_-eIF2α: 6.693 ± 1.657 and double positive PolyGA/P_i_-eIF2α: 78.30 ± 5.218. Q.A. of BiP expression in *WT* control and *WT* LV::PolyGA in motor cortex and hippocampus reveals increased expression of BiP in infected neurons: unpaired *t* test Motor cortex: *WT* vs *WT* LV::PolyGA-GFP − ve *t* = 1.065, *P* = 0.2913 n.s., *WT* vs *WT* LV::PolyGA-GFP + ve *t* = 15.88, *P* < 0.0001***; unpaired *t* test hippocampus: *WT* vs *WT* LV::PolyGA-GFP − ve *t* = 0.4432, *P* = 0.6592 n.s., *WT* vs *WT* LV::PolyGA-GFP + ve *t* = 11.87, *P* < 0.0001***. **g** Representative images depicting GRP75 expression pattern in *WT* and *WT* LV::PolyGA-GFP, while *WT* show cytoplasmic GRP75 immunolabeling, neurons infected with LV::PolyGA-GFP show reduced GRP75 expression as well as GRP75 sequestration around the aggregates (yellow arrows) as represented in the 3D reconstruction. Q.A unpaired *t* test Motor cortex: *WT* vs *WT* LV::PolyGA-GFP − ve *t* = 0.3635, *P* = 0.7178 n.s., *WT* vs *WT* LV:: PolyGA-GFP + ve *t* = 11.43, *P* < 0.0001***; unpaired *t* test hippocampus: *WT* vs *WT* LV:: PolyGA-GFP − ve *t* = 0.8712, *P* = 0.3871 n.s.; *WT* vs *WT* LV::PolyGA-GFP + ve *t* = 15.80, *P* < 0.0001***. Scale bars: **a** and **f** 20 µm, (zoom 5 µm), **c** 5 µm and 30 µm (zoom 3 and 2 µm), **d** 8 µm, **g** 5 µm (zoom 3 and 2 µm)

Several studies have implicated the link between toxic DPRs, UPR markers, and neurodegeneration [14, 68] and recently highlighted the presence of both DPRs and UPR markers in human *C9ORF72*-ALS/FTD post-mortem brain [22]. Given that our data had revealed that in the human post-mortem hippocampus, PolyGA co-localizes with GRP75, indicating the possibility of aberrant GRP75 sequestration by PolyGA, we assessed PolyGA expression in *C9-500* MNs. At P150, but not at P80, *C9-500* MNs presenting the UPR marker P_i_-eIF2α also displayed an accumulation of PolyGA (Fig. 6a). Furthermore, as previously shown [11], we found a direct correlation between large PolyGA aggregates (volume > 1 µm^3^) and the expression of P_i_-eIF2α in MNs, which became highly significant by P150 in mutant MNs coinciding with widespread UPR in MNs compared with P80, when highest GRP75 expression is observed. Notably, longitudinal measurement of PolyGA aggregate size within ChAT positive neurons revealed that between P150-P200 nearly 64–90% of all MNs harbored large PolyGA aggregates (Fig. 6b). Consequently, we examined the relationship between PolyGA aggregates and reduced GRP75 expression at P150. We found that spinal MNs with large PolyGA aggregates mainly showed lower GRP75 levels when compared to an age (P80) when GRP75 levels are elevated (Fig. 6c, bottom graph). Strikingly, cortical neurons of aged (P240) *C9-500* mice exhibited PolyGA aggregates co-localizing with GRP75 (Fig. 6c, right). Similarly, in both human postmortem ALS tissue (Fig. 6d) and *C9-500* rodent neurons (Fig. 6c, lower right panel) GRP75 was prone to surround PolyGA aggregates.

To further demonstrate that these observations were not mouse model-specific, we generated a lentiviral construct expressing GFP-tagged PolyGA (LV::PolyGA-GFP) harboring 149 repeats under synaptophysin promoter and injected the virus into the cortical layer V and the hippocampus CA1 region of *WT* animals (see scheme Fig. 6e). Analysis after 2 months, revealed robust expression of PolyGA both in the layer V upper MNs and in the hippocampus (Supplementary Fig. 9a, online resource). Both cortical and hippocampal CA1 neurons expressing PolyGA aggregates presented higher BiP expression levels compared with non-infected *WT* neurons, as well as displayed UPR (78.3 ± 5.2% of PolyGA transduced neurons), measured by P_i_-eIF2α immunoreactivity (Fig. 6f). Notably, PolyGA-expressing neurons were largely devoid of cytoplasmic GRP75 and PolyGA aggregates were surrounded by the remaining GRP75, consistent with sequestration of GRP75 (Fig. 6g and Supplementary Fig. 9b, online resource). To exclude that changes in BiP or GRP75 expression were due to lentiviral transduction or expression of GFP, cortical neurons were infected with LV::GFP, which did not lead to any significant alteration in GRP75 or BiP expression, suggesting that the observed changes were PolyGA-dependent (Supplementary Fig. 9c, online resource).

### PolyGA impairs GRP75 function at the MAM, thereby compromising mitochondrial Ca^2+^ uptake

**Fig. 7:**
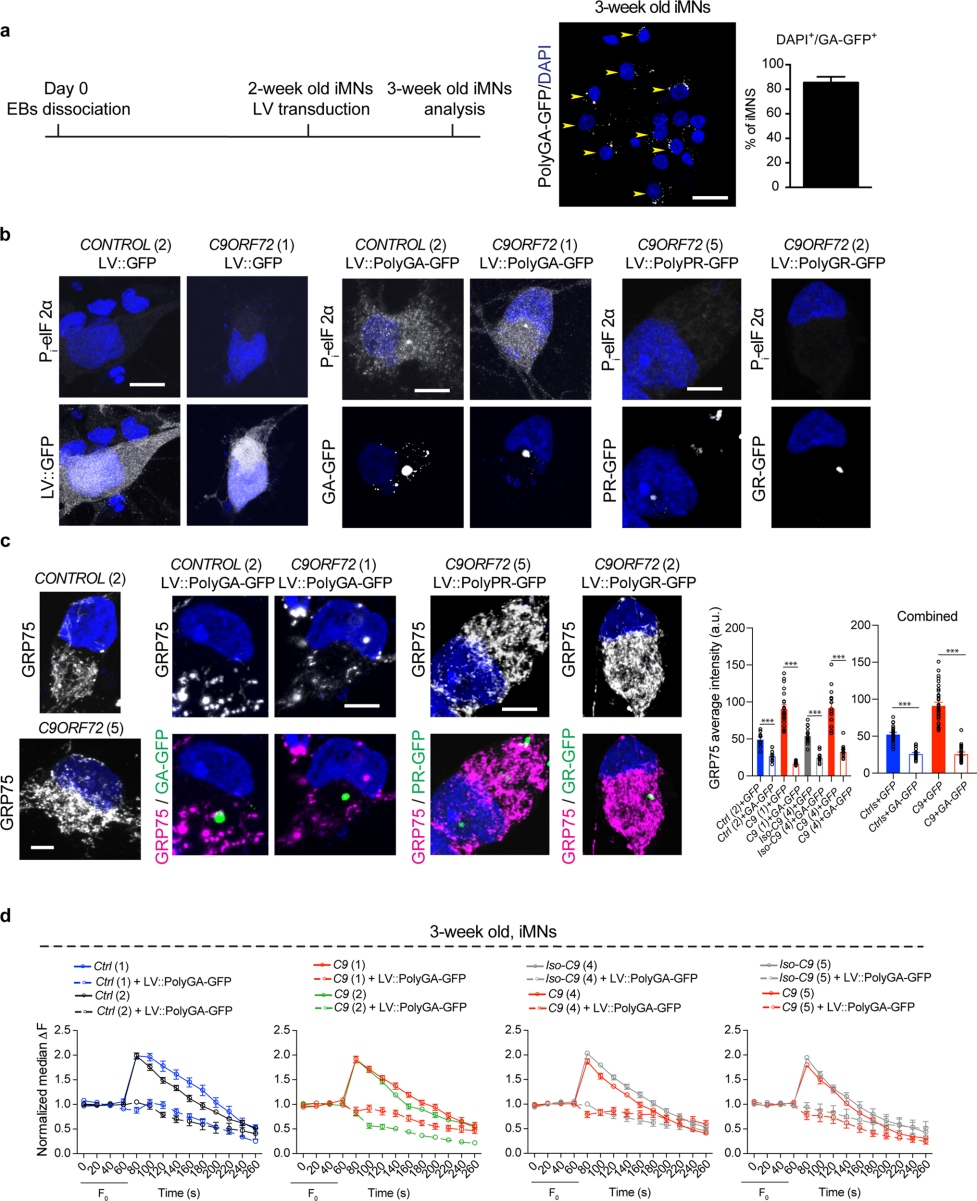
PolyGA impacts GRP75 levels, disrupting mitochondrial Ca^2+^ uptake in iMNs. **a** Experimental timeline for LV transduction of iMNs and Q.A. of LV::PolyGA transduction efficiency in iMNs. 85.46% of iMNs show PolyGA aggregates after LV transduction. **b** iMNs transduced with LV::PolyGA-GFP show increased appearance of UPR compared to control-GFP and LV::PolyPR-GFP, LV::PolyGR-GFP transduced iMNs. **c** Representative images of GRP75 expression in iMNs and iMNs transduced with LV::GFP; LV::PolyGA-GFP, LV::PolyPR-GFP and LV::PolyGR-GFP, while untransduced iMNs as well as the ones transduced with LV::PolyPR-GFP and LV::PolyGR-GFP show normal GRP75 expression, iMNs transduced with LV::PolyGA-GFP are devoid of GRP75. Right: Q.A. of GRP75 expression from LV::PolyGA-GFP transduced neurons and LV::GFP transduced neurons. Unpaired *t* test: *Ctrl(2)* + LV::PolyGA-GFP vs *Ctrl(2)* + LV::GFP *t* = 6.465, *P* < 0.0001***; *C9(1)* + LV::PolyGA-GFP vs *C9(1)* + LV::GFP *t* = 10.20, *P* < 0.0001***; *Iso-C9(4)* + LV::PolyGA-GFP vs *Iso-C9(4)* + LV::GFP *t* = 8.287, *P* < 0.0001***; *C9(4)* + LV::PolyGA-GFP vs *C9(4)* + LV::GFP *t* = 7.980, *P* < 0.0001***. Combined graph for control lines (*Ctrl(2) and Iso-C9(4)*) and *C9(1–4*): unpaired *t* test *Ctrls* + LV::PolyGA-GFP vs *Ctrls* + LV::GFP *t* = 10.76, *P* < 0.0001***; *C9* + LV::PolyGA-GFP vs *C9* + LV::GFP *t* = 12.51, *P* < 0.0001***. **d** Baseline (0–60 s) and stimulated mitochondrial Ca^2+^ uptake (80 s onward) traces from *Ctrls(1 and 2)* and *C9ORF72(1,2)* iMNs and *Iso-C9ORF72(4 and 5) and C9ORF72(4 and 5)* iMNs after LV transduction. All the MNs lines show deficit in mitochondria Ca^2+^ transient when transduced with LV::PolyGA-GFP compared to not transduced controls. (Number of iMNs: *Ctrl(1)*: 10, *Ctrl(1)* + LV::PolyGA-GFP: 17, *Ctrl(2)*: 12, *Ctrl(2)* + LV::PolyGA-GFP: 11, *C9(1)*: 10, *C9(1)* + LV::PolyGA-GFP: 12, *C9(2)*:11, *C9(2)* + LV::PolyGA-GFP: 11, *iso-C9(4)*: 11, *iso-C9(4)* + LV::PolyGA–GFP: 10; *C9(4)*: 10, *C9(4)* + LV::PolyGA–GFP: 13; *iso-C9(5)*: 10, *iso-C9(5)* + LV::PolyGA–GFP: 11; *C9(5)*: 10, *C9(5)* + LV::PolyGA–GFP: 11). Multiple *t* test at 80 s: *Ctrl(1)* mean = 1.99 vs *Ctrl(1)* + LV::PolyGA–GFP mean = 0.88, *P* < 0.0001; *Ctrl(2)* mean = 1.98 vs *Ctrl(2)* + LV::PolyGA–GFP mean = 1.04, *P* < 0.0001; *C9(1)* mean = 1.91 vs *C9(1)* + LV::PolyGA–GFP mean = 0.86, *P* < 0.0001, *C9(2)* mean = 1.91 vs *C9(2)* + LV::PolyGA–GFP mean = 0.82, *P* < 0.0001, *Iso-C9(4)* mean: 2.03 vs *Iso-C9(4)* + LV::PolyGA–GFP mean: 0.79, *P* < 0.0001; *C9(4)* mean = 1.87 vs *C9(4)* + LV::PolyGA–GFP mean = 1.00, *P* < 0.0001, *Iso-C9(5)* mean: 1.94 vs *iso-C9(5)* + LV::PolyGA–GFP mean: 0.94, *P* < 0.0001; *C9(5)* mean = 1.79 vs *C9(5)* + LV::PolyGA–GFP mean = 0.77, *P* < 0.0001. Scale bars: **a** 25 µm, **b** 5 μm, **c** 10 μm

Drawing from these observations, we assessed the influence of PolyGA on GRP75-mediated normalization of mitochondrial Ca^2+^ uptake in iMNs. Because we could not detect PolyGA aggregates in our iMN cultures until 4 weeks of differentiation, we decided to infect iMNs with LV::PolyGA-GFP, LV::PolyPR-GFP, LV::PolyGR-GFP, or LV::GFP (Fig. 7a for experimental design). We transduced 2-week-old iMNs (transduction efficiency of more than 85%) (Fig. 7a), as at this stage, high GRP75 expression levels as well as almost normally functioning mitochondria are present. In line with a previous study [68], only PolyGA, but not PolyPR or PolyGR-transduced iMNs displayed robust UPR signaling via the appearance of P_i_-eIF2α (Fig. 7b). Notably, PolyGA-expressing iMNs showed significantly reduced GRP75 expression compared with PolyPR or PolyGR aggregate-bearing iMNs. Importantly, this reduction was irrespective of whether they were healthy/isogenic controls or *C9ORF72*-derived iMNs (Fig. 7c). As our data suggested that PolyGA expression negatively impacted GRP75 levels, we subsequently, performed mitochondrial Ca^2+^ imaging on LV::PolyGA-GFP transduced iMNs. We could not use LV::GFP as mitochondrial Ca^2+^ measurements involve two classical fluorophores measuring simultaneously mitochondrial Ca^2+^ uptake as well as its membrane potential. Strikingly, PolyGA expression led to impaired mitochondrial Ca^2+^ uptake in healthy iMNs as well as in all *C9ORF72* iMN lines compared with non-transduced iMNs (Fig. 7d). To understand how PolyGA affects mitochondria at a structural level, SBF–SEM was performed on healthy control iMN lines, which were transduced with LV::PolyGA-GFP. Large PolyGA aggregates harboring various organelle-like structures were observed (yellow box); rounded mitochondria were atypically clustered around the nucleus (red box), with depletion of mitochondria from the cytoplasmic region of the iMN (Supplementary Fig. 10, online resource), indicating abnormal distribution of mitochondria in PolyGA aggregate bearing neurons.

### Overexpression of GRP75 rescues ER stress and mitochondrial deficits

**Fig. 8:**
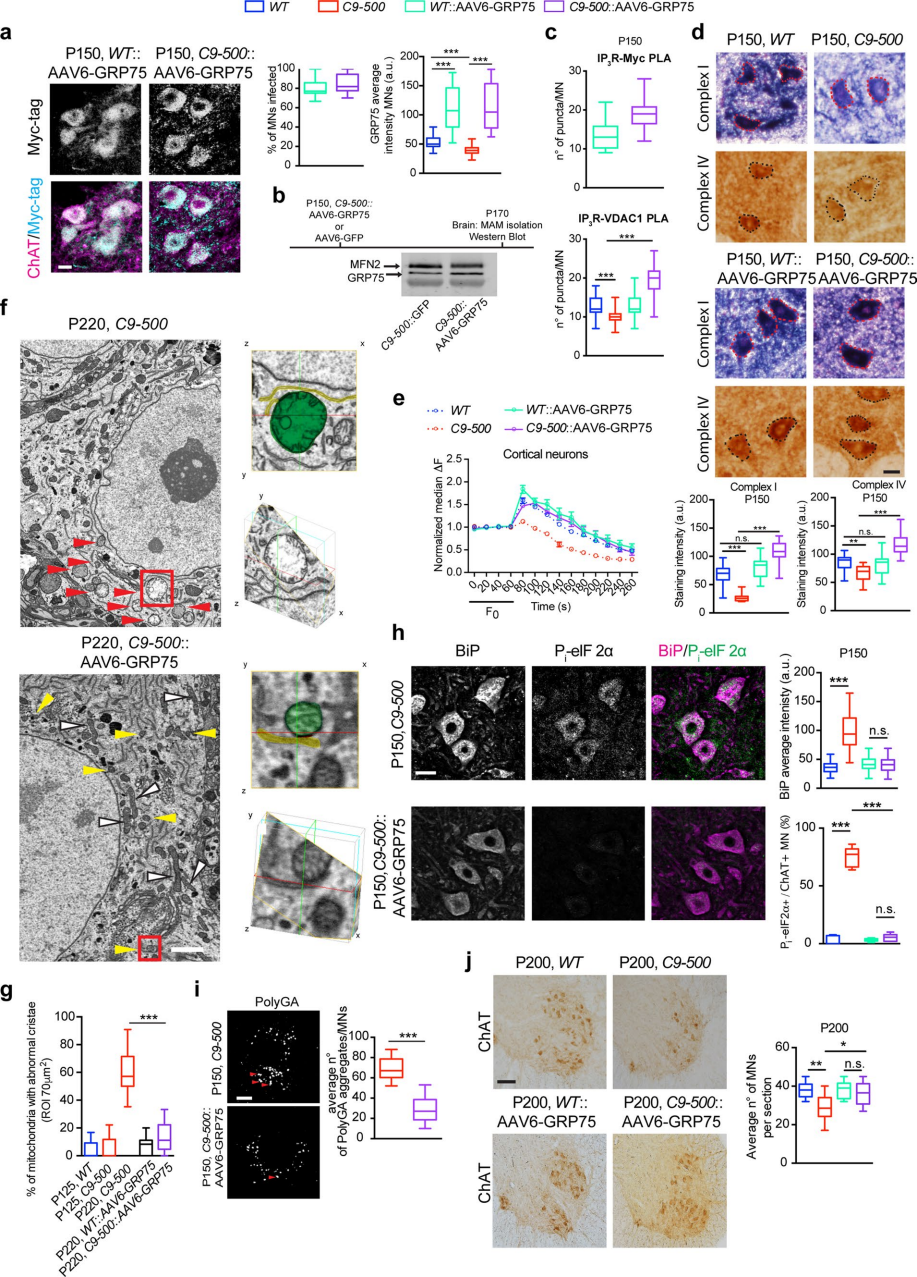
Overexpression of GRP75 protects MNs and ameliorates disease symptoms. **a** Dual immunolabeling for ChAT and Myc-tag in AAV6–GRP75 transduced spinal cord reveals robust expression of GRP75 in *C9-500* and *WT* MNs. Right: Q.A. of percentage of MNs infected with the virus. (3 mice/genotype). Q.A. of GRP75 intensity levels in Myc + ve MNs (one-way ANOVA F = 184.1, *P* < 0.0001***, Sidak multiple comparison test: *WT* *n* = 54 vs *C9-500* *n* = 55, *t* = 2.393***; *C9-500* *n* = 54 vs *C9-500*::AAV6–GRP75 *n* = 69, *t* = 14.91***). **b** Experimental timeline for stereotaxic injection of AAV6–GRP75 in adult *C9-500* mice. Representative immunoblot from MAM isolated fraction displaying increased GRP75 within MAM fraction in *C9-500* overexpressing brains. No change is observed in the internal control MFN2. **c** Q.A. of proximity ligation assay for IP_3_R–VDAC1 and Myc–IP_3_R performed on *WT* and *C9-500*::AAV6–GRP75 injected animals. After GRP75 overexpression the number of contacts between IP_3_R–VDAC1 are significantly increased in *C9-500* animals compared to age-matched controls. Unpaired *t* test Myc–IP_3_R *t* = 5.810, *P* < 0.0001***; One-way ANOVA IP_3_R–VDAC1: *F* = 65.07, *P* < 0.0001***, Sidak’s multiple comparison test: *WT* vs C9-500***; *WT* vs *WT* AAV6–GRP75 n.s.; *C9-500* vs *C9-500* AAV6–GRP75***. **d** Representative images of ETC complexes I and IV in *C9-500* MN soma showing increased staining intensities after GRP75 overexpression and relative Q.A. of staining intensity (Complex I, One-way ANOVA: *F* = 76.59, *P* < 0.0001***, Sidak multiple comparison test: *WT* P150, *n* = 16 vs *C9-500* P150, *n* = 16, *t* = 7.139***, *C9-500* P150, *n* = 16 vs *C9-500-*AAV6*–*GRP75 P150, *n* = 19, *t* = 14.92***; Complex IV, One-way ANOVA: *F* = 29.74, *P* < 0.0001***, Sidak multiple comparison test: *WT* P150, *n* = 19 vs *C9-500* P150, *n* = 16, *t* = 3.324***, *C9-500* P150, *n* = 16 vs *C9-500-*AAV6*–*GRP75 P150, *n* = 19, *t* = 9.068***). 3 experiments from 3 mice/genotype. **e** Baseline and stimulated mitochondrial Ca^2+^ uptake traces from *WT* and *C9-500* naïve cortical neurons (dotted lines) and AAV6–GRP75 transduced cortical neurons (bold lines). Overexpression of GRP75 significantly ameliorated and normalized Ca^2+^ uptake in mitochondria of *C9-500* cortical neurons (number of neurons *WT*: 22, *C9-500*: 24, *WT*::AAV6*–*GRP75: 21, *C9-500*::AAV6*–*GRP75: 22; multiple *t* test at 80 s *C9-500* mean = 1.120, *C9-500*::AAV6–GRP75 mean = 1.469, *P* < 0.0001; 100 s: *C9-500* mean = 0.967, *C9-500*::AAV6–GRP75 mean = 1.530, *P* < 0.0001). **f** Representative images of a 2D SBF–SEM analysis of *C9-500* spinal MNs at P220: rounded, swollen mitochondria disconnected from the ER showing rudimentary cristae (red arrowheads). Such abnormal mitochondria are not present in aged-matched *C9-500* animals overexpressing GRP75, where white arrowheads mark tubular mitochondria and yellow arrowheads mark round mitochondria in close contact with the ER membranes. Right: Higher magnification volume views of two P220 *C9-500* mitochondria, one of which is marked by the red rectangle in the left picture. Below: aged matched *C9-500*::AAV6–GRP75 mitochondria displaying normal architecture of cristae and normal ER–mitochondria contact. **g** Q.A. of the percentage of mitochondria displaying abnormal cristae (One-way ANOVA *F* = 170.0, *P* < 0.0001***, Sidak’s multiple comparison test: P220 *C9-500* vs P220 *C9-500*::AAV6–GRP75, *t* = 18.09***). **h** Representative images of BiP and P_i_-eIF2α immunoreactivity showing decreased levels of ER stress and UPR in *C9-500* animals after GRP75 overexpression. Q.A. indicates normalized BiP levels (unpaired *t* test, MN numbers P150 *WT* *n* = 41 vs P150 *C9-500* *n* = 53, *t* = 12.74, *P* < 0.0001***; P150 *WT*::AAV6–GRP75, *n* = 48 vs P150 *C9-500*::AAV6–GRP75, *n* = 61, *t* = 0.7378, *P* = 0.4623 n.s.), and low levels of BiP coincide with the absence of UPR signaling (3 mice/genotype/age). **i** Immunofluorescence for PolyGA aggregates reveals a reduced number of PolyGA aggregates in *C9-500*::AAV6–GRP75 MNs (unpaired *t* test P150: *C9-500* *n* = 24 vs *C9-500*::AAV6–GRP75 *n* = 32, *t* = 12.94, *P* < 0.0001***). **j** Immunohistochemical staining for the MN marker ChAT reveals approximately 30% reduction in MN numbers in mutant *C9-500* spinal cord. Sustained MN numbers observed in *C9-500* spinal cord transduced with AAV6–GRP75 (*n* = 8–15 consecutive sections from the lumbar region of 3–5 mice). Unpaired *t* test, *WT* mean = 38.00 ± 1.344 vs *C9-500* 28.70 ± 2.161, *t* = 3.558**, *P* = 0.0024; *WT*::AAV6–GRP75 mean = 38.00 ± 1.509 vs *C9-500*::AAV6–GRP75 mean = 36.13 ± 2142 *t* = 0.7378, n.s. *P* = 0.4775). *n* = 6 mice/genotype. Scale bars **a**, **d** 30 µm, **f** 2.5 μm, **i** 10 μm,** j** 150 µm

While early ER stress-mediated adaptive responses induce elevated expression of GRP75, thereby aiding in sustaining mitochondrial function, however, chronic ER stress eventually leads to the initiation of apoptosis [25]. Thus, we assessed whether the overexpression of GRP75 would support ER–mitochondrial association, thereby promoting ER and mitochondrial function and decreasing PolyGA load. To this end, neonatal *C9-500* mice were infected with AAV6–GRP75 via i.c.v. injection. As the cloning vector had a myc-tag, we probed for exogenous GRP75 expression by immunostainig for myc. A significant fraction (84.6 ± 2.4%) of lumbar MNs were immunoreactive for myc and expressed elevated amounts of GRP75. Further, MAM isolation and immunoblotting for GRP75 revealed higher amounts of GRP75 at the MAM (Fig. 8b). Overexpression of AAV6–GRP75 in mutant MNs from birth on led to enhanced IP_3_R–VDAC1 interactions in GRP75 overexpressing *C9-500* MNs. To confirm that increased IP_3_R–VDAC1 association was due to the interaction of AAV6–GRP75 with IP_3_R, we measured Myc–IP_3_R associations via PLA, which were prominently increased in *C9-500* MNs. (Fig. 8c). Next, we examined ETC levels as a readout for mitochondrial function, and found that GRP75 overexpression in symptomatic *C9-500* MNs restored levels of Complex I and Complex IV compared with age-matched untreated *C9-500* MNs (Fig. 8d). Further, to assess the impact of GRP75 overexpression on mitochondrial Ca^2+^ transients, we overexpressed GRP75 in cultured cortical neurons from *C9-500* mice. As expected, mitochondrial Ca^2+^ uptake was strongly impaired in non-transduced cortical neurons, but GRP75 overexpression led to normalized Ca2^+^ transients (Fig. 8e).

To gain more insights into the ameliorated mitochondrial phenotype at a structural level, SBF–SEM was performed on *C9-500* spinal cord at P220, when endogenous GRP75 expression is significantly reduced. *C9-500* MNs at P220 presented clusters of rounded mitochondria lacking cristae (red arrows), whereas *C9-500* MNs overexpressing GRP75 exhibited both elongated (white arrows) and rounded mitochondria (yellow arrows) at comparable levels (Fig. 8f). Importantly, a significant proportion of mitochondria presented cristae abnormalities or their complete loss in *C9-500* MNs when compared with AAV6–GRP75 overexpressing *C9-500* MNs (Fig. 8g). We also examined mitochondrial cristae at P125, when higher endogenous GRP75 expression is observed; at this time point, mitochondrial cristae in *C9-500* MNs were unchanged compared to *WT* MNs. Subsequently, we analyzed ER stress and detected normalized BiP levels and absence of UPR signaling in mutant MNs overexpressing GRP75 (Fig. 8h). Moreover, we found that GRP75 overexpression also diminished the number of large PolyGA aggregates in *C9-500* MNs (Fig. 8i). Consequently, we determined whether restoring ER and mitochondrial homeostasis via GRP75 overexpression is neuroprotective. Counting MN numbers at P220 in *C9-500* spinal cord expressing AAV6–GRP75 revealed unchanged MN numbers compared with *WT* mice. In comparison, a 30% reduction (*P* = 0.0024) in MN numbers, indicative of ongoing MN degeneration, was observed in untreated *C9-500* spinal cord (Fig. 8j), suggesting that high GRP75 expression provides neuroprotection in *C9ORF72*-ALS/FTD.

At the NMJ, fiber-typing analysis (ATP pH 9.4) revealed that the Gastrocnemius muscle (GC) of *C9-500* mice had lost the checkerboard pattern due to the loss of type 2 fibers. Conversely, in GC muscle of GRP75 overexpressing *C9-500* mice, the checkerboard pattern was mostly preserved and comparable to *WT* (Supplementary Fig. 11a, online resource, left panel). Interestingly, NADH staining, an oxidative enzyme staining procedure that reveals myofibrillar architecture and mitochondria revealed the most striking difference between the three groups. Notably, different strong stains of individual myocytes in *WT* reflecting varied fiber types was observed. In *C9-500*, fiber typing was negligible with only few intensely stained fibers mainly being atrophic and/or angulated, compatible with denervation and a loss of type 2 fibers. In contrast, GC muscle of GRP75 overexpressing mutant mice, exhibited overall more intensely stained fibers and lacking checkerboard appearance across many myocyte bundles, but without clearly atrophic/angulated fibers. The intense staining was also prominent in the subsarcolemmal region. These findings are compatible with reduced signs of denervation and loss of fiber type 2, and increased mitochondrial proliferation (Supplementary Fig. 11a, online resource, right panel). Subsequently, we measured muscle endurance of GRP75 overexpressing *C9-500* mice via hanging wire test, which revealed an amelioration in muscle endurance evaluated as time taken to first fall and number of falls within a 2-min period (Supplementary Fig. 11b, online resource). Motor coordination was examined using a reverse rotarod protocol, and significant improvement in motor performance was observed in GRP75 overexpressing *C9-500* mice (Supplementary Fig. 11c, online resource). Finally, hind limb clasping as described in [53] was measured revealing a significant delay in the appearance and progression of hind limb clasping within the GRP75 overexpressing *C9-500* cohort (Supplementary Fig. 11d, online resource). Notably, lifespan increased by approximately 40% for *C9-500* mice overexpressing GRP75 (Supplementary Fig. 11e, online resource). Overall, our data indicate that higher GRP75 levels, significantly delay symptomatic motor deficits and prolong lifespan in *C9-500* mice.

## Discussion

Identifying early disease coping mechanisms conferring neuronal resilience is expected to lead to effective treatments for NDs. In this study, employing patient-derived MNs and the *C9-500* mouse model of ALS, we found that the transient upregulation of GRP75, a mitochondrial associated membrane (MAM) protein, is an ER stress-induced adaptive response. Mechanistically, this GRP75 increase facilitates ER–mitochondrial coupling, thus sustaining mitochondrial Ca^2+^ uptake and efficient function (Supplementary Fig. 12, online resource). It is well-documented that GRP75 expression is modulated by ER stress, as both BiP and GRP75 genes contain ER stress response element (ERSE) on their promoters, these can simultaneously undergo transcription in response to ER stress signals [38]. Notably, previous studies indicate that the function of GRP75 likely depends upon intrinsic neuronal vulnerabilities and the type of stressor. Under physiological conditions, GRP75 inhibition activates mitochondrial stress responses [7]. However, during pathological disease states in Parkinson’s disease, increased GRP75 expression in neuronal cells prevented mitochondrial dysfunction and cell death [8], whereas in human dopaminergic neurons, GRP75 overexpression enhanced the cytotoxic effects of the mitochondrial complex I inhibitor rotenone [31]. Similarly, in cells exhibiting oxidative stress, GRP75 potentiated cell death via mitochondrial Ca^2+^ overload [26]. Our results indicate that elevated endogenous expression of GRP75 promotes mitochondrial function via normalized mitochondrial Ca^2+^ uptake in *C9orf72* neurons (Fig. 8d, e), suggesting a key protective role for GRP75 during the initial phase of the disease. Our data also highlight the notion that “younger” neurons with relatively preserved ER–MAM can utilize GRP75-mediated specific molecular mechanisms to protect themselves, whereas in older neurons, reduced GRP75 expression compromises this ability.

An important finding from our study was the identification of distinct early impairments in *C9ORF72* mitochondrial ability to uptake Ca^2+^ and of overall reduced ATP levels in mutant MNs (Fig. 3). These impairments occurred before the appearance of pathological hallmarks, suggesting that they might define an intrinsic vulnerability pathway. Conserved mitochondrial functional deficits are observed within *C9ORF72* iPSCs, hinting towards *C9ORF72* mutation as a key component of this intrinsic vulnerability. In addition, oxidative stress, impaired basal and maximal mitochondrial respiration and the direct binding of toxic PolyGR to Atp5a1, causing mitochondrial functional deficits, have recently been demonstrated in *C9ORF72*-ALS/FTD; however, it is not clear how early these deficits manifest in human patients [12, 42, 48]. We found that PolyGA aggregate-bearing human postmortem *C9ORF72*-ALS/FTD patient hippocampal dentate gyrus neurons display reduced expression of GRP75. In human *C9ORF72*-ALS/FTD patient lumbar spinal cord, where PolyGA inclusions are less abundant and pTDP-43 pathology predominates, MNs harboring pTDP-43 aggregates displayed reduced GRP75 immunoreactivity. These findings are interesting considering the variability of TDP-43 aggregate pathology [4–6]. Elevated levels of GRP75 together with its co-localization mostly with the small granular, early pTDP-43 aggregates suggest that GRP75 is probably still actively involved in fostering neuronal protein quality control (PQC) mechanisms, counteracting incipient aggregation, whereas GRP75 is probably no longer active in protecting against the large globular or skein-like inclusions because of decline in overall PQC mechanisms. Thus, the observed early compensatory responses mediated by GRP75 may become targets of toxic inclusions, as we discovered that even in healthy iMNs, PolyGA expression likely impaired GRP75 function at the MAM, leading to deficits in mitochondrial Ca^2+^ uptake. This is likely to be detrimental to neuronal survival in the long run due to inefficient ATP generation, and thus compromised bioenergetics.

The precise mechanism leading to PolyGA-mediated sequestration of GRP75 remains unclear. GRP75 belongs to the HSP70 family of chaperones based on sequence homology. It is the only member of this protein family mostly localized and functioning within mitochondria [18]. Therefore, we also investigated the physical localization of PolyGA within MAMs and were unable to detect PolyGA biochemically in the MAM fractions (data not shown). Recently, UBQLN2 was implicated in recognizing HSP70 ubiquitination, which facilitated UBQLN2-HSP70-GA complex formation and promoted PolyGA degradation [66], thus, further studies are required to decipher whether ubiquitination is involved in sequestering GRP75.

Several previous studies have also shown the deleterious effects of DPRs other than PolyGA such as PolyGR or PolyPR in mediating ER–mitochondrial toxicity [12, 42, 58]. Interestingly, in this regard, our data show specific reduction in GRP75 expression in association with PolyGA expression, but not other DPRs. Considering our findings of reduction of GRP75 expression in human *C9ORF72* neurons, PolyGA may actively participate in mitochondrial dysfunction via sequestration of GRP75 in *C9ORF72-*ALS/FTD. Although this requires further study, it is possible that after translation, GRP75 is transported primarily into the mitochondria, and either inefficient mitochondrial translocation complex or insufficient ATP required for the transport might make GRP75 vulnerable to cytoplasmic sequestration by PolyGA. Alternatively, PolyGA might sequester GRP75 within the cytosol, thereby hindering its proper localization to the mitochondria and, thus diminishing its association at the MAMs.

We found that transient overexpression of GRP75 restored mitochondrial function by normalizing complex I and IV and reduced ER stress in *C9-500* spinal MNs. As the ER lacks an internal ATP generation machinery, it crucially relies on the mitochondrial ATP supply, and any mitochondrial ATP deficits coupled with aggregation-prone proteins might negatively influence ER homeostasis. Most likely, ER homeostasis critically depends on a permanent supply of ATP, which is essential for optimal protein folding [24] and for the clearance of aggregated proteins in proteinopathies, such as NDs [21, 27]. Because GRP75 enhances ER–mitochondria associations, it should be involved in alleviating ATP deficits, thus promoting the dissipation of adverse effects of ER stress. Notably, overexpression of GRP75 decreased levels of large PolyGA inclusions. These data are highly indicative of an interplay between GRP75 and PolyGA. Nevertheless, this relationship requires further study, as does the mechanism by which GRP75 attenuates ER stress and PolyGA aggregation. In addition, increased expression and co-localization of GRP75 with pTDP-43 might be indicative of GRP75 trying to manage the PQC pathways in those MNs, while decreased expression of GRP75 could be indicative of the failure of such PQC mechanism organized by GRP75. It is also reasonable to speculate that GRP75, a molecular chaperone of the HSP70 family, might also be involved in TDP-43 phase separation, which requires ATPase-dependent activity of HSP70 chaperones [65].

Whether exogenous overexpression of GRP75 rescued disease pathology by primarily restoring ER homeostasis, thereby reducing PolyGA aggregation, or whether restoring mitochondrial function was also a key factor in mitigating PolyGA toxicity, remains to be determined. Taken together, our work indicates that GRP75 serves as an early compensatory response in *C9ORF72*-ALS/FTD, and its pathological targeting by PolyGA aggregates not only disrupts this adaptive response involving ER–mitochondrial cross talk, but also affects mitochondrial function and neuronal survival.

## Supplementary Information

Below is the link to the electronic supplementary material.

Supplementary file1 (DOCX 14438 KB)
