# Supplementary material for: PolyGA targets the ER stress-adaptive response by impairing GRP75 function at the MAM in C9ORF72-ALS/FTD
Source: Acta Neuropathol. 2022 Sep 19;144(5):939–66. doi: 10.1007/s00401-022-02494-5 (PMC9547809; doi:10.1007/s00401-022-02494-5)
Supplement: Supplementary file 1 — Supplementary file1 (DOCX 14438 KB) [file 401_2022_2494_MOESM1_ESM.docx]

**PolyGA targets the ER stress-adaptive response by impairing GRP75 function at the MAM in C9ORF72-ALS/FTD**

**Supplementary Information**

**METHODS**

Laser dissection microscopy (LDM) on spinal MNs

*WT* mice were injected with 0.1µg/g of tunicamycin (Sigma, T7765) or saline. Mice were anesthetized and isolated spinal cords were embedded in O.C.T compound and frozen on dry ice. 18 μm thick sections were cut and transferred onto a membrane slide (Membrane Slide 1.0 PET, ZEISS). Slides were stained with 0.1% Toluidine blue in PBS, washed with 70% and 100% EtOH, and dried at room temperature (RT). 2500-3000 lumbar MNs were collected using the PALM MicroBeam system (ZEISS), and transferred into capture tube caps (AdhesiveCap 500 opaque, ZEISS), and stored at -80 until RNA extraction. qPCR was performed with GoTaq® 2-Step RT-qPCR System (Promega), using a Rotor-Gene Q (Qiagen). The experiments were repeated 2-3 times, qPCR was run thrice for each gene. The *gapdh* gene was used for normalization and the delta Ct ((ΔCt) threshold cycle) was determined for each gene relative to *gapdh.*

Primary neuron culture

Primary cortical neurons were prepared from P0 *C9-500* BAC neonates and cultured as previously described [4]. Briefly, cortex was isolated, cleaned in Hank’s Balanced Salt solution (HBSS) and dissociated enzymatically (papain) at 37^o^C for 30 min, followed by gentle trituration in triturating media (MEM, 30% glucose, 50mM L-glutamin and 10% Horse serum). Primary cortical neurons were resuspended in adhesion medium (MEM, 30% glucose, 50mM L-glutamine, Pen/strep and 10% Horse serum), plated on poly-D-lysine coated plates. After 3 hours (h), adhesion media was replaced with growth media (Neurobasal media, B27, 50mM L-glutamine and Pen/strep) and kept in an incubator at 37°C with 5% CO2.

Lentivirus preparation and viral transduction of iMNs and cortical neurons

Lentivirus for PolyGA_149_-GFP, PolyPR_175_-GFP, PolyGR_149_-GFP and GFP were prepared using previously published protocol from Riguet et al. [6] and Barde et al [2]. Neurons were transduced with lentivirus particles after 7 days of differentiation. Mouse Hspa9 cDNA ORF clone (Origene, MR209980), was subcloned into AAV vector and viral particles were generated at Virovek (Hayward, CA, USA). AAV6-GRP75 viral stock was diluted in cell culture media to achieve the multiplicity of infection of 5000. Half of the culture media was substituted with virus-containing media after 7 days of iMN differentiation. Virus-containing media was replaced with fresh media after 24h and iMNs were cultured for additional 7 days.

Pharmacological treatments of iMNs

Matured iMNs were treated with Tunicamycin (1 µg/ml) for 18-20h, 10 µM MKT-077 for 1h 45 min (Sigma-Aldrich, M5449) or 15 µM Salubrinal (Enzo, ALX-270-428) for 48h before further analysis.

Quantitative real-time PCR of iPSCs, iMNs and dNeus

RNA was extracted using SV Total RNA isolation system (Promega, Z3100). cDNA was made using GoScriptTM Reverse transcriptase (A5000, Promega). qPCR was performed with HOT FIREPol^®^ EvaGreen^®^ qPCR Mix Plus (Rox) (Solis Biodyne, 08-24-00008), using a Applied Biosystem^TM^ 7500 Real-Time PCR systems. RNA levels were normalized to *Gapdh* and gene expression differences were quantified with the comparative CT method (Supplementary table 1, online resource for primer sequences).

Proximity ligation assay (PLA) on iMNs and mouse spinal cord.

PLA was done on iMNs using Duolink® In Situ Starter Kit Mouse/Rabbit according to manufacturer’s protocol. iMNs were fixed with 4% PFA and permeabilized with 0.1% Triton X in PBS. Cells were blocked using 40µl of Duolink® Blocking solution for 1h at 37°C using a humid chamber. Cells were incubated overnight at 4°C with primary antibodies diluted in the Duolink® antibody diluent. Cells were washed with buffer A, and incubated for 1h at 37°C with the PLUS and MINUS PLA probes ((Probe Anti-mouse MINUS, DUO92004 and Probe anti-rabbit PLUS, DUO92002) diluted 1:5 in the Duolink® antibody diluent. Coverslips were washed with 1x wash buffer A, and incubated with ligation solution for 30 min at 37°C. Cells were rewashed with 1x wash buffer A, incubated with amplification solution for 100 min at 37°C. Cells were washed twice with 1x wash buffer B and once with 0.01x wash buffer B, and coverslips were mounted onto glass slides using Duolink® In Situ mounting medium with DAPI. Images were acquired using FluoViewTM FV1000 (Olympus). For mouse spinal cord, PLA was adapted from Gomes et al. [3]. Briefly, 50 μm free-floating spinal cord sections were mounted on double frosted slide (Huberlab) and air-dried. Slides were rinsed in 0.01% Triton X-100 in PBS for 12 min followed by 3 washes of 5 min in PBS, and placed in a humid chamber with blocking solution for 1h at 37°C. Subsequently slides were incubated with the following primary antibodies (mouse anti-VDAC1, Millipore, MABN504; rabbit anti-IP_3_R, ABCAM, ab5804; mouse anti-myc tag, Cell Signaling Technologies, 9B11; mouse anti-GRP75, ABCAM, ab53098; rabbit anti-GRP75, ABCAM, ab227215) in a humid chamber for 2 nights at 4°C. Slides were rinsed thrice for 5 min in buffer A, and incubated with PLA probes at a working concentration of 1:10 for 1h at 37°C. Ligation was performed at 37°C for 45 min followed by 3 washes of 5 min in buffer A. Amplification was performed at 37°C in a dark humid chamber for 100 min. Sections were rinsed twice for 10 min in buffer B, followed by a third wash in 0.001% buffer B. Slides were dried and coverslips were mounted with Duolink In Situ Mounting Medium with DAPI, and edges were sealed with nail polish and stored at -20°C overnight before confocal imaging using FluoView 1000 (Olympus).

Measurement of mitochondrial activity from iMNs and rodent spinal cord

Mitochondrial respiration was monitored via oxygen consumption rate (OCR), using Seahorse XFp instrument. iMNs and iPSCs were plated on Seahorse plates pre-coated with Matrigel. Culture media was changed with Seahorse DMEM basal media 45 min before the measurements. Seahorse ports were filled with 1 µM oligomycin, 1 µM FCCP, 0.5 µM of rotenone and antimycin A for iMNs and 1.5 µM oligomycin, 0.25 µM FCCP, 0.5 µM of rotenone and antimycin A for iPSCs. OCR in the different respiration states was accessed with Wave 2.3.0 software (Agilent). Mice were transcardially perfused with PBS and spinal cord was isolated, washed with cold mitochondrial isolation buffer (D-Mannitol 210 mM; sucrose 70 mM, HEPES 5 mM, EGTA 1mM, fatty acid-free BSA 0.5%; pH7.2), and homogenized in a Potter-tube [8]. The homogenate was centrifuged at 800 x g for 10 min at 4°C; the supernatant collected and centrifuged at 8000 x g for 10 min at 4°C. After two washes in mitochondrial isolation buffer, the pellet was resuspended in mitochondria assay solution (D-Mannitol 220 mM; sucrose 70 mM, KH2PO4 10 mM, MgCl2 5 mM, HEPES 2 mM, EGTA 1mM, fatty acid-free BSA 0.2%; pH7.2), containing Malate 5 mM and Glutamate 10 mM for assessing complex 1 driven respiration. Protein concentration of mitochondria suspensions were determined using BioRad assay and a suspension volume of 5μg/well was loaded on Seahorse culture plates in triplicate wells per animal; and simultaneous mitochondrial OCR was measured for both *WT* and *C9-500* conditions. Seahorse Flux Pak cartridges were filled with the reagents at 10-fold concentration: (A) ADP: 40 mM; (B) oligomycin: 25ug/mL; (C) FCCP: 40 μM; (D) antimycin: 40 μM.

Colorimetric staining of mitochondria complexes

Mice were rapidly perfused with PBS, isolated spinal cord was embedded in O.C.T compound and quickly frozen on dry ice. 20 µm thick sections were cut with a cryostat and transferred onto an adhesive glass slide. Sections were incubated for 30 min at 37° C in freshly prepared appropriate complex histochemistry media: (1) Complex I: 1.23mg/ml (1.5mM) Nitroblue tetrazolium (NBT, Sigma) and 0.625 mg/ml NADH (Sigma) were mixed in PBS, pH=7.4. Complex IV: 0.5mg/ml 3,3’-diaminobenzidine tetrahydrochloride (DAB, Sigma), 1mg/ml cytochrome c (Sigma) and approximately 2μg/ml (a few crystals) bovine catalase (Sigma) were mixed in PBS, pH=7.4. Subsequent to the staining of each complex, sections were washed 3×10 min in PBS and dehydrated for 4 min in 70% EtOH, 4min in 90% EtOH, 10 min in 100% EtOH, 10 min in Xylol and mounted with Eukitt. Images were acquired using BX51 Olympus microscope.

Mitochondrial Ca^2+^ uptake measurements

Mitochondrial Ca^2+^ uptake measurements were adapted from McKenzie et al. [5]. Briefly, neurons were incubated for 45 min in staining solution: 156mM NaCl, 3mM KCl, 2mM MgSO4, 1.25mM KH2PO4, 10mM D-glucose, 2mM CaCl2 and 10mM HEPES pH 7.35, 5 µg/ml (w/v) Fluo-4, AM, 10 µM Verapamil. After a brief wash with Ca^2+^ free HBSS, cells were incubated for 10 min with intracellular solution: 6mM NaCl, 130mM KCl, 7.8mM MgCl2, 1mM KH2PO4, 0.4mM CaCl2, 2mM EGTA, 10mM HEDTA, 2mM malate, 2mM glutamate, 2mM ADP, 20mM HEPES pH 7.1, 25 µg/ml (w/v) digitonin and 1 µM thapsigargin. Images were acquired every 20 seconds using 40x water immersion objective fitted to Fluoview 1000 (Olympus), the first 60 seconds considered as baseline, followed by depolarization of neuron with 50mM KCl. iMNs were treated with 5 µM of MKT-077 for 1h, prior to 45 min incubation in staining solution containing 5 µM MKT-077. Images were analyzed using Fiji, multiple region of interest (ROI) were chosen inside the cytosol and fluorescence intensity was calculated over different time frames. To calculate ΔF, the median intensity values were divided by the average of the first 60 seconds of recording (F_0_) per single ROI.

Electron microscopy

Anesthetized mice were transcardially perfused with 0.1 M PBS (pH=7.4), followed by fixation solution (2.5% glutaraldehyde (GA) +2% PFA in 0.1 M Na-cacodylate, pH=7.4). Spinal cord was isolated and post-fixed in fixation solution. iMNs were grown on 24 well plates without coverslip, cell media was removed and iMNs were fixed with 5 % glutaraldehyde. Bloc staining, dehydration and embedding were performed as follows: post-fixation was done in 0.15 M cacodylate buffer, 1.5% potassium ferrocyanide and 2% osmium tetroxide. The samples were incubated with 0.64 M pyrogallol for 15 min at RT and for 5 min in a water bath at 50°C, and rinsed with water. Samples were incubated in 2% OsO4 for 22 min at RT and 8 min in a water bath at 50°C. After water rinses, the samples were incubated overnight in a solution of 0.15 M gadolinium acetate (LFG Distribution, Lyon, France) and 0.15 M samarium acetate (LFG Distribution) pH 7.0. Followed by water rinses, samples were incubated in 1% Walton's lead aspartate [7], useful for ultra-structural enzymology [7] at 60°C for 30 min. After staining, the samples were dehydrated for 5 min in graded EtOH (20%, 50%, 70%, 90%, 100%, 100%) at 4°C. The blocks were infiltrated with Durcupan resin mixed with EtOH at ratios of 1:3 (v/v), 1:1, and 3:1, each step lasted for 2h, to finally be infiltrated with pure Durcupan overnight. The samples were transferred to fresh Durcupan and the resin was polymerized for 3 days at 60°C. Pyramids with a surface of approximately 500 × 500μm2 were trimmed with a razor blade. The blocs were imaged at the serial block-face scanning electron microscopy (SBF-SEM) using a Quanta FEG 250 (FEI, Eindhoven, The Netherlands), equipped with a 3View2XP in-situ ultramicrotome (Gatan). Images were acquired in low or high vacuum accordingly to block quality. Acceleration voltage of 3 kV and pixel dwell time was set between 2 and 2.5 µs with a pixel size from 6 to 24 nm. Fiji software was employed to analyze the mitochondria-ER contacts and sphericity for 3 view EM images. The mitochondria sphericity was calculated using different ROI per SBF-SEM stack; values were plotted as frequency distribution (%). ER-mitochondria contact length was measured following the mitochondria in contact with the ER across the SBF-SEM stack.

MAM isolation

MAM isolation was performed according to Annunziata et al. 2013 [1]. Briefly, animals were decapitated, brain extracted, halved, placed on ice, and weighed. The brain was homogenized with 15 strokes using a dounce homogenizer containing 1ml cold Solution A (0.32M Sucrose, 1mM NaHCO_3_, 1mM MgCl_2_, 0.5mM CaCl_2_, Halt Protease and Phosphatase inhibitor cocktail). Homogenates were diluted up to 10 volumes w/v, and centrifuged at 1400g for 10 min at 4°C. The supernatant was transferred to a falcon tube and the pellet was resuspended in 10 volumes of Solution A and homogenized with 4 strokes and transferred to a new falcon tube and centrifuged at 710 g for 10 min at 4°C. The supernatant was pooled with the previous supernatant and centrifuged at 13800g for 10 min at 4°C. The pellet was resuspended in 10 volumes of Solution A and homogenized with 4 strokes and centrifuged at 13800g for 10 min at 4°C. The supernatant was pooled with the previous supernatants. The pellet was resuspended in 10 volumes of Solution A and homogenized with 4 strokes, and centrifuged at 13800g for 10 min at 4°C. The pellet was resuspended in 4.8 ml/g of original brain weight of Solution B (0.32M sucrose, 1mM NaHCO_3_, Halt Protease and Phosphatase Inhibitor Cocktail) and homogenized with 6 strokes. Using glass Pasteur pipettes, a discontinuous sucrose gradient was prepared in an Ultra-Clear Beckman centrifuge tube, consisting of 3 layers (bottom to top): 1.2M sucrose in 1mM NaHCO_3_, 1M Sucrose in 1mM NaHCO_3_, 0.85M Sucrose in 1mM NaHCO3, and the resuspended pellet. The tubes were centrifuged at 82500g for 2 hours at 4°C. The pellet, corresponding to the crude mitochondria, was resuspended in 2ml isolation medium (250mM Mannitol, 5mM HEPES pH 7.4, 0.5mM EGTA, 0.1% BSA, Halt protease and phosphatase inhibitor cocktail). The mitochondrial suspension was layered upon a 30% Percoll (GE Healthcare) gradient in gradient buffer (225mM Mannitol, 25mM HEPES pH 7.5, 1mM EGTA, 0.1% BSA) in an Ultra-Clear Beckman centrifuge tube on ice. The sample was centrifuged at 95000g for 30 min at 4°C. The upper band corresponding to the light fraction was removed with a Pasteur pipette, diluted in 10ml isolation medium, and centrifuged at 6300g for 10 min at 4°C. Additional isolation medium was added to the supernatant and transferred to an Ultra-Clear Beckman centrifuge tube, centrifuged at 100,000g for 1hr at 4°C to obtain the MAM fraction (pellet). The pooled supernatants were transferred to an Ultra-Clear Beckmann centrifuge tube and centrifuged at 100,000g for 1 hour at 4°C to obtain the cytosolic fractions (supernatant) and ER fractions (pellet).

Immunoblotting

Cells were lysed using either NP-40 or RIPA lysis buffer, containing Halt Protease and Phosphatase Inhibitor Cocktail (ThermoFisher Scientific). The Pierce BCA protein assay (ThermoFisher Scientific) was used to determine protein concentrations. Protein samples were boiled at 90°C for 5 min with Laemmli buffer containing β-mercaptoethanol, separated on a SDS-PAGE gel and transferred to a PVDF membrane. Membranes were blocked in 5% milk dissolved in PBS containing 0.1% Tween for 1 hour. Primary antibodies used were: mouse anti-GRP75 (Abcam, ab2799, 1:1000), rabbit anti-GRP75 (Abcam, ab53098, 1:1000), mouse anti-GAPDH (Acris, ACR001P, 1:10000), rabbit anti-mitofusin-2 (Abcam, ab124773, 1:1000), mouse anti-Calnexin (Proteintech, 66903-1-Ig, 1:2000). Membranes were incubated with primary antibodies overnight at 4°C, washed with PBS and 0.1% Tween, followed by incubation for 1h at 20°C with secondary Alexa Fluor 680 goat anti-rabbit and Alexa Fluor 800 goat anti-mouse secondary antibodies, and developed using Li-Cor Odyssey infrared imaging system.

PolyGA antibody production

4GAD Mouse monoclonal antibodies directed against the N-terminal sequence of the *C9ORF72* poly(GA) protein were generated by three intraperitoneal (IP) injections at two weeks interval of two months-old BALB/c female mice with a MELRSRALGAC peptide conjugated to KLH plus 200 µg of poly(I/C) as adjuvant. Spleen cells were fused with Sp2/0.Agl4 myeloma cells and supernatants of hybridoma cultures were tested at day 10 by ELISA for cross-reaction with the MELRSRALGAC peptides. Positive supernatants were tested by immunofluorescence and western blot on PolyGA-GFP transfected HEK293 cells. Specific cultures were cloned twice on soft agar. Specific hybridomas were established and ascites fluid was prepared by injection of 2x106 hybridoma cells into Freund adjuvant-primed BALB/c mice. All animal experimental procedures were performed according to European authority guidelines.

Measurement of muscle coordination and survival

Rotarod apparatus (Ugo Basile, Comerio, Italy) was used to assess general motor performance during the light phase of the 12h light/12h dark cycle. For the inverse rotarod, the rod accelerated from 15 to 33 rpm in 10 seconds, 33 rpm to 15 rpm in 10 seconds, followed by inversing the direction of the rod and repeating the same procedure. One trial lasted maximum 110 seconds, with 3min rest in between trials. Three trials every 10 days was performed for *C9-500* animals and respective controls. Mice were habituated on the rotating rod at fixed speed for 3 days prior to the baseline recording. All measurements were done blind for the genotype of mice. The hanging wire test was done by placing the mice on top of a cage cover and once the animal was stable, the cage cover was gently inverted, and the latency of the first fall was recorded. The average number of falls was assessed within 120 seconds. To measure longevity, the mice were followed until they became immobile and required food and water inside the cage.

**Supplementary References**

1. Annunziata I, Patterson A, d’Azzo A (2013) Mitochondria-associated ER Membranes (MAMs) and Glycosphingolipid Enriched Microdomains (GEMs): Isolation from Mouse Brain. Journal of Visualized Experiments. doi: 10.3791/50215

2. Barde I, Salmon P, Trono D (2010) Production and Titration of Lentiviral Vectors. In: Current Protocols in Neuroscience. John Wiley & Sons, Inc., Hoboken, NJ, USA

3. Gomes I, Sierra S, Devi LA (2016) Detection of Receptor Heteromerization Using In Situ Proximity Ligation Assay. Curr Protoc Pharmacol 75:2.16.1-2.16.31. doi: 10.1002/cpph.15

4. Mahul-Mellier A-LL, Burtscher J, Maharjan N, Weerens L, Croisier M, Kuttler F, Leleu M, Knott GW, Lashuel HA (2020) The process of Lewy body formation, rather than simply α-synuclein fibrillization, is one of the major drivers of neurodegeneration. Proceedings of the National Academy of Sciences 117:4971 LP – 4982. doi: 10.1073/pnas.1913904117

5. McKenzie M-, Lim SC-, Duchen MR (2017) Simultaneous Measurement of Mitochondrial Calcium and Mitochondrial Membrane Potential in Live Cells by Fluorescent Microscopy. JoVE e55166. doi: doi:10.3791/55166

6. Riguet N, Mahul-Mellier A-L, Maharjan N, Burtscher J, Croisier M, Knott G, Hastings J, Patin A, Reiterer V, Farhan H, Nasarov S, Lashuel HA (2021) Nuclear and cytoplasmic huntingtin inclusions exhibit distinct biochemical composition, interactome and ultrastructural properties. Nat Commun 12:6579. doi: 10.1038/s41467-021-26684-z

7. Walton J (1979) Lead asparate, an en bloc contrast stain particularly useful for ultrastructural enzymology. Journal of Histochemistry & Cytochemistry 27:1337–1342. doi: 10.1177/27.10.512319

8. Wettmarshausen J, Perocchi F (2017) Isolation of Functional Mitochondria from Cultured Cells and Mouse Tissues. pp 15–32

**Supplementary Figures**


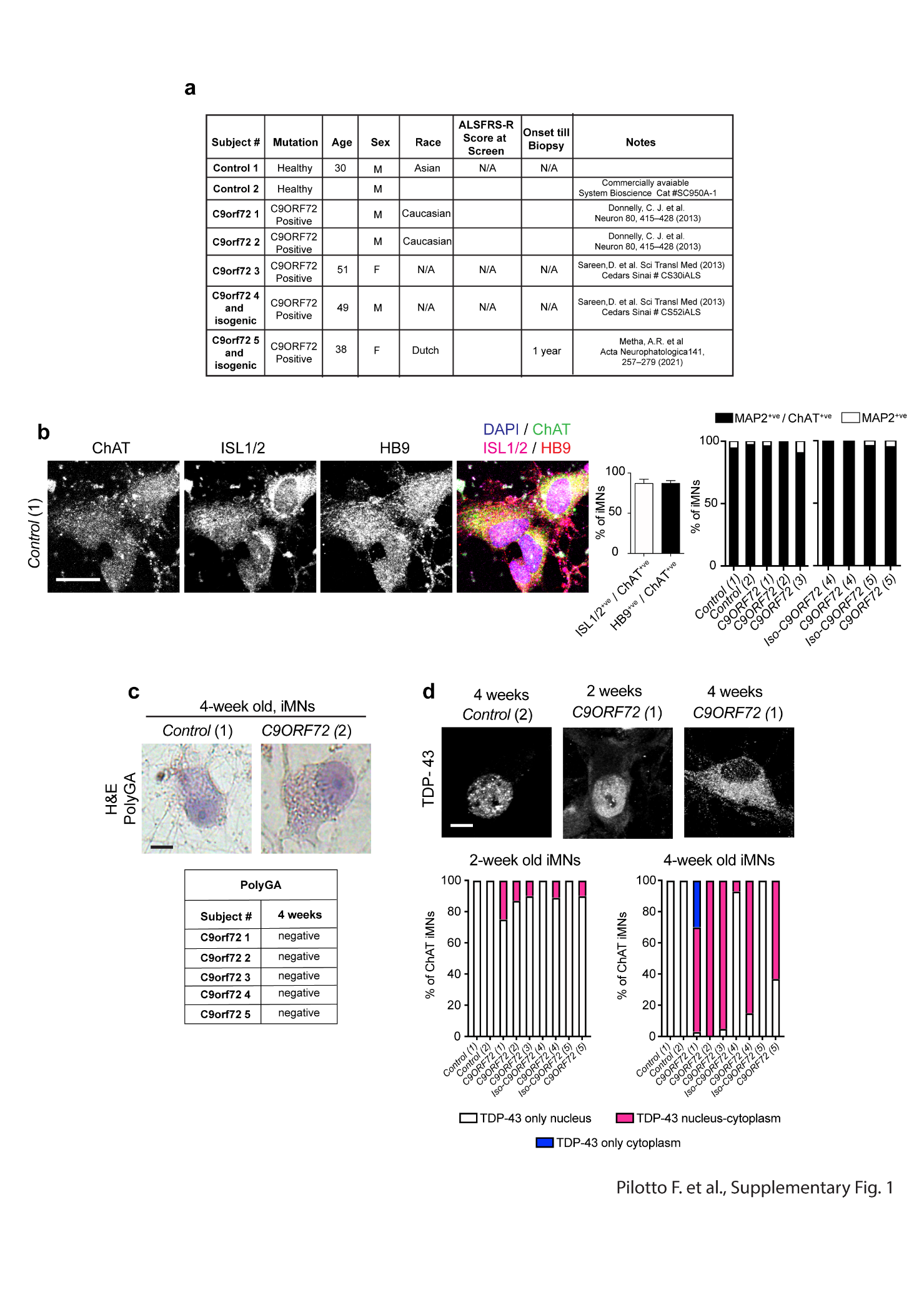


**Supplementary Figure 1: Characterization of IPSC-derived MNs (iMNs)**

**(a)** Table with demographic information about iPSC lines from *C9ORF72*-ALS/FTD patients and healthy controls (*Ctrls*) employed to generate iMNs. **(b)** Representative images of iMNs stained for MN markers (ChAT, ISL1/2, and HB9). 90% to 100% double positive (MAP2 +ve and ChAT +ve) iMNs from all lines were used in this study. Multiple independent conversion of iPSCs to iMNs were used. **(c)** Representative PolyGA immunohistochemical images counterstained with H&E for *Ctrl(1)* and *C9(2)* patient line. Note; PolyGA aggregates were not detected in *C9ORF72* patient-derived iMNs until four weeks in culture. **(d)** Representative images of TDP-43 immunostaining *Ctrl(2)* and *C9(1)* patient line at 2 and 4 weeks showing mislocalization of TDP-43 and the appearance of cytosolic TDP-43 at 4 weeks. **Bottom:** characterization of TDP-43 appearance, note at 2 weeks; patient lines do not present any ALS-related pathology. Scale bars (b): 10 µm, (c & d) 25 µm.


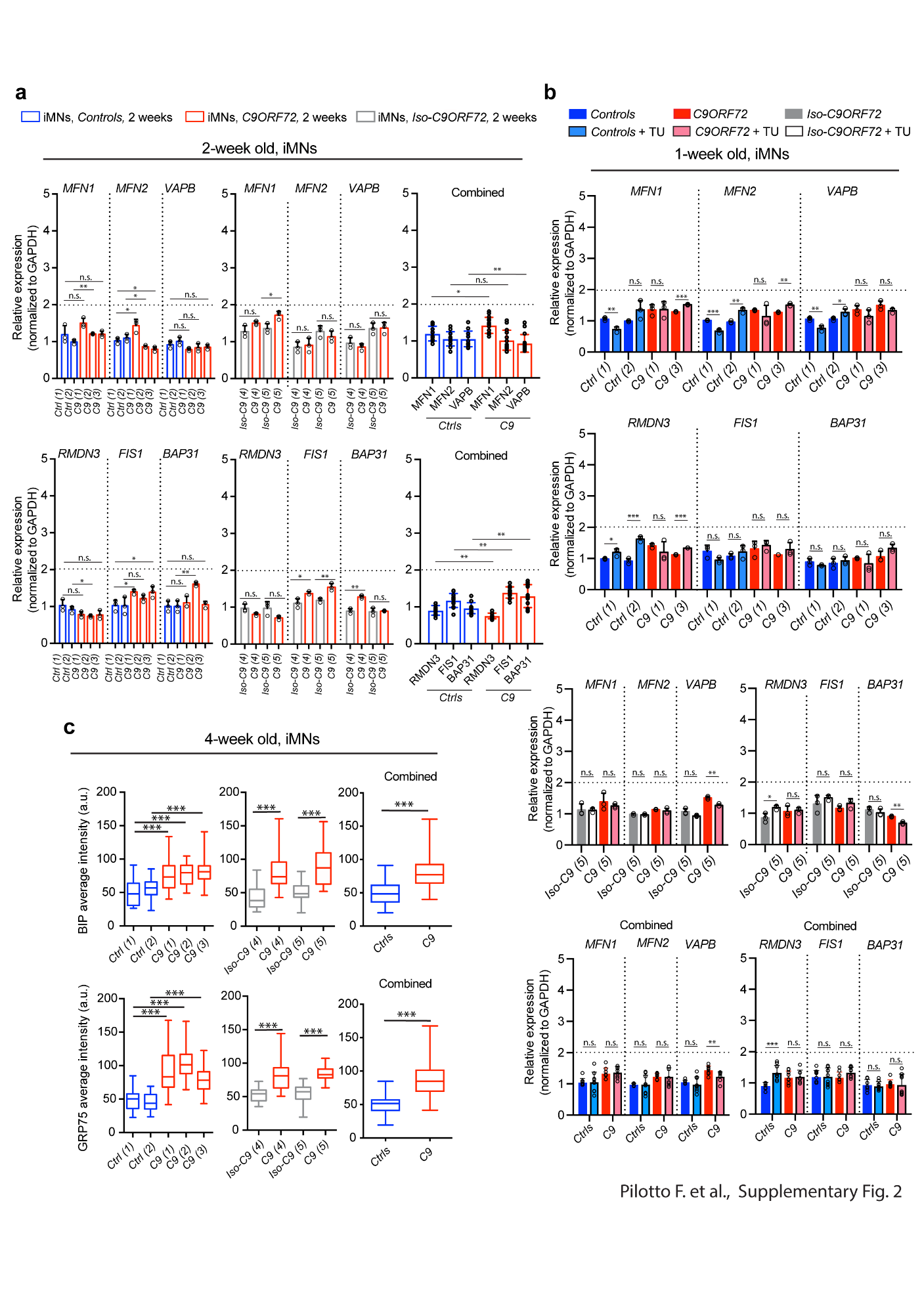


**Supplementary Figure 2: ER stress does not influence mRNA levels of other MAM molecules**

**(a)** qPCR analysis of mitochondria associated membrane (MAM) molecules from 2-week old iMNs. All *C9ORF72* patient lines display mild changes (below 2 folds) in MAM tethers. Combined graph represents the average values for *Ctrls* lines (*Ctrl(1-2)), Iso-C9(4-5)*) and *C9(1-2-3-4-5)*, Unpaired t-test; *MFN1*: *Ctrls* vs *C9* t=2.242, P= 0.0465*; *MFN2*: *Ctrls* vs *C9* t=0.6129, P= 0.0465, n.s.; *VAPB*: *Ctrls* vs *C9* t=3.842, P= 0.0027**; *RMDN3*: *Ctrls* vs *C9* t=3.681, P= 0.0011**; *FIS1*: *Ctrls* vs *C9* t=3.279, P= 0.0031**; *BAP31*: *Ctrls* vs *C9* t=3.339, P= 0.0026**. **(b)** 1-week old iMNs treated with TU (1µg/ml) for 18 hours do not display any significant increase (more than 2-fold) in transcripts levels of MAM molecules: *MFN1, MFN2 VAPB, RMDN3, FIS1, BAP31*. Unpaired t-test; *MFN1*: *Ctrls* vs *Ctrls +* TU t=0.04295, P= 0.9663, n.s.; *MFN1*: *C9* vs *C9* + TU t=0.4829, P= 0.6357, n.s.; *MFN2*: *Ctrls* vs *Ctrls +* TU t=0.06354, P= 0.9501, n.s.; *MFN2*: *C9* vs *C9* + TU t=0.02517, P= 0.9802, n.s.; *VAPB*: *Ctrls* vs *Ctrls +* TU t=0.9164, P= 0.3731, n.s.; *VAPB*: *C9* vs *C9* + TU t=3.218, P= 0.0054**; *RMDN3*: *Ctrls* vs *Ctrls +* TU t=4.914, P= 0.0002***; *RMDN3*: *C9* vs *C9* + TU t=0.1638, P= 0.8719, n.s.; *FIS1*: *Ctrls* vs *Ctrls +* TU t=0.1326, P= 0.8962, n.s.; *FIS1*: *C9* vs *C9* + TU t=1.923, P= 0.0724, n.s.; *BAP31*: *Ctrls* vs *Ctrls +* TU t=0.5712, P= 0.5758, n.s.; *BAP31*: *C9* vs *C9* + TU t=0.3287, P= 0.7466, n.s. **(c)** Quantitative Analysis (Q.A.) of GRP75 and BiP expression reveals significantly increased levels of GRP75 and BiP in all *C9ORF72* lines when compared to controls or isogenic controls at 4 weeks post differentiation. Unpaired t-test BiP: *Ctrl(1)* vs *C9(1)* t=4.661, P<0.0001***; *Ctrl(1)* vs *C9(2)* t=4.967, P<0.0001***; *Ctrl(2)* vs *C9(3)* t=4.983, P<0.0001***; *Iso-C9(4)* vs *C9(4)* t=6.094, P<0.0001***; *Iso-C9(5)* vs *C9(5)* t=5.595, P<0.0001***; Unpaired t-test GRP75: *Ctrl(1)* vs *C9(1)* t=6.816, P<0.0001***; *Ctrl(1)* vs *C9(2)* t=10.63, P<0.0001***; *Ctrl(2)* vs *C9(3)* t=7.201, P<0.0001***; *Iso-C9(4)* vs *C9(4)* t=5.966, P<0.0001***; *Iso-C9(5)* vs *C9(5)* t=8.465, P<0.0001***. Combined graph for *Ctrl* lines (*Ctrl 1-2, Iso-C9 4-5*) and *C9* (*C9 1-2-3-4-5*), Unpaired t-test; *BiP*: *Ctrl* vs *C9* lines at 4 weeks, t=10.91, P<0.0001***. *GRP75 Ctrl* vs *C9lines at* 4 weeks, t=14.14, P<0.0001***.


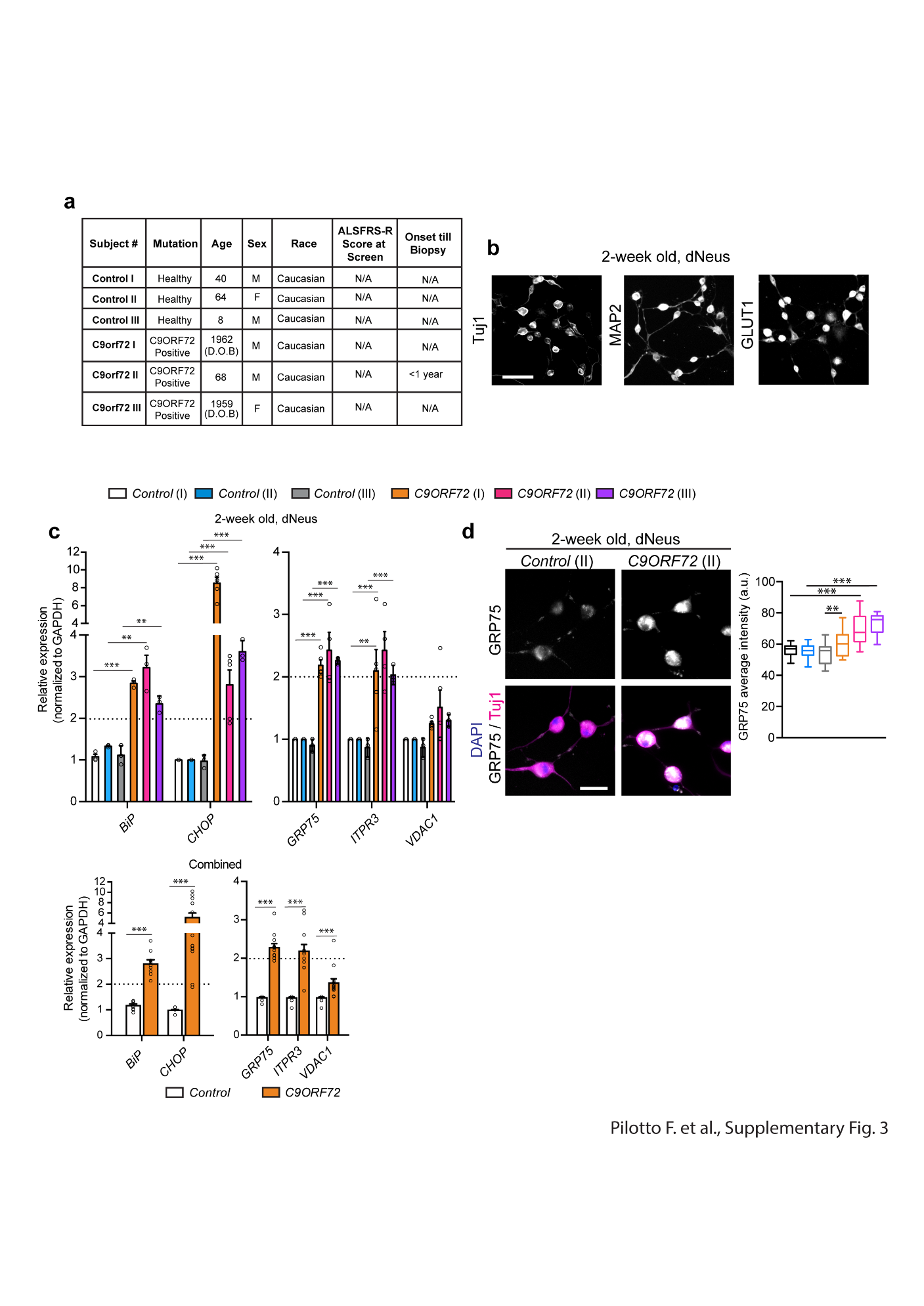


**Supplementary Figure 3: Neuronal characterization of fibroblast-derived neurons (dNeus)**

**(a)** Table with the phenotypic information about *C9ORF72* patients and healthy controls. **(b)** Representative images of 2-week old dNeus stained with neuronal markers Tuj1, MAP2 and GLUT1, corresponding to glucose uptake. **(c)** qPCR from three *Ctrl* (I,II & III) and three *C9ORF72* patient fibroblasts directly differentiated to neurons (dNeus). *C9ORF72* lines show more than two fold increase in ER stress markers *BiP* and *CHOP*, and ensuing upregulation of GRP75 transcripts. Combined graph for *Ctrl* lines (*Ctrl I,II & III*) and *C9* (*C9 I,II & III*), Unpaired t-test; *BiP*: *Ctrls* vs *C9* t=9.671, P<0.0001***; *CHOP*: *Ctrls* vs *C9* t=5.533, P<0.0001***; *GRP75*: *Ctrls* vs *C9* t=14.53, P<0.0001***; *ITP3R*: *Ctrls* vs *C9* t=7.939, P<0.0001***; *VDAC1*: *Ctrls* vs *C9* t=3.791, P=0.0008***. **(d)** Representative images of GRP75 staining of 2-week old dNeus from *Ctrl(II)* and *C9ORF72(II).* Q.A. present significantly increased GRP75 expression in *C9*-patient lines *I, II and III*. Unpaired t-test: *Ctrl(III)* n=18 vs *C9ORF72(I)* n=24, t=2.838**, *Ctrl(I)* n=14 vs *C9ORF72(II)* n= 10, t=4.459***, *Ctrl(II)* n=14 vs *C9ORF72(III)* n=9. t=7.556***). Scale bar: (b) 150 µm, (d) 50 µm.


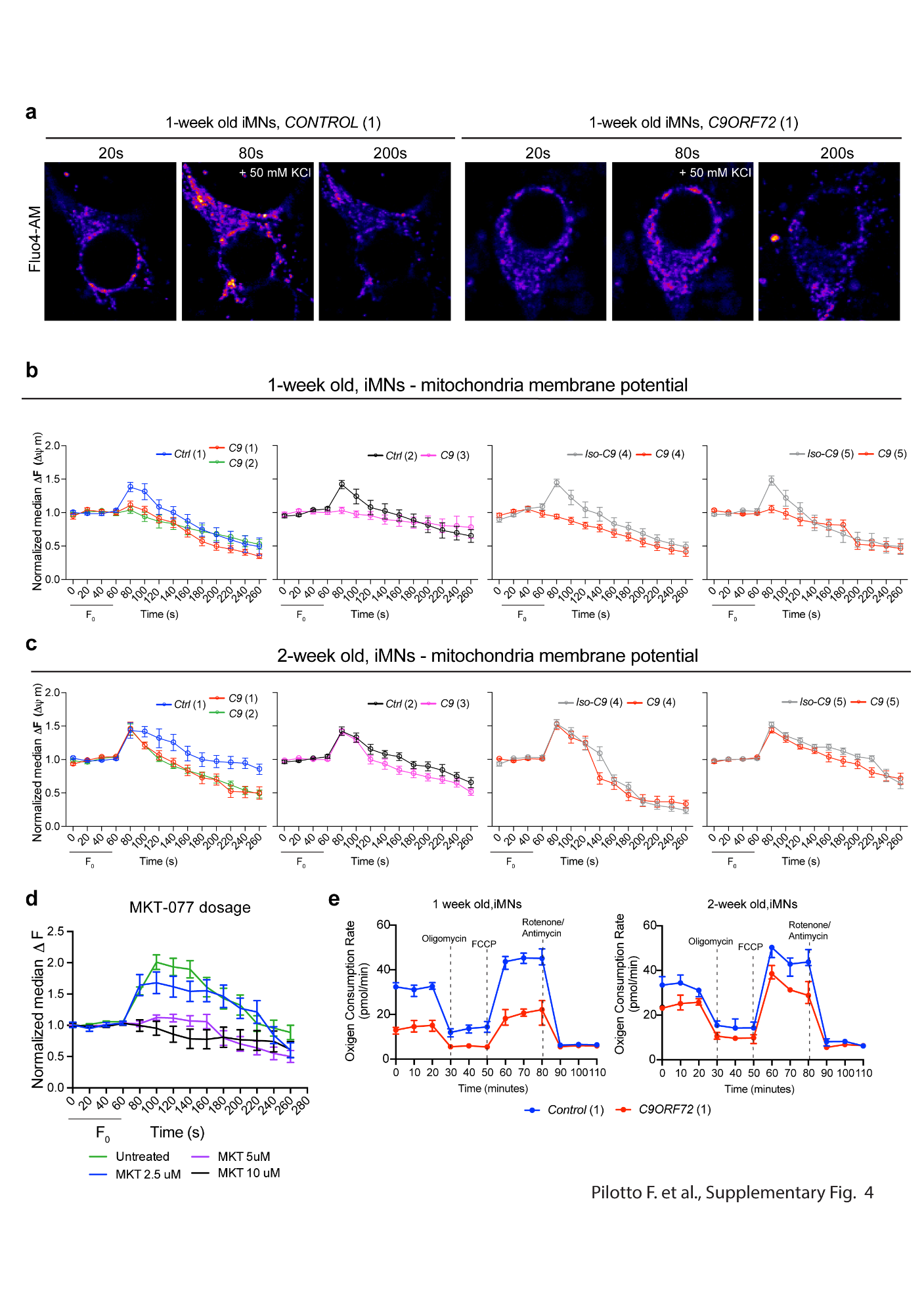


**Supplementary Figure 4: Impaired mitochondrial membrane potential and function in mutant iMNs**

**(a**) Representative images of Ca^2+^ transients using Fluo4-AM indicator for *Ctrl(1)* and *C9(1)* iMNs at 1-week of differentiation. Note the increase in fluorescence in control iMNs when neurons are depolarized with KCl. **(b-c)** Traces of mitochondrial membrane potential from *Ctrl* or isogenic and *C9ORF72* iMNs, reveals deficits in mitochondrial membrane potential within *C9ORF72* iMNs at 1 week but not at 2 weeks post differentiation. **(d)** Dosage curves for Ca^2+^ uptake after MKT-077 treatment of iMNs, validating that 5 uM treatment is sufficient to abolish mitochondrial Ca^2+^ transients. **(e)** Representative traces of mitochondrial respiration from *Ctrl(1)* and *C9(1)* iMNs, showing mitochondrial functional deficits in *C9ORF72* patient line at one week, but those deficits are normalized at 2 weeks, when GRP75 expression is elevated.


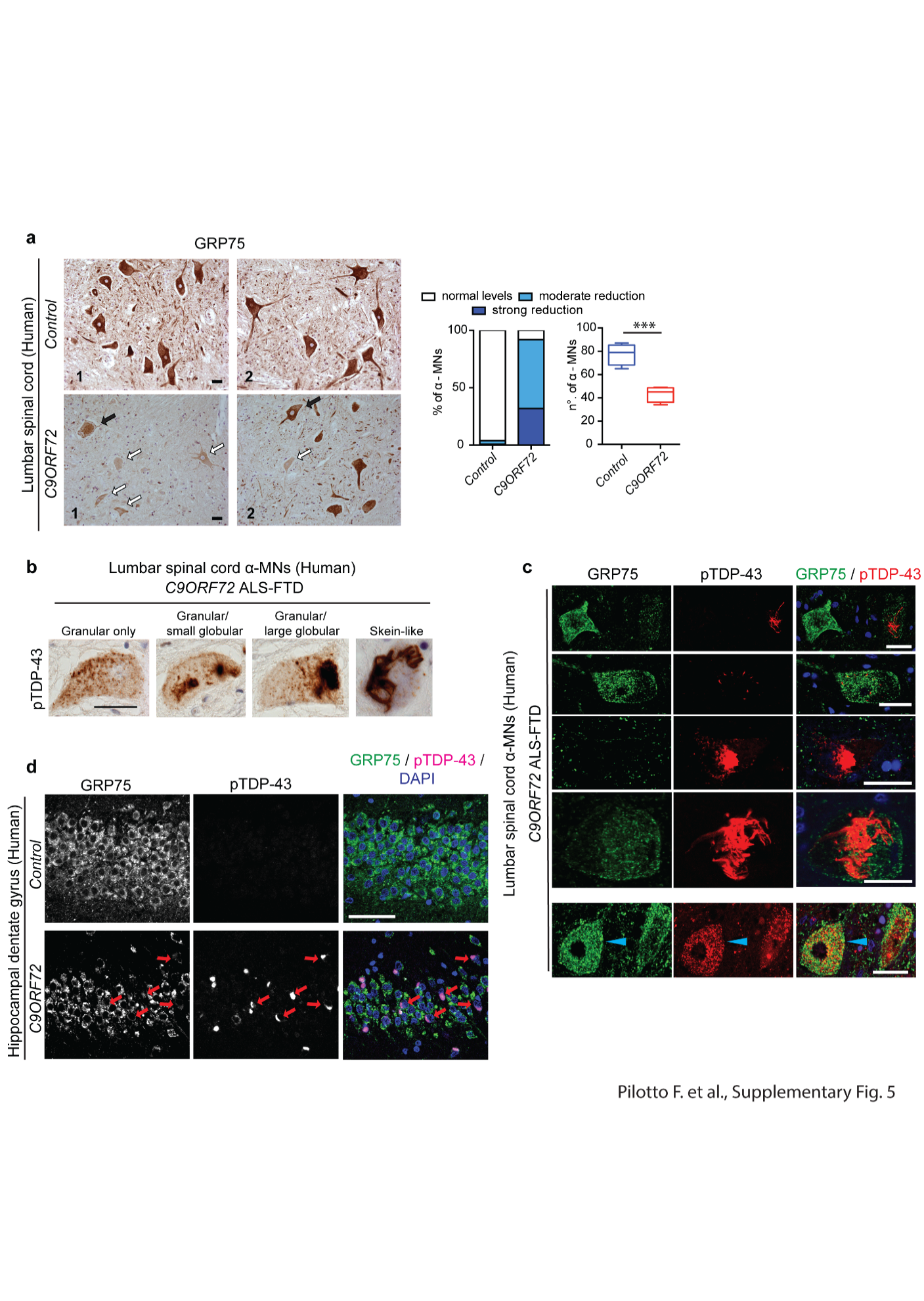


**Supplementary Figure 5: *C9ORF72*-ALS/FTD postmortem human CNS tissue exhibits varying levels of GRP75 immunoreactivity, coinciding with aggregates size and form**

**(a)** Representative GRP75 DAB immunohistochemistry, showing markedly (white arrows) and moderately reduced (black arrows) labeling of many neurons in human lumbar spinal cord anterior horns of *C9ORF72* ALS/FTD cases compared to the strong, uniform staining of large and small neurons in the normal *Controls* (upper panel). Images are shown from two different *C9ORF72*-ALS/FTD and control samples. Q.A. of MN numbers and GRP75 immunoreactivity: normal levels (*C9ORF72:* 8%), moderate reduction (*C9ORF72:* 60%) and strong reduction (*C9ORF72:* 32%). (Number of MNs: *Control*: 77.5 ± 4.6; *C9ORF72*: 43.25 ± 3.33). Three sections each of n=4 *C9ORF72*-ALS/FTD patients and n=4 age-matched controls. **(b)** Patterns of pTDP-43 aggregation (dash- or dot-like/granular, dense/globular, dense/skein-like) in lumbar spinal cord α-MNs of *C9ORF72*-ALS/FTD patients. **(c)** Representative double immunofluorescence labelling of GRP75 and pTDP-43 in lumbar spinal cord α-MNs of *C9ORF72*-ALS/FTD patients. Neurons with high levels of GRP75 show only minor, if any pTDP-43 aggregation, and vice versa. Occasionally increased levels of GRP75 were also associated with a buildup of small granular pTDP-43 aggregates (blue arrowheads, lower panel). **(d)** Representative triple co-immunolabeling showing an overall reduction of GRP75 immunoreactivity in hippocampal dentate gyrus neurons and markedly reduced levels of GRP75 in pTDP-43 aggregate-bearing dentate gyrus neurons (red arrows) in a representative *C9ORF72* ALS patient. Scale bars: (a) 20 µm, (b & c) 50 µm, (d) 15 µm.


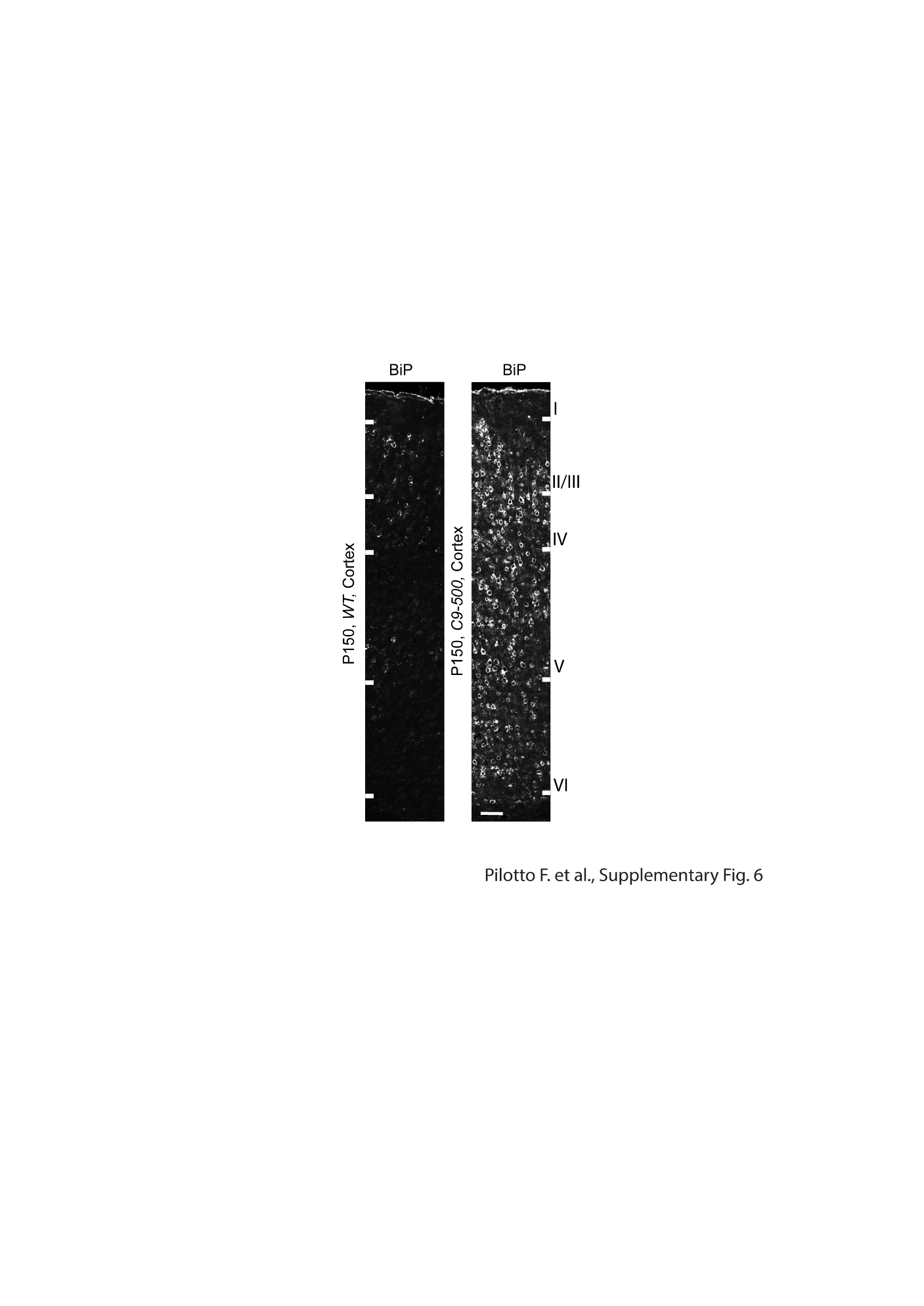


**Supplementary Figure 6: Presence of ER stress in *C9-500* motor cortex**

Representative images of primary motor cortex from *WT* and *C9-500* animals showing increased levels of BiP throughout the cortical layers, suggestive of ER stress. Scale bar: 200µm.


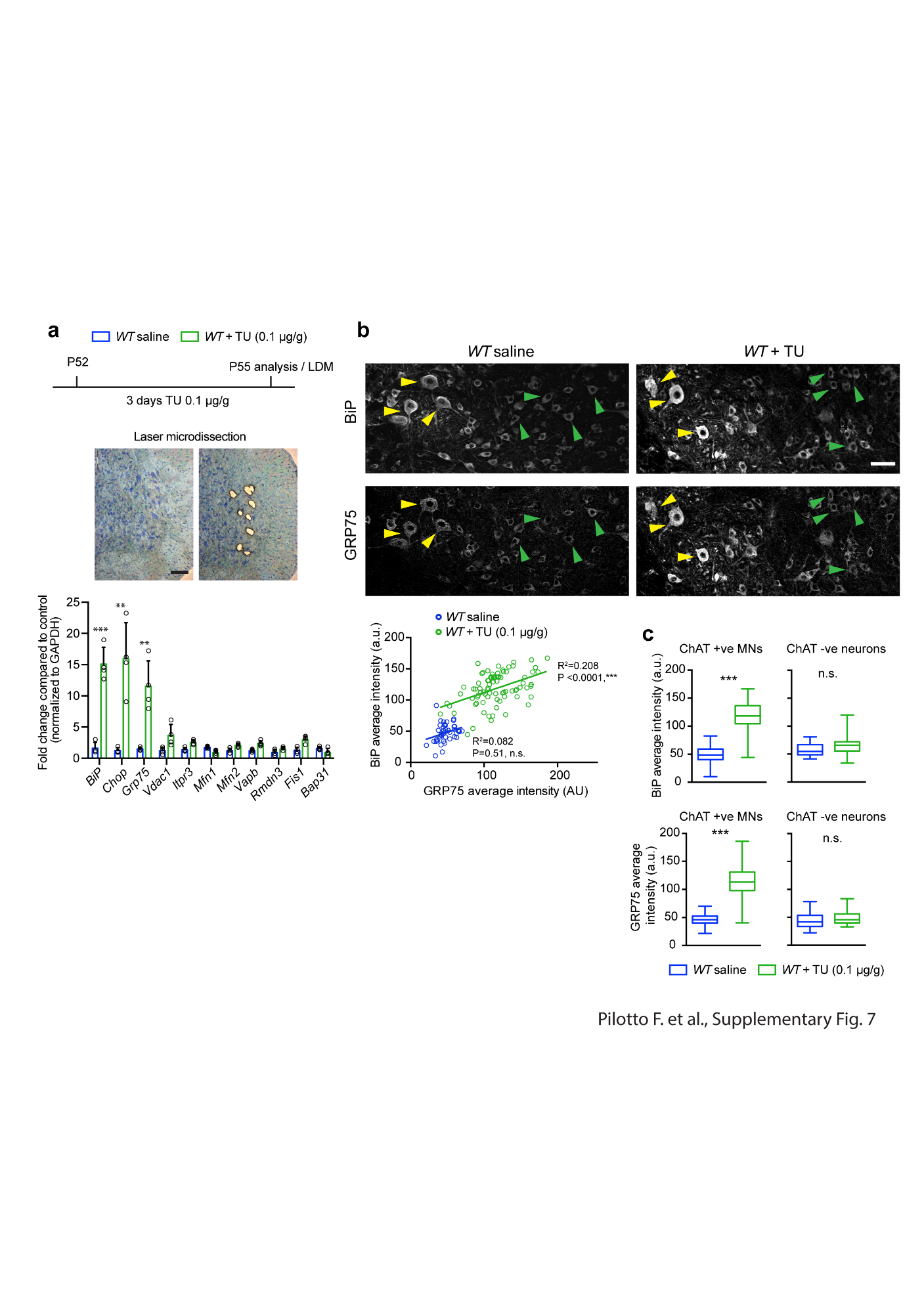


**Supplementary Figure 7: *WT* MNs robustly upregulate GRP75 in response to ER stress**

**(a)** Experimental timeline for *in-vivo* tunicamycin (TU) treatment to induce ER stress. Representative images of a spinal cord section before and after laser dissection microscopy (LDM) of the spinal MNs. **Bottom:** qPCR of MNs collected with LDM, showing more than 10-fold increase in mRNA levels of *BiP*, *Chop* and *Grp75* after TU treatment, but not in other MAM resident molecules (unpaired t-test *BiP*: *WT* saline vs *WT*+TU t=9.390, P<0.0001***, *Chop WT* saline vs *WT*+TU t= 5.50, P= 0.0023**, *Grp75 WT* saline vs *WT*+TU t=4.943, P=0.0026**). n=3 *WT* saline and 3 *WT* + TU. **(b)** Representative images of MNs from *WT* mice treated with saline or TU, displaying increased protein expression of BiP and GRP75 in ChAT+ve MNs but not in ChAT-ve MNs. **Bottom:** Direct correlation between BiP expression levels and GRP75 in *WT* animals treated with TU, (*WT* saline Y=0.3939*X+28.77, P=0.0504, n.s.; *WT* +TU Y=0.3975*X+72.22, P<0.0001***). **(c)** Q.A. of BiP and GRP75 average intensity in ChAT+ve MNs and ChAT-ve neurons, (unpaired t-test: BiP in ChAT+ve MNs: *WT* saline n=47 vs *WT*+TU n=82 t=17.18, P<0.0001***; BiP in ChAT-ve neurons: *WT* saline n=29 vs *WT*+TU n=37 t=1.778, P=0.0537 n.s.; GRP75 in ChAT +ve MNs: *WT* saline n=48 vs *WT*+TU n=82 t=15.60,P<0.0001***; GRP75 in ChAT-ve neurons: *WT* saline n=29 vs WT+TU n=37 t=1.397, P=0.1672 n.s.). Scale bars: (a) 150 µm, (b) 50 µm.


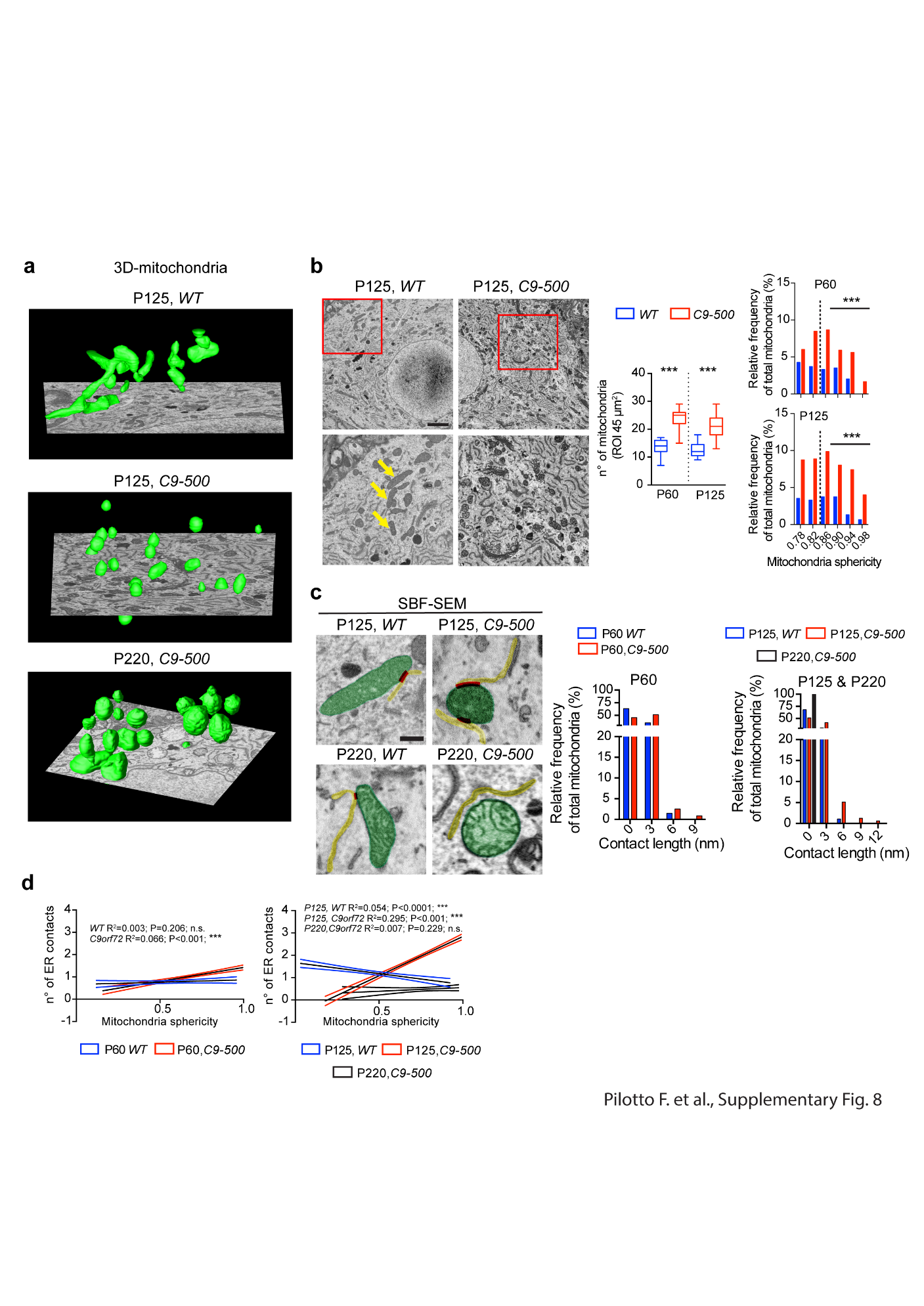


**Supplementary Figure 8: Presence of spherical mitochondria having increased contact with ER membranes in *C9-500* spinal cord, reflecting elevated GRP75 expression**

**(a)** 3D segmentation of mitochondria from serial block-face scanning electron microscopy (SBF-SEM) images of P125, *WT*, P125 and P220, *C9-500*. Note the appearance of spherical and progressively fragmented mitochondria in *C9-500* MNs. **(b)** A 2D representative image from a SBF-SEM indicates that mitochondria within mutant MNs are primarily vesicular as compared to *WT* MNs, where elongated mitochondria are largely present (yellow arrows). Note the lack of yellow arrows in mutant condition in the zoomed region of interest (ROI). **Right:** Q.A. of mitochondria numbers within MNs performed by counting consecutive images through the stack of SBF-SEM images (100 to 150 images/per stack/genotype), indicating a significant increase in mitochondria numbers in *C9-500* MNs (P60 *WT* mean ± SEM 13.54± 0.4479 vs P60 *C9-500* mean ± SEM 23.97 ± 0.5492, Unpaired t test P<0.0001***, P125 *WT* mean ± SEM 12.36± 0.4434 vs P125 *C9-500* mean ± SEM *21.08* ± 0.6906, Unpaired t test P<0.0001***). Sphericity analysis performed on SBF-SEM image stacks plotting sphericity values as relative frequency distribution histogram. Note the higher percentage of spherical mitochondria in mutant MNs (P60 *WT* bin center 0.86=3.717, 0.90=3.532, 0.94=2.045, 0.98=0; P60 *C9-500* bin center 0.86=8.696, 0.90=5.929, 0.94=5.632, 0.98=1.680; P125 *WT* bin center 0.86=3.761, 0.90=3.761, 0.94=1.327, 0.98=0.664, Unpaired t test, mitochondria numbers P60 *WT,* n=538 vs P60 *C9-500,* n=1012, t=9.094, df=1548***P<0.0001. P125 *C9-500* bin center 0.86=9.854, 0.90=8.029, 0.94=7.421, 0.98=4.015, Unpaired t test, mitochondria numbers P125 *WT,* n=452 vs P125 *C9-500* n=822, t=16.24, df=1272, P<0.0001 ***; 3 animals/genotype/age). **(c)** Representative images of 2D examples of ER-mitochondria contacts, the mitochondria are marked in green and the ER in yellow, red line represents the surface in contact between the two organelles. **Right**: histogram representing relative frequency distribution of the contact length between ER and mitochondria reveals larger contact area within mutant condition, when GRP75 is upregulated between P60- P125 and reduced ER-mitochondria contacts length at P220 when GRP75 is downregulated (bin center: *WT* P60: 0=63.235, 3=35.294, 6=1.471,9=0; *C9-500* P60: 0=45.378, 3=51.261, 6=2.521, 9=0.840; *WT* P125: 0=68.132, 3=30.769, 6=1.099, 9=0, 12=0; *C9-500* P125: 0=51.282, 3=41.667, 6=5.128, 9=1.282, 12=0.641; *C9-500* P220: 0=100). **(d)** Linear regression between the no. of contacts per mitochondria and their sphericity at P60, P125 and P220. (P60 *WT:* Y=0.2121*X+0.6565, P=0.206, P60 *C9-500;* Y=1.245*X+0.1785, P<0.0001, P125 *WT;* Y=0.9732*X+1.638, P<0.0001, P125 *C9-500*; Y=3.514*X+0.6767, P<0.0001, P220 *C9-500*; Y=0.33*X+0.22, P=0.229). n=3 animals/genotype/age. Scale bars: (b) 5 µm, (c) 300 nm.


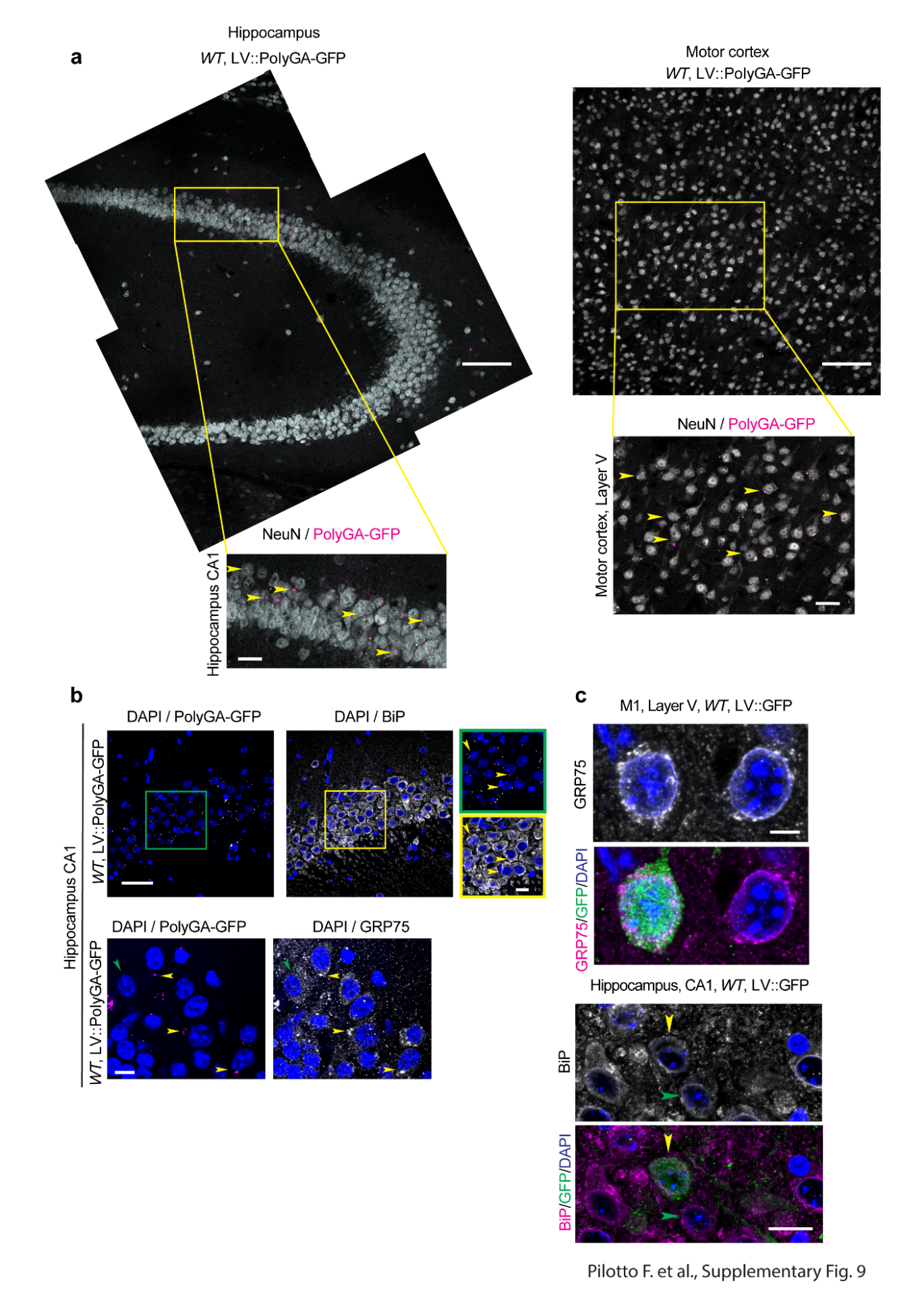


**Supplementary Figure 9: Wide spread CNS transduction of LV::PolyGA-GFP in adult mice**

**(a)** Representative images of hippocampus and motor cortex from LV::PolyGA-GFP injected *WT* mice. **(b)** Representative confocal images of hippocampus from *WT* LV::PolyGA-GFP injected mice, showing increased expression of BiP, indicative of ER stress (yellow arrows). **Bottom:** Higher magnification images showing GRP75 sequestered with PolyGA inclusions (yellow arrows). Green arrow points to uninfected neuron, showing cytoplasmic GRP75. **(c)** Representative images from *WT* LV::GFP injected animals. Note no change in BiP or GRP75 expression levels is observed between GFP positive neurons (yellow arrow in the hippocampus image) and neighboring uninfected (green arrow in the hippocampus image) both in the cortex and in the hippocampus. Scale bars: (a) 200 µm (zoom 50 µm), (b) 50 µm (zoom 10 µm) & 20 µm (c) 5 & 10 µm.


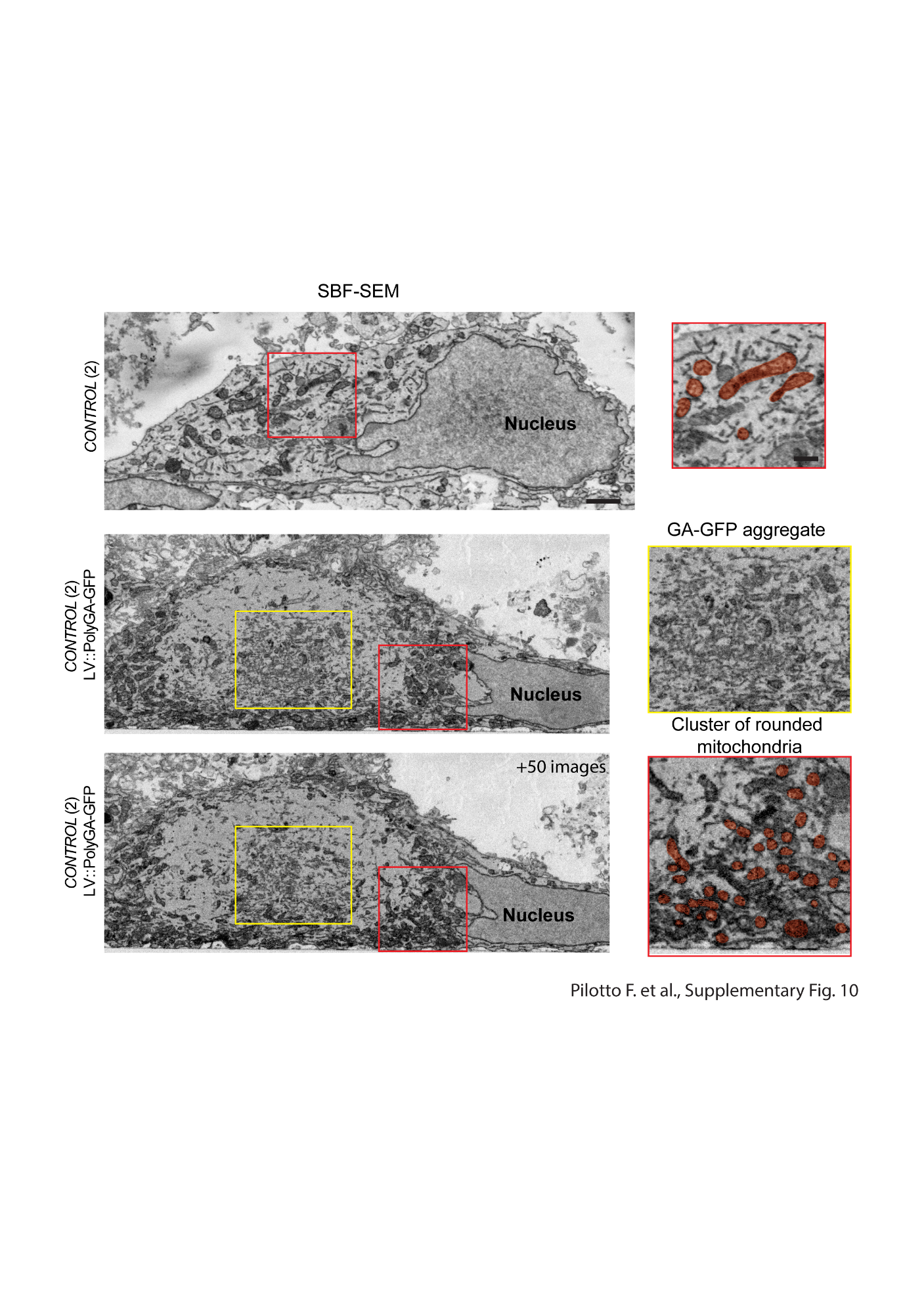


**Supplementary Fig. 10: PolyGA aggregates alter mitochondrial localization in iMNs**

Representative images from SBF-SEM of 3-week old iMN (*Ctrl(2)*) naïve and transduced with LV::PolyGA-GFP. Images reveal that PolyGA transduced iMN is largely devoid of mitochondria and ER, especially within the region whether the aggregate (yellow box) is present. Notably, mitochondria (red box) display small and rounded morphology (filled in red) and are found at the periphery near the nucleus of iMN. Scale bars: (a) 3 µm (zoom 0.5 µm).


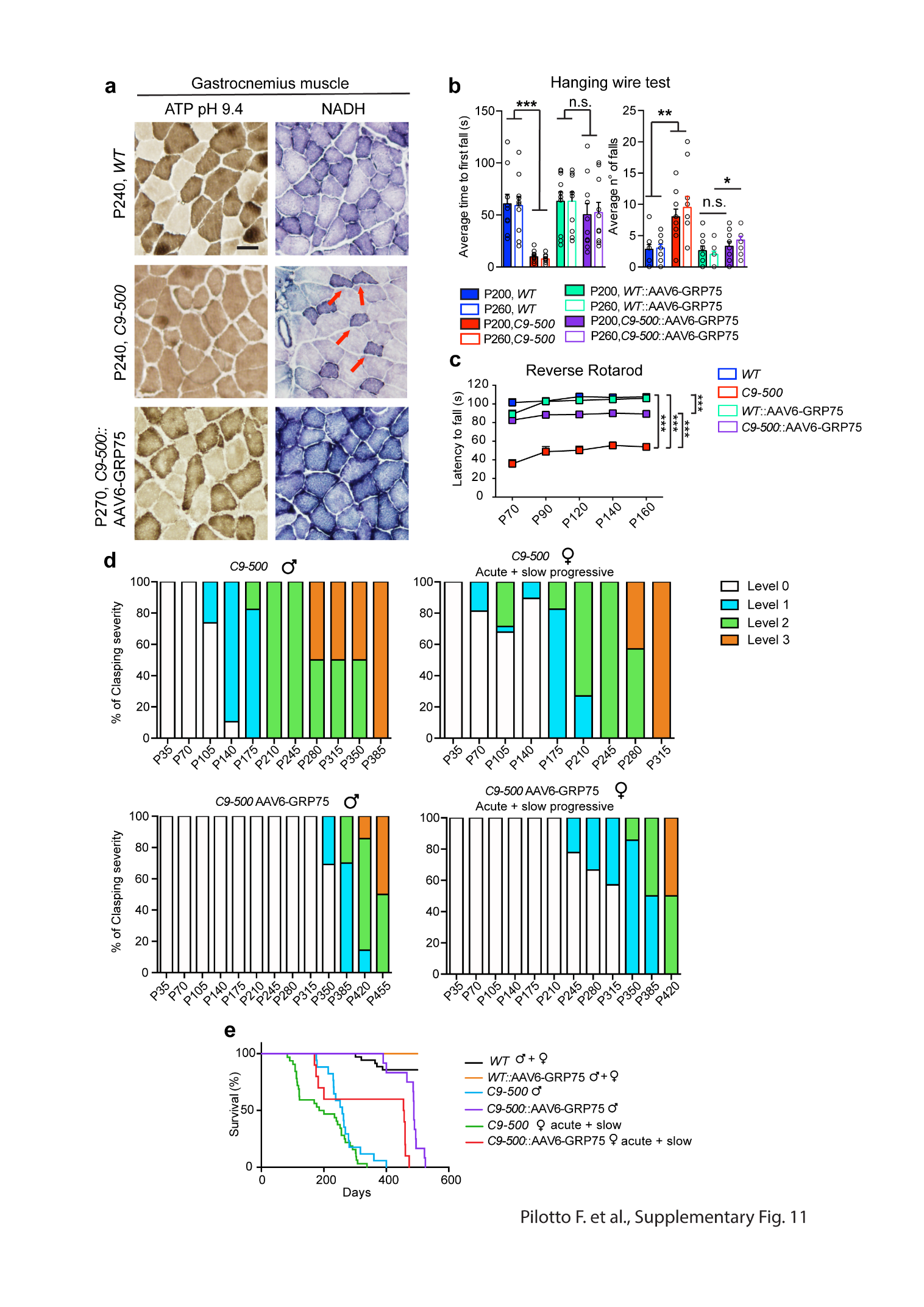


**Supplementary Figure 11: GRP75 overexpression rescues muscle and motor pathology in *C9-500* mice**

**(a)** Representative images of muscle sections with staining for ATP pH 9.4 and NADH from P240, *C9-500* and *WT* and P270 *C9-500* mice overexpressing GRP75. Visible checkerboard pattern in *WT* muscle, while in *C9-500* muscle, loss of the checkerboard pattern, lacking pale Type 1 fibers and dark Type 2 fibers. Note, the intensely NADH stained fibers mainly being atrophic and/or angulated in *C9-500* muscle. The GRP75 overexpressing muscle presents a modified checkerboard pattern with incomplete fiber grouping, in both staining, suggestive of ongoing regeneration. n=3 mice/age/genotype. **(b)** Hanging wire test done at P200 and P260 presents improved performance in *C9-500*::AAV6-GRP75 mice compared to age matched controls (10 mice/genotype), **Left**: average time to first fall, **Right**: average number of falls, both measurements are comparable to *WT* in *C9-500*::AAV6-GRP75**.** Unpaired t-test average time to first fall: P200, *WT* vs *C9-500* p<0.0001***; P260, *WT* vs *C9-500* p<0.0001***; P200, *WT*::AAV6-GRP75 vs *C9-500*::AAV6-GRP75 p=0.3679, n.s.; P260, *WT*::AAV6-GRP75 vs *C9-500*::AAV6-GRP75 p=0.4182, n.s.; Unpaired t-test average number of falls: P200, *WT* vs *C9-500* p=0.0023**; P260, *WT* vs *C9-500* p=0.0029**; P200, *WT*::AAV6-GRP75 vs *C9-500*::AAV6-GRP75 p=0.5161, n.s.; P260, *WT*::AAV6-GRP75 vs *C9-500*::AAV6-GRP75 p=0.0103*. **(c)** Reverse rotarod performance graph plotted in seconds as latency to fall. Values are the means and SEM (No. of animals: 10 *WT*; 10 *C9-500;* 11 *WT*::AAV6-GRP75; 20 *C9-500*::AAV6-GRP75). Note the improved performance of *C9-500* mice overexpressing AAV6-GRP75. Two-way ANOVA: interaction P=n.s., F (12,235) =0.9449, age P<0.0001. F (4,235) =11.20, treatment P<0.0001 F (3,235) =321.3, Bonferroni post hoc *WT* vs *C9-500****; *WT* vs *WT*::AAV6-GRP75 n.s; *WT* vs *C9-500*::AAV6-GRP75***; *C9-500* vs *C9-500*::AAV6-GRP75***; *WT*::AAV6-GRP75*** vs *C9-500*::AAV6-GRP75***. **(d)** Progression of the hindlimb clasping phenotype in *C9-500* animals: males (n=19), females (n=32) and *C9-500*::AAV6-GRP75 animals: males (n=18 males) and females (n=13 females). Note the delay in the appearance of higher clasping scores in GRP75 overexpressing *C9-500* cohort. AAV6-GRP75 overexpression ameliorates the pathological phenotype in *C9-500* mice. **(e)** Survival curve: No. of mice: *C9-500*::AAV6-GRP75=22 (12 males, 6 females, 4 acute females), *C9-500=*51 (19 males, 19 females, 13 acute females). Median survival: *C9-500* females acute and slow progressive: 192.5 days, *C9-500* slow progressive males: 259 days, *C9-500*::AAV6-GRP75 slow progressive males: 488 days and females: 456 days. Log-rank (Mantel-Cox) test: P< 0.0001***. Scale bars: (a) 150 µm.


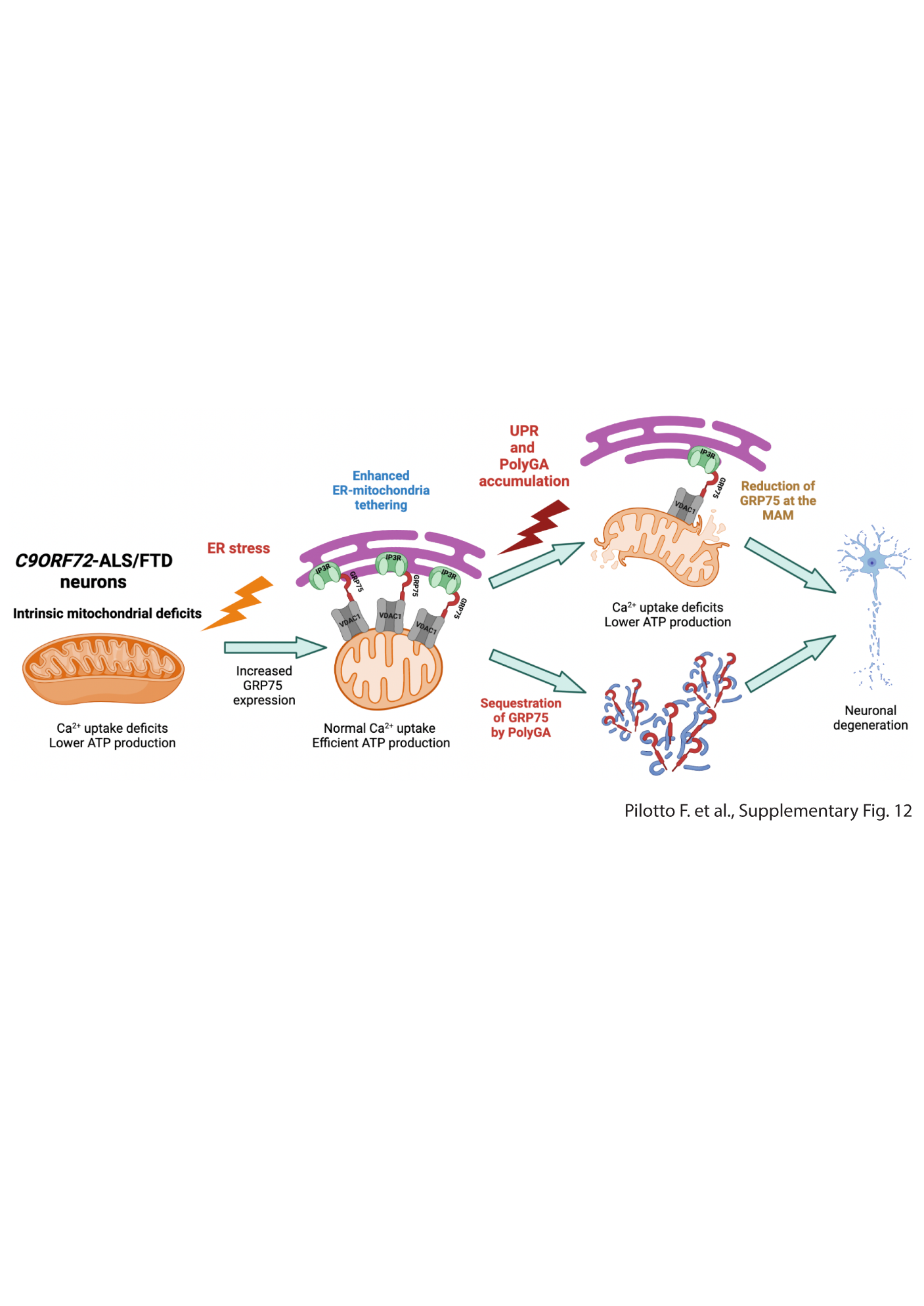


**Supplementary Figure 12: Scheme portraying the interplay between ER stress, GRP75 expression and selectively enhanced ER-mitochondria association in promoting endogenous neuroprotective mechanism in *C9ORF72*-ALS/FTD**

The proposed model depicts the presence of early intrinsic mitochondrial deficits in *C9ORF72* MNs, reflected by the impaired Ca^2+^ uptake and reduced ATP production. The progressive appearance of ER stress response, which induces GRP75 upregulation, thus selectively enhancing ER-mitochondrial tethering, normalizing mitochondrial function and confers neuroprotection in *C9ORF72*-ALS/FTD. The progression of ER stress into UPR coincides with the deposition of large PolyGA aggregates, leading to diminished GRP75 expression and its sequestration within PolyGA aggregates, thus, resulting in impaired ER-mitochondria coupling, mitochondrial dysfunction and eventually MN degeneration.

| **Mouse primer sequences** | | |
| --- | --- | --- |
| BiP | Forward | TGTCTTCTCAGCATCAAGCAAGG |
|  | Reverse | CCAACACTTCCTGGACAGGCTT |
|  |  |  |
| Chop | Forward | GGAGGTCCTGTCCTCAGATGAA |
|  | Reverse | GCTCCTCTGTCAGCCAAGCTAG |
|  |  |  |
| Derl1 | Forward | GTATTCTACTCGGCTTGAAGCAG |
|  | Reverse | CCAGACGTAGAGCACTGACATG |
|  |  |  |
| Calr | Forward | AAAGGACCCTGATGCTGCCAAG |
|  | Reverse | TCAGGGATGTGCTCTGGCTTGT |
|  |  |  |
| Sil1 | Forward | CAGGAGAAGAGTGCGAAGGTAC |
|  | Reverse | GGGATGAATCCTGGGTCAACTC |
|  |  |  |
| Calx | Forward | GTGGTGCCTATGTGAAGCTGCT |
|  | Reverse | GCAGTTTGTAGTCCTCTCCACAC |
|  |  |  |
| Vdac1 | Forward | AGTGACCCAGAGCAACTTCGCA |
|  | Reverse | CAGGCGAGATTGACAGCAGTCT |
|  |  |  |
| Grp75 | Forward | GTTGGTATGCCAGCAAAACGGC |
|  | Reverse | CAAGCATCACCATTGGAGGCAC |
|  |  |  |
| itp3r | Forward | GCAACCACATCTGGACGCTCTT |
|  | Reverse | AGAAGGCACTGATGGTGTCCAG |
|  |  |  |
| Mfn1 | Forward | CCAGGTACAGATGTCACCACAG |
|  | Reverse | TTGGAGAGCCGCTCATTCACCT |
|  |  |  |
| Mfn2 | Forward | GTGGAATACGCCAGTGAGAAGC |
|  | Reverse | CAACTTGCTGGCACAGATGAGC |
|  |  |  |
| Vapb | Forward | GAAGGTGATGGAAGAGTGCAGG |
|  | Reverse | GCTGTTGCTCGGCATCGCCTT |
|  |  |  |
| Rmdn3 | Forward | CAGAGGCTGCTCTGAAGAAAGG |
|  | Reverse | GCTAAAGCCACTCTGGATGCGT |
|  |  |  |
| Fis1 | Forward | GCTGGTTCTGTGTCCAAGAGCA |
|  | Reverse | GACATAGTCCCGCTGTTCCTCT |
| Bap31 | Forward | CCAGAACAATCCAGGTGCCATG |
|  | Reverse | TGAGAGTCACCAGGCGTCTAAG |
|  |  |  |
| Opa1 | Forward | TCTCAGCCTTGCTGTGTCAGAC |
|  | Reverse | TTCCGTCTCTAGGTTAAAGCGCG |
|  |  |  |
| GAPDH | Forward | CATCACTGCCACCCAGAAGACTG |
|  | Reverse | ATGCCAGTGAGCTTCCCGTTCAG |
|  |  |  |
| DRP1 | Forward | GCGAACCTTAGAATCTGTGGACC |
|  | Reverse | CAGGCACAAATAAAGCAGGACGG |

| **Human primer sequences** | | |
| --- | --- | --- |
| CHOP | Forward | TAAAGATGAGCGGGTGGCAG |
|  | Reverse | CCTTCTTGAACACTCTCTCCTCAG |
|  |  |  |
| DERL1 | Forward | TGCATCTTGGCTACCTGTGG |
|  | Reverse | CCCATCAAAAGCTCCTGAAAGC |
|  |  |  |
| CALR | Forward | TGGACCTCTGGCAGGTCAAG |
|  | Reverse | AGCGTATGCCTCATCGTTGG |
|  |  |  |
| GRP75 | Forward | GGACGTGAGCAGCAGATTGT |
|  | Reverse | CCTTCTTTCGCCGGTCTTCT |
|  |  |  |
| ITP3R | Forward | GCAACCACATCTGGACGCTCTT |
|  | Reverse | AGAAGGCGTTGATGGTGTCCAG |
|  |  |  |
| VDAC1 | Forward | CGGAAGGCAGAAGATGGCTG |
|  | Reverse | GGCTGAGCCTGAGCTTGTAA |
|  |  |  |
| GAPDH | Forward | GTCTCCTCTGACTTCAACAGCG |
|  | Reverse | ACCACCCTGTTGCTGTAGCCAA |
|  |  |  |
| DRP1 | Forward | GATGCCATAGTTGAAGTGGTGAC |
|  | Reverse | CCACAAGCATCAGCAAAGTCTGG |
|  |  |  |
| BiP | Forward | CTGTCCAGGCTGGTGTGCTCT |
|  | Reverse | CTTGGTAGGCACCACTGTGTTC |
|  |  |  |
| MFN1 | Forward | GGTGAATGAGCGGCTTTCCAAG |
|  | Reverse | TCCTCCACCAAGAAATGCAGGC |
|  |  |  |
| MFN2 | forward | ATTGCAGAGGCGGTTCGACTCA |
|  | Reverse | TTCAGTCGGTCTTGCCGCTCTT |
|  |  |  |
| Vapb | forward | AGGTTATGGAAGAATGTAAGAGGC |
|  | Reverse | GTTGCTCTGCACTGTCTTCCTC |
|  |  |  |
| Rmdn3 | Forward | TCACTGAGGAGGTGAGCGAGAA |
|  | Reverse | CAGGTGACAGTCAGCACTCTCA |
|  |  |  |
| Fis1 | Forward | CAAGGAACTGGAGCGGCTCATT |
|  | Reverse | GGACACAGCAAGTCCGATGAGT |
|  |  |  |
| Bap31 | Forward | AGAACAGGAGCCTGAAGGCTGA |
|  | Reverse | AAGCGGTCGTACTCCTTGGTGA |
|  |  |  |

**Supplementary Table 1: Primer sequences (mouse and human) used in qPCR experiments**

| **Case No.** | **Age** | **Gender** | **Cause of death** | **PMI (hrs)** | **Neurol. diagnosis** | **Neuropathol. diagnosis** | **pTDP-43 aggregates** |
| --- | --- | --- | --- | --- | --- | --- | --- |
| *Controls* |  |  |  |  |  |  |  |
| 1 | 70 | M | Heart failure | 7 | - | **Normal** |  |
| 2 | 81 | M | Organ failure | 16 | - | **Normal** |  |
| 3 | 54 | M | Sepsis | 15 | - | **Normal** |  |
| 4 | 67 | M | Cardiac dysrhythmia | 13 | - | **Normal** |  |
| *C9ORF72* |  |  |  |  |  |  |  |
| 1 | 64 | F | Respiratory | 24 | fALS | ***C9ORF72*-ALS** | + |
| 2 | 53 | M | Respiratory | 36 | fALS | ***C9ORF72*-ALS** | + |
| 3 | 68 | M | Assisted suicide | 12 | fALS | ***C9ORF72*-ALS** | + |
| 4 | 69 | M | Unknown | 26 | fALS | ***C9ORF72*-ALS** | + |

**Supplementary Table 2: Demographic description of Control and *C9ORF72*-ALS/FTD cases**

Cases have undergone comprehensive clinical, neuropathological and genetic workup, the latter with targeted next-generation sequencing analysis of *C9ORF72* as well as *TARBP, ALS2, ErbB4, NEK1, MATR3, VCP, SIGMAR1, C19ORF12, OPTN, HNRNPA1, DAO, SPG11, FUS, GRN, PNPLA6, SOD1, CHCHD10, NEFH* and *UBQLN2.* For details see:

**Reference:** Månberg A, Skene N, Sanders F, Trusohamn M, Remnestål J, Szczepińska A, Aksoylu IS, Lönnerberg P, Ebarasi L, Wouters S, Lehmann M, Olofsson J, von Gohren Antequera I, Domaniku A, De Schaepdryver M, De Vocht J, Poesen K, Uhlén M, Anink J, Mijnsbergen C, Vergunst-Bosch H, Hübers A, Kläppe U, Rodriguez-Vieitez E, Gilthorpe JD, Hedlund E, Harris RA, Aronica E, Van Damme P, Ludolph A, Veldink J, Ingre C, Nilsson P, Lewandowski SA.

Altered perivascular fibroblast activity precedes ALS disease onset. Nat Med. 2021 Apr;27(4):640-646. doi: 10.1038/s41591-021-01295-9.

| Rabbit | Anti-GRP75 | ABCAM | 1:100 |
| --- | --- | --- | --- |
| Goat | Anti-GRP75 | R&D system | 1:100 |
| Mouse | Anti-PolyGA | Merk Millipore | 1:100 |
| Rabbit | Anti-pTDP-43 | CosmoBio | 1:100 |

**Supplementary Table 3: Antibodies used for post-mortem human tissue staining**
